# Supplementary material for: Multitask benchmarking of single-cell multimodal omics integration methods
Source: Nat Methods. 2025 Oct 13;22(11):2449–60. doi: 10.1038/s41592-025-02856-3 (PMC12615258; doi:10.1038/s41592-025-02856-3)
Supplement: Supplementary file 1 — Supplementary methods, algorithms and Figs. 1–13. [file 41592_2025_2856_MOESM1_ESM.pdf]

nature methods

Supplementary information

https://doi.org/10.1038/s41592-025-02856-3

Multitask benchmarking of single-cell multimodal omics integration methods

In the format provided by the authors and unedited

Supplementary Notes

Supplementary methods

Summary of integration methods in each category and task

The following criteria are used for selecting integration methods for benchmarking:

- ***Reasonable speed and memory requirements.*** Methods characterised by excessively slow speeds or high memory consumption are excluded.
- ***High reproducibility.*** Only methods demonstrating consistent results when applied to varied datasets, ensuring robustness and reliability, are included in the benchmarking process.
- ***Comprehensive documentation and tutorials.*** We exclude methods lacking detailed descriptions of input data and/or running instructions.
- ***Active maintenance.*** Preference is given to methods that are maintained and updated.
- ***Joint latent space or graph generation.*** We exclude methods that do not generate a joint latent space or graph except those for spatial registration, as this is an overwhelmingly common capability among most methods.
- ***Broad applicability.*** Methods that are designed exclusively for specific types of data, such as CyTOF, and are not broadly applicable, are not included.
- ***Platform accessibility.*** We focus on methods compatible with Python or R.
- ***Ease of installation.*** Methods requiring special computing resources or complicated installation processes are disregarded.

The above selection criteria lead to triage of 40 integration methods.

Experimental datasets

The following are details of the raw datasets used for benchmarking.

Datasets for vertical integration

D1: consists of samples generated by the "Cambridge Medical Centre" from data source 1. It contains paired RNA and ADT data modalities of 30,313 cells from 17 cell types with 24,737 genes and 192 proteins.

D2: consists of samples generated by the "NCL Medical Centre" from data source 1. It contains paired RNA and ADT data modalities of 64,262 cells from 18 cell types, with 24,737 genes and 192 proteins.

D3: consists of "HD1_Adult" samples from data source 2. It contains paired RNA and ADT data modalities of 8,650 cells from 30 cell types with 33,538 genes and 189 proteins.

D4: consists of "HD2_Adult" samples from data source 2. It contains paired RNA and ADT data modalities of 9,538 cells from 29 cell types with 33,538 genes and 189 proteins.

D5: consists of "HD3_Adult" samples from data source 2. It contains paired RNA and ADT data modalities of 10,423 cells from 30 cell types with 33,538 genes and 189 proteins.

D6: consists of “Batch1” samples from data source 3. It contains paired RNA and ADT data modalities of 67,090 cells from 31 cell types with 33,538 genes and 228 proteins.

D7: consists of “Batch2” samples from data source 3. It contains paired RNA and ADT data modalities of 94,674 cells from 31 cell types with 33,538 genes and 228 proteins.

D8: consists of “batch1” samples from data source 5. It contains paired RNA and ADT data modalities of 14,468 cells from 27 cell types with 17,009 genes and 25 proteins.

D9: consists of “batch2” samples from data source 5. It contains paired RNA and ADT data modalities of 16,204 cells from 27 cell types with 17,009 genes and 25 proteins.

D10: consists of “PBMC” samples from data source 6. It contains paired RNA and ADT data modalities of 7,108 cells from 11 cell types with 33,514 genes and 52 proteins.

D11: consists of “Lung” samples from data source 6. It contains paired RNA and ADT data modalities of 3,362 cells from 14 cell types with 33,514 genes and 52 proteins.

D12: consists of all samples from data source 7. It contains paired RNA and ATAC data modalities of 34,774 cells from 23 cell types with 23,296 genes and 344,592 ATAC peaks.

D13: consists of “AdBrainCortex” samples from data source 8. It contains paired RNA and ATAC data modalities of 10,309 cells from 23 cell types with 33,160 genes and 244,544 ATAC peaks.

D14: consists of “P0_BrainCortex” samples from data source 8. It contains paired RNA and ATAC data modalities of 5,081 cells from 19 cell types with 19,322 genes and 229,429 ATAC peaks.

D15: consists of all samples from data source 11. It contains paired RNA and ATAC data modalities of 11,787 cells from 13 cell types with 36,601 genes and 108,344 ATAC peaks.

D16: consists of “Adol1” samples from data source 12. It contains paired RNA, ATAC data modalities of 3,188 cells from 14 cell types with 30,201 genes, 304,034 ATAC peaks.

D17: consists of “Adult1” samples from data source 12. It contains paired RNA, ATAC data modalities of 1,541 cells from 11 cell types with 30,201 genes, 304,034 ATAC peaks.

D18: consists of “Child1” samples from data source 12. It contains paired RNA, ATAC data modalities of 5,418 cells from 13 cell types with 30,201 genes, 304,034 ATAC peaks.

D19: consists of “EaFet1” samples from data source 12. It contains paired RNA, ATAC data modalities of 2,801 cells from 12 cell types with 30,201 genes, 304,034 ATAC peaks.

D20: consists of “Inf1” samples from data source 12. It contains paired RNA, ATAC data modalities of 2,723 cells from 14 cell types with 30,201 genes, 304,034 ATAC peaks.

D21: consists of “LaFet1” samples from data source 12. It contains paired RNA, ATAC data modalities of 3,102 cells from 12 cell types with 30,201 genes, 304,034 ATAC peaks.

D22: consists of “Control” samples from data source 14. It contains paired RNA, ADT, and ATAC data modalities of 7,624 cells from 26 cell types with 36,495 genes, 210 proteins, and 68,963 ATAC peaks.

D23: consists of “Stim” samples from data source 14. It contains paired RNA, ADT, and ATAC data modalities of 6,139 cells from 27 cell types with 36,495 genes, 210 proteins, and 68,963 ATAC peaks.

Datasets for diagonal integration

D24: consists of unpaired RNA and ATAC data modalities from data source 7. Both modalities contain 34,774 cells from 23 cell types. The RNA data modality contains 23,296 genes and the ATAC data modality contains 344,592 peaks.

D25: consists of unpaired RNA and ATAC data modalities of ‘AdBrainCortex’ samples from data source 8. Both modalities contain 10,309 cells from 23 cell types. The RNA data modality contains 33,160 genes and the ATAC data modality contains 244,544 peaks.

D26: consists of unpaired RNA and ATAC data modalities of ‘P0_BrainCortex’ samples from data source 8. Both modalities contain 5,081 cells from 19 cell types. The RNA data modality contains 19,322 genes and the ATAC data modality contains 229,429 peaks.

D27: consists of unpaired RNA and ATAC data modalities from data source 11. Both modalities contain 11,787 cells from 13 cell types. The RNA data modality contains 36,601 genes and the ATAC data modality contains 108,344 peaks.

D28: consists of unpaired RNA and ATAC data modalities from data source 14. The RNA data modality of “Control” samples contains 7,624 cells from 26 cell types with 36,495 genes and the ATAC data modality contains 6,139 cells from 27 cell types with 68,963 peaks.

D29: consists of unpaired RNA and ATAC data modalities from data source 15. The RNA data modality contains 6,314 cells from 13 cell types with 36,601 genes and the ATAC data modality contains 6,548 cells from 12 cell types with 94,986 peaks.

D30: consists of unpaired RNA and ATAC data modalities from data source 11 and 14. The RNA data modality extracted from data source 11 contains 11,787 cells from 13 cell types with 36,601 genes. The ATAC data modality extracted from the ‘Control’ samples of data source 14 contains 6,139 cells from 26 cell types with 68,963 peaks.

D31: consists of unpaired RNA and ATAC data modalities from two different sources including “Control” samples of source 14 and “batch1” samples of source 15. The RNA data modality contains 7,624 cells from 26 cell types with 36,495 genes and ATAC data modality contains 6,314 cells from 13 cell types with 99,658 peaks.

D32: consists of unpaired RNA and ATAC data modalities of “E13” samples from data source 21. The RNA data modality contains 2,187 cells from 14 cell types with 17,058 genes and the ATAC data modality contains 2,187 cells from 14 cell types with 32,437 peaks.

D33: consists of unpaired RNA and ATAC data modalities of “P22” brain from data source 21. The RNA data modality contains 9,215 cells from 11 cell types with 22,914 genes and the ATAC data modality contains 9,215 cells from 11 cell types with 121,068 peaks.

D34: consists of 13 paired RNA and ATAC data modalities from data source 9, which are split into 26 unpaired batches for analysis. Both data modalities contain identical numbers of cells (i.e. 6,224, 6,740, 4,279, 4,220, 6,111, 4,895, 6,781, 4,325, 1,679, 1,771, 8,023, 9,876, and 4,325) and numbers of cell types (i.e. 21, 21, 19, 20, 17, 16, 21, 20, 18, 18, 19, 19, and 18) across the 13 original batches. The RNA data modality contains 13,431 genes and the ATAC data modality contains 116,490 peaks.

D35: consists of 9 paired RNA and ATAC data modalities from data source 10, which are split into 18 unpaired batches. Both data modalities contain identical numbers of cells (i.e. 8,343, 10,968, 3,977, 6,301, 5,467, 9,584, 7,360, 3,822, and 5,204) and numbers of cell types (i.e. 32, 34, 24, 31, 31, 31, 34, 26, and 32) across the 9 original batches. The RNA data modality contains 32,285 genes and the ATAC data modalities contain 162,669, 156,487, 172,779, 169,478, 159,345, 185,545, 186494, 157,105, 170,504 peaks, respectively, and 263,550 consolidated peaks.

D36: consists of 2 paired RNA and ATAC data modalities from data source 14, which are split into 4 unpaired batches. Batch 1 and 3 from "Control" samples both contain 7,624 cells from 26 cell types, and batch 2 and 4 from "Stim" samples both contain 6,139 cells from 27 cell types. The RNA data modality contains 36,495 genes and the ATAC data modality contains 68,963 peaks.

D37: consists of 3 batches of unpaired RNA and ATAC data modalities from data source 15, which are split into 6 unpaired batches. Both data modalities contain identical numbers of cells (i.e. 6,314, 6,548, and 6,547) and numbers of cell types of 13, 12, 13 across the 3 original batches. The RNA data modalities contain 36,601, 36,601, 36,601 genes and the ATAC data modalities contain 99,658, 94,986, 83,234 peaks, respectively, and 109,087 consolidated peaks.

Datasets for mosaic integration

D38: sourced from data source 1, consists of 3 data batches: one with only RNA modality (24,737 genes), a second with both RNA (24,737 genes) and ADT (192 proteins) modalities, and a third with only ADT modality (192 proteins). It contains 30,313, 64,262, and 30,313 cells from 17, 18, and 17 cell types, respectively.

D39: sourced from data source 2, consists of 3 data batches: one with only RNA modality (33,538 genes), a second with both RNA (33,538 genes) and ADT (189 proteins) modalities, and a third with only ADT modality (189 proteins). It contains 8,650, 9,538, and 10,423 cells from 30, 29, and 30 cell types, respectively.

D40: sourced from data source 3, consists of 3 data batches: one with only RNA modality (33,538 genes), a second with both RNA (33,538 genes) and ADT (228 proteins) modalities, and a third with only ADT modality (228 proteins). It contains 67,090, 94,674, and 67,090 cells from 31, 31, and 31 cell types, respectively.

D41: sourced from data source 5, consists of 3 data batches: one with only RNA modality (17,009 genes), a second with both RNA (17,009 genes) and ADT (25 proteins) modalities, and a third with only ADT modality (25 proteins). It contains 14,468, 16,204, and 14,468 cells from 27, 27, and 27 cell types, respectively.

D42: sourced from data source 7, consists of 3 data batches: one with only RNA modality (23,296 genes), a second with both RNA (23,296 genes) and ATAC (344,592 peaks) modalities, and a third with only ATAC modality (344,592 peaks). It contains 34,774 cells from 23 cell types for all 3 batches.

D43: sourced from data source 8, consists of 3 data batches: one with only RNA modality (33,160 genes), a second with both RNA (33,160 genes) and ATAC (244,544 peaks) modalities, and a third with only ATAC modality (244,544 peaks). It contains 10,309 cells from 23 cell types for all 3 batches.

D44: sourced from data source 8, consists of 3 data batches: one with only RNA modality (19,322 genes), a second with both RNA (19,322 genes) and ATAC (229,429 peaks) modalities, and a third with only ATAC modality (229,429 peaks). It contains 5,081 cells from 19 cell types for all 3 batches.

D45: sourced from data source 11, consists of 3 data batches: one with only RNA modality (36,601 genes), a second with both RNA (36,601 genes) and ATAC (108,344 peaks) modalities, and a third with only ATAC modality (108,344 peaks). It contains 11,787 cells from 13 cell types for all 3 batches.

D46: sourced from data sources 4 and 9, consists of 3 data batches: one with paired RNA (13,953 genes) and ADT (134 proteins) modalities, a second with paired RNA (13,431 genes) and ATAC (116,490 peaks) modalities, and a third with only RNA modality (13,953 genes). It contains 10,465, 9,876, and 5,227 cells from 38, 19, and 37 cell types, respectively.

D47: sourced from data sources 4 and 9, consists of 3 data batches: one with paired RNA (13,953 genes) and ADT (134 proteins) modalities, a second with paired RNA (13,431 genes) and ATAC (116,490 peaks) modalities, and a third with only RNA modality (13,953 genes). It contains 11,473, 8,023, and 11,035 cells from 34, 19, and 36 cell types, respectively.

D48: sourced from data sources 4 and 9, consists of 3 data batches: one with paired RNA (13,953 genes) and ADT (134 proteins) modalities, a second with paired RNA (13,431 genes) and ATAC (116,490 peaks) modalities, and a third with only ATAC modality (116,490 peaks). It contains 9,521, 4,895, and 9,876 cells from 37, 16, and 19 cell types, respectively.

D49: sourced from data sources 4 and 9, consists of 3 data batches: one with paired RNA (13,953 genes) and ADT (134 proteins) modalities, a second with paired RNA (13,431 genes) and ATAC (116,490 peaks) modalities, and a third with only ADT modality (134 proteins). It contains 4,978, 4,325, and 5,227 cells from 41, 20, and 37 cell types, respectively.

D50: sourced from data sources 4 and 9, consists of 5 data batches: one with paired RNA (13,953 genes) and ADT (134 proteins) modality, a second with paired RNA (13,431 genes) and ATAC (116,490 peaks) modality, a third with only RNA modality (13,953 genes), a fourth with only ADT modality (134 proteins), and a fifth with only ATAC modality (116,490 peaks). It contains 10,465, 9,876, 11,473, 11,035, and 8,023 cells from 38, 19, 34, 36, and 19 cell types, respectively.

Datasets for cross integration

D51: consists of 2 batches of CITE-seq data generated from data source 1. Batch 1 consists of samples from the "Cambridge Medical Centre" and contains 30,313 cells from 17 cell types and batch 2 consists of samples from the "NCL Medical Centre" and contains 64,262 cells from 18 cell types. Both batches consist of 24,737 genes and 192 proteins.

D52: consists of 3 batches of CITE-seq data generated from data source 2. Batch 1 consists of samples from "HD1_Adult" and contains 8,650 cells from 30 cell types; batch 2 consists of samples from "HD2_Adult" and contains 9,538 cells from 29 cell types; and batch 3 consists of samples from "HD3_Adult" and contains 10,423 cells from 30 cell types. All batches consist of 33,538 genes and 189 proteins.

D53: consists of 2 batches of CITE-seq data generated from data source 3. Batch 1 consists of samples from "batch1" and contains 67,090 cells from 31 cell types and batch 2 consists of samples from "batch2" and contains 94,674 cells from 31 cell types. Both batches consist of 33,538 genes and 228 proteins.

D54: consists of 12 batches of CITE-seq data generated from data source 4. The 12 batches contain 5,227, 4,978, 6,106, 10,465, 5,584, 9,122, 9,521, 11,035, 11,473, 5,456, 3,929, and 7,365 cells, which correspond to 37, 41, 36, 38, 30, 34, 37, 36, 34, 39, 33, and 36 cell types, respectively. All batches share the same set of features including 13,953 genes and 134 proteins.

D55: consists of 2 batches of CITE-seq data generated from data source 5. Batch 1 consists of samples from "batch1" and contains 14,468 cells from 27 cell types, and batch 2 consists of samples from "batch2" and contains 16,204 cells from 27 cell types. Both batches share the same set of features including 17,009 genes and 25 proteins.

D56: consists of 13 batches of 10x multiome data generated from data source 9. The 13 batches contain 6,224, 6,740, 4,279, 4,220, 6,111, 4,895, 6,781, 4,325, 1,679, 1,771, 8,023, 9,876, and 4,325 cells, which correspond to 21, 21, 19, 20, 17, 16, 21, 20, 18, 18, 19, 19, and 18 cell types, respectively. All batches share the same set of features including 13,431 genes and 11,6490 ATAC peaks.

D57: consists of 9 batches of 10x multiome data generated from data source 10. The 9 batches contain 8,343, 10,968, 3,977, 6,301, 5,467, 9,584, 7,360, 3,822, and 5,204 cells, which correspond to 32, 34, 24, 31, 31, 31, 34, 26, and 32 cell types, respectively. All batches share the same set of RNA features with 32,285 genes while the ATAC data modalities contain 162,669, 156,487, 172,779, 169,478, 159,345, 185,545, 186,494, 157,105, 170,504 peaks (263,550 consolidated peaks), respectively.

D58: consists of 2 batches of ASAP-seq data generated from data source 13. Batch 1 contains 3,517 cells from 4 cell types, and batch 2 contains 4,849 cells from 4 cell types. ATAC data modalities contain 21,994, 26,808 peaks (21,344 common peaks), while ADT data modalities contain 227, 227 proteins.

D59: consists of 2 batches of DOGMA-seq data generated from data source 14. Batch 1 contains 7,624 cells from 26 cell types, and batch 2 contains 6,139 cells from 27 cell types. Both batches share the same set of features including 36,495 genes, 210 proteins, and 68,963 ATAC peaks.

Datasets for spatial integration

D60: consists of 12 batches of spatial transcriptomics data extracted from mouse squamous cell carcinoma from data source 16. Each batch of data contains RNA expression and spatial coordinates, generated using the Visium platform. The numbers of genes in each batch are 12,453, 12,675, 12,830, 8946, 9759, 8287, 10,319, 10,176, 8,399, 7,659, 9,949, and 8,359, and the corresponding numbers of cells are 666, 645, 638, 584, 517, 517, 1,125, 1,035, 828, 545, 619, and 460. The number of spot labels in each batch are 12, 12, 12, 8, 8, 8, 12, 12, 12, 8, 8, 7.

D61: includes 12 batches of spatial transcriptomics data extracted from the human dorsolateral prefrontal cortex from data source 17. Each batch of data contains RNA expression and spatial coordinates, generated using the Visium platform. The number of genes in each batch are 9,948, 9,256, 10,486, 10,104, 10,461, 9,972, 10,996, 10,609, 11,381, 12,381, 10,629, and 10,778, and the corresponding numbers of cells are 4,220, 4,379, 4,786, 4,593, 3,635, 3,483, 4,092, 3,888, 3,611, 3,635, 3,566, and 3,431. The number of spot labels in each batch are 7, 7, 7, 7, 5, 5, 5, 5, 7, 7, 7, 7.

D62: includes 2 batches of spatial transcriptomics data of human breast cancer extracted from data source 18. Each batch of data contains RNA expression and spatial coordinates, generated using Xenium platform. The number of cells in each batch are 167,780, 118,752, and the 2 batches both have 541 genes and 20 spot labels.

D63: includes 4 batches of spatial transcriptomics data of drosophila embryos extracted from data source 19. Each batch of data contains RNA expression and spatial coordinates, generated using Stereo-seq. The number of cells in each batch are 925, 1,272, 1,263, 1,248, and the corresponding numbers of genes and cell types are consistently 13,668 and 10, respectively.

D64: includes 8 batches of spatial transcriptomics data of mouse brain extracted from data source 20. Each batch of data contains RNA expression and spatial coordinates, generated using MERFISH. The number of cells in each batch are 9,571, 11,047, 10,890, 9,665, 9,617, 8,823, 15,694, 24,790 and the corresponding numbers of genes and cell types are consistently 374 and 13, respectively.

Simulation datasets

Datasets for vertical integration

SD1: contains paired RNA and ADT data modalities of 3,000 cells from 3 cell types with 5,000 genes and 200 proteins.

SD2: contains paired RNA and ADT data modalities of 5,000 cells from 5 cell types with 3,000 genes and 200 proteins.

SD3: contains paired RNA and ATAC data modalities of 3,000 cells from 3 cell types with 2,000 genes and 3,000 peaks.

SD4: contains paired RNA and ATAC data modalities of 5,000 cells from 5 cell types with 1,000 genes and 2,000 peaks.

SD5: contains paired RNA, ADT, and ATAC data modalities of 3,000 cells from 3 cell types with 2,000 genes, 200 proteins, and 3,000 ATAC peaks.

SD6: contains paired RNA, ADT, and ATAC data modalities of 5,000 cells from 5 cell types with 1,000 genes, 200 proteins, and 2,000 ATAC peaks.

Datasets for diagonal integration

SD7: consists of unpaired RNA and ATAC data modalities. Both modalities contain 3,000 cells from 3 cell types. The RNA data modality contains 2,000 genes and the ATAC data modality contains 3,000 peaks that correspond to 2,000 gene activity scores.

SD8: consists of unpaired RNA and ATAC data modalities. Both modalities contain 5,000 cells from 5 cell types. The RNA data modality contains 1,000 genes and the ATAC data modality contains 2,000 peaks that correspond to 1,000 gene activity scores.

SD9: consists of 2 batches of paired RNA and ATAC data, which are split into 4 unpaired batches. The first original batch contains 2,003 cells from 3 cell types and the second original batch contains 1,997 cells from 3 cell types. For both batches, the RNA data modalities contain 2,000 genes and the ATAC data modalities contain 3,000 peaks that correspond to 2,000 gene activity scores.

SD10: consists of 2 batches of paired RNA and ATAC data, which are split into 4 unpaired batches. The first original batch contains 2,514 cells from 5 cell types and the second original batch contains 2,486 cells from 5 cell types. For both batches, the RNA data modalities contain 2,000 genes and the ATAC data modalities contain 5,000 peaks that correspond to 2,000 gene activity scores.

Datasets for mosaic integration

SD11: consists of 3 data batches where the first with only RNA modality (5,000 genes), the second with both RNA (5,000 genes) and ADT (200 proteins) modalities, and the third with only ADT modality (200 proteins). It contains 3,000 cells from 3 cell types for all 3 batches.

SD12: consists of 3 data batches where the first with only RNA modality (3,000 genes), the second with both RNA (3,000 genes) and ADT (200 proteins) modalities, and the third with only ADT modality (200 proteins). It contains 5,000 cells from 5 cell types for all 3 batches.

SD13: consists of 3 data batches where the first with only RNA modality (2,000 genes), the second with both RNA (2,000 genes) and ATAC (3,000 peaks) modalities, and the third with only ATAC modality (3,000 peaks). It contains 3,000 cells from 3 cell types for all 3 batches.

SD14: consists of 3 data batches where the first with only RNA modality (1,000 genes), the second with both RNA (1,000 genes) and ATAC (2,000 peaks) modalities, and the third with only ATAC modality (2,000 peaks). It contains 5,000 cells from 5 cell types for all 3 batches.

Datasets for cross integration

SD15: consists of 2 batches of CITE-seq data. The 2 batches contain 2,003 and 1,997 cells from 3 cell types, respectively. Both batches share the same set of features including 2,000 genes and 200 proteins.

SD16: consists of 2 batches of CITE-seq data. The 2 batches contain 2,514 and 2,486 cells from 5 cell types, respectively. Both batches share the same set of features including 2,000 genes and 200 proteins.

SD17: consists of 2 batches of paired RNA and ATAC data. The 2 batches contain 2,003 and 1,997 cells from 3 cell types, respectively. Both batches share the same set of features including 2,000 genes and 3,000 ATAC peaks.

SD18: consists of 2 batches of paired RNA and ATAC data. The 2 batches contain 2,514 and 2,486 cells from 5 cell types, respectively. Both batches share the same set of features including 2,000 genes and 5,000 ATAC peaks.

SD19: consists of 2 batches of paired ADT and ATAC data. The 2 batches contain 2,003 and 1,997 cells from 3 cell types, respectively. Both batches share the same set of features including 200 proteins and 3,000 ATAC peaks.

SD20: consists of 2 batches of paired ADT and ATAC data. The 2 batches contain 2,514 and 2,486 cells from 5 cell types, respectively. Both batches share the same set of features including 200 proteins and 5,000 ATAC peaks.

SD21: consists of 2 batches of paired RNA, ADT, and ATAC data. The 2 batches contain 2,003 and 1,997 cells from 3 cell types, respectively. Both batches share the same set of features including 2,000 genes, 200 proteins and 3,000 ATAC peaks.

SD22: consists of 2 batches of paired RNA, ADT, and ATAC data. The 2 batches contain 2,514 and 2,486 cells from 5 cell types, respectively. Both batches share the same set of features including 2,000 genes, 200 proteins and 5,000 ATAC peaks.

Single-cell multimodal omics data integration methods

M1: totalVI*[1] *(v1.1.2). We create the ‘anndata’ objects and employed the ‘scvi.model.TOTALVI.setup_anndata’, ‘scvi.model.TOTALVI’, and ‘train’ functions to train the totalVI model as described in the tutorial (https://docs.scvi-tools.org/en/stable/tutorials/notebooks/multimodal/totalVI.html). Finally, we employ the ‘get_latent_representation’ function to obtain the integrated embeddings. Besides, for the imputation purpose, we set ‘include_protein_background=True’ in the ‘get_normalized_expression’ function to obtain the imputed ADT.

M2: sciPENN*[2] *(v1.0.0). We use ‘sciPENN_API’ and ‘train’ functions to build and train the model following the tutorial (https://github.com/jlakkis/sciPENN). Then, we use the ‘embed’ function to obtain the embeddings and use the ‘predict’ function to obtain the imputed modality.

M3: Concerto*[3] *(github version ab1fc7f). We construct the model using the ‘multi_embedding_attention_transfer’ function. The ‘concerto_train_multimodal’ function is then used to train the model with default settings (https://github.com/melobio/Concerto - reproducibility). Finally, we obtain the embeddings through the ‘concerto_test_multimodal’ function.

M4: scMSI*[4] *(github version dffcbb2). As described in the tutorial (https://github.com/ChengmingZhang-CAS/scMSI-master/tree/main), we construct the model using the 'SCMSIRNAProtein' function. Then, we train the model using the 'train' function . To obtain the integrated embeddings, we concatenate the embeddings from the RNA and ADT data.

M5: Matilda*[5] *(github version 7d71480). The count matrices along with the cell type labels, are used as input. As described in the authors' protocol (https://github.com/PYangLab/Matilda), we use 'main_matilda_train.py' to train the model with default parameters. Then, we use 'main_matilda_task.py' with 'dim_reduce=True' to obtain embeddings and set 'fs=True' to obtain the importance score for feature selection.

M6: MOFA+*[6] *(v1.6.0). As described in the vignette (https://raw.githack.com/bioFAM/MOFA2_tutorials/master/R_tutorials/getting_started_R.html), we use the 'create_mofa' function, with a list of batches to establish the MOFA object. Then, we train the model using the 'run_mofa' function, following the standard pipeline and parameters. Finally, we extract 'expectations$Z' and 'intercepts$group1' from the resulting object to obtain the embeddings and importance score for feature selection, respectively.

M7: Multigrate*[7] *(v0.0.2). Following the authors' instructions (https://github.com/theislab/multigrate_reproducibility), we employ the 'data.organize_multiome_anndatas', 'model.MultiVAE.setup_anndata', 'model.MultiVAE', and 'train' functions to construct and train the model. Finally, we use the 'get_latent_representation' function to obtain the embeddings.

M8: UINMF*[8] *(v2.0.1). Following the authors' vignette (https://github.com/welch-lab/liger/blob/master/vignettes/articles/UINMF_vignette.Rmd), we apply functions such as 'createLiger', 'normalize', 'selectGenes', 'scaleNotCenter', and 'runUINMF' in the 'rliger' package to perform data integrations. Finally, we extract 'H' from the resulting object to obtain the embeddings.

M9: scMoMaT*[9] *(v0.2.2). We first compile a list comprising multi-batch multi-modal data into the 'preprocess' function. Then, this data is fed into the 'scmomat_model' and 'train_func' to train the model with default settings (https://github.com/PeterZZQ/scMoMaT). Finally, we use 'extract_cell_factors' and 'calc_post_graph' functions to extract the graphs, and the 'extract_marker_scores' function to obtain feature importance scores respectively.

M10: Seurat (WNN)*[10] *(v5.0.2). As described in the authors' vignette (https://satijalab.org/seurat/articles/weighted_nearest_neighbor_analysis.html), we calculate the closest neighbours in the dataset using the 'FindMultiModalNeighbors' function with default settings. Finally, we extract 'weighted.nn' and 'wknn' from the resulting object to obtain the graphs.

M11: scMM*[11] *(github version c5c8579). We use the 'main.py' file to train the model to obtain the embeddings as described in the authors' protocol (https://github.com/kodaim1115/scMM/tree/master). Specifically, we set the 'model' parameter based on the input data combination while keeping other parameters as default. Finally, we employ the 'get_latent' function to obtain the embeddings and use the 'predict' function to impute missing modalities.

M12: scMDC*[12] *(github version 43b0c3a). Following the authors' protocol (https://github.com/xianglin226/scMDC), we build the model using the 'scMultiClusterBatch' function with 'n_batch' as the actual number of batches. Then, the model is trained using the 'pretrain_autoencoder' function with the default parameters. Finally, we extract the embeddings through the 'encodeBatch' function.

M13: moETM*[13] *(github number c2eaa97). moETM is capable of being applied to bi-modality combinations such as RNA+ADT or RNA+ATAC. The count matrices from all modalities are used as input. Following (https://github.com/manqizhou/moETM), we first preprocess the data, and then use 'build_moETM' and 'Train_moETM' functions to build and train the model with default settings. Finally, we extract 'delta' from the resulting object to obtain the embeddings. For imputation, we use the 'Trainer_moETM_for_cross_prediction' and 'Train_moETM_for_cross_prediction' functions.

M14: VIMCCA*[14] *(v0.5.6). We use the 'log1p' and 'scale' function to process the input data, followed by the 'vimcca.fit_integration' function to train the model with default parameters (https://github.com/jhu99/scbean). The output from 'vimcca.fit_integration' is the integrated embedding.

M15: iPOLNG*[15] *(v0.0.2). The count matrices with 5,000 highly variable genes and 20,000 highly variable peaks are used as input (as suggested by the authors). We use the 'iPoLNG' function with the default parameters to train the model (https://github.com/cuhklinlab/iPoLNG). Finally, we extract 'Ls_est' from the resulting object to obtain the embeddings.

M16: MIRA*[16] *(v2.1.0). We employ 'utils.make_joint_representation' to construct the joint representation space with default parameters (https://github.com/cistrome/MIRA). Finally, we calculate the integrated graph via the 'neighbours' function from the scanpy package on 'X_joint_umap_features' from the resulting AnnaData object.

M17: UnitedNet*[17] *(github version 3689da8). The count matrices along with the cell type labels, are used as input. We build the model using the 'UnitedNet' function. We adjust the 'input' dimension to match the actual dimension for the 'encoder', 'decoder', and 'discriminators', and set the 'output' in the 'clusters' to reflect the true number of cell types in the configuration. The model is then trained using the 'train' and 'finetune' functions with default settings (https://github.com/LiuLab-Bioelectronics-Harvard/UnitedNet). Subsequently, we use the 'infer' function to obtain the embeddings and 'predict' function to impute the missing modality in the test data.

M18: scMVP*[18] *(github version fc61e4d). The count matrices from paired RNA and ATAC are used as input, followed by standard preprocessing on both modalities. We build the model using the 'Multi_VAE_Attention' function. The model is then trained using the 'MultiTrainer' and 'train' functions with default settings (https://github.com/bm2-lab/scMVP). Subsequently, we use the 'create_posterior' function to create posterior from the trained model and 'get_latent' function to obtain the embeddings.

M19: scBridge*[19] *(github version ff17561). We use the 'Net' function and 'run' function to construct and train the network with the default parameters (https://github.com/XLearning-SCU/scBridge). Subsequently, we extract the 'Embedding' from the resulting object.

M20: Portal*[20] *(v1.0.2). We employ the 'model.Model' function to construct the model. Subsequently, we use the 'preprocess' function for data preparation, the 'train' function for model training, and the 'eval' function to obtain the integrated embeddings of the cells. All these steps are executed using default settings (https://github.com/YangLabHKUST/Portal).

M21: SCALEX*[21] *(v1.0.2). We use the 'SCALEX' function to train the model (https://github.com/jsxlei/SCALEX). Then, we extract the 'latent' from the resulting object to obtain the integrated embeddings.

M22: VIPCCA*[22] *(v0.2.7). We use the 'preprocessing' function to process the input data, followed by the 'VIPCCA' function to train the model with default parameters (https://github.com/jhu99/vipcca). Finally, we use the 'fit_integrate' function to obtain the integrated embedding.

M23: Seurat v3*[23] *(v5.0.2). We identify the anchors and co-embeddings using the 'VariableFeatures', 'GetAssayData', 'FindTransferAnchors', 'merge', 'ScaleData', and 'RunPCA' functions for integration (https://satijalab.org/seurat/articles/seurat5_atacseq_integration_vignettel). Specifically, the reduction parameter in the 'FindTransferAnchors' function is set to 'cca' by default. The integrated embeddings are extracted using the 'cell.embeddings' function.

M24: MultiMAP*[24] *(github version 681e608). The RNA count matrices and ATAC counts, which feature both peaks and gene activity scores, are utilised as inputs. Following the authors' recommendations (https://nbviewer.org/github/Teichlab/MultiMAP/blob/master/examples/tutorial.ipynb), we utilise the 'Integration' function to integrate the data with the default parameters. Then, we obtain the integrated embedding from the 'X_multimap' attribute.

M25: Seurat v5*[25] *(v5.0.2). Following the authors' vignette (https://satijalab.org/seurat/articles/seurat5_integration_bridge.html), we utilise the 'PrepareBridgeReference', 'FindBridgeTransferAnchors', and 'MapQuery' functions to perform the integration. Finally, we extract 'cell.embeddings' to obtain the integrated embeddings.

M26: sciCAN*[26] *(github version ad71bba). The count matrices from both RNA and ATAC (gene activity scores) are used as inputs. Then, normalisation, log-transformation, and standard scaling are applied to both modalities. Following (https://github.com/rpmccordlab/sciCAN) and the authors' recommendations, we deploy the 'Cycle_train_wolabel' function with 200 epochs, while keeping other parameters as the default, to train our model. Then, we use the 'FeatureExtractor' function to extract the integrated embeddings from the model.

M27: Conos*[27] *(v1.5.2). Following the authors' instructions (https://htmlpreview.github.io/?https://raw.githubusercontent.com/kharchenkolab/conos/main/doc/integrating_rnaseq_atacseq.html), the count data from RNA and ATAC, presented as a list of matrices, is used as input for the Conos method. We perform preprocessing using the 'basicP2proc' function in the pagoda2 package. Then, we utilise functions such as 'new', 'buildGraph', 'findCommunities', and 'embedGraph' in the Conos package to perform the integration. Finally, we extract the 'graph_connectivities' to obtain the integrated graph.

M28: iNMF*[28] *(v2.0.1). We apply functions such as ‘createLiger’, ‘normalize’, ‘selectGenes’, ‘scaleNotCenter’, ‘runIntegration’, and ‘quantile_norm’ to align the RNA and ATAC data with the default settings (https://github.com/welch-lab/liger/blob/master/vignettes/articles/Integrating_scRNA_and_scATAC_data.Rmd). Finally, we extract ‘H.norm’ to obtain the integrated embeddings.

M29: online iNMF*[29] *(v2.0.1). Following the authors’ protocol (https://github.com/welch-lab/liger/blob/master/vignettes/articles/online_iNMF_tutorial.Rmd), we apply functions such as ‘createLiger’, ‘normalize’, ‘selectGenes’, ‘scaleNotCenter’, ‘runIntegration’, and ‘quantile_norm’ to integrate the RNA and ATAC data. Finally, we extract the embeddings in ‘H.norm’ to obtain the integrated embeddings.

M30: scJoint*[30] *(github version cbbfa5d). The count matrices along with the cell type labels, are used as input. We use the ‘TrainingProcessStage1’, ‘KNN’, and ‘TrainingProcessStage3’ functions to train the model as described in the tutorial (https://github.com/SydneyBioX/scJoint). Then, we use the encoder of the model to obtain the integrated embeddings.

M31: GLUE*[31] *(v0.3.2). The RNA count matrices and ATAC counts, which feature peaks, are utilised as inputs. Following the authors’ tutorial (https://github.com/gao-lab/GLUE), we construct a graph via the ‘get_gene_annotation’ function and build an RNA-anchored guidance graph with graph validation using the ‘check_graph’ function. Then, we use the ‘configure_dataset’ function to create a subgraph, and run the model using ‘fit_SCGLUE’. All parameters are set to their default values as in the tutorial. Finally, we obtain the integrated embeddings using ‘glue.encode_data’.

M32: SMILE*[32] *(github version a2e2ca6). The count matrices from the mosaic data combination of RNA and ATAC are used as input. Following (https://github.com/rpmccordlab/SMILE/blob/main/Tutorial/SMILE_data_integration_withReference.ipynb), we use ‘littleSMILE’ and ‘ReferenceSMILE_trainer’ functions to train the model. Upon completion of the training, we utilise the network’s encoder to extract latent embeddings.

M33: StabMap*[33] *(v0.1.8). Following the authors’ vignette (https://marionilab.github.io/StabMap/articles/stabMap_PBMC_Multiome.html), a data list with multi-batch and multi-modal data is fed into the stabMap function using the default setting to do integration, resulting in the integrated embeddings. For imputation purposes, we use the ‘imputeEmbedding’ function to obtain the imputed data.

M34: MultiVI*[34] *(v1.1.2). We use the ‘data.organize_multiome_anndatas’, ‘model.MULTIVI.setup_anndata’, ‘model.MULTIVI’, and ‘train’ functions with the default settings to train the model (https://docs.scvi-tools.org/en/stable/tutorials/notebooks/multimodal/MultiVI_tutorial.html). Finally, we use the ‘get_latent_representation’ function to obtain the integrated embeddings. For imputation purposes, we use ‘get_normalized_expression’ and ‘get_accessibility_estimates’ functions, respectively, to obtain the imputed RNA and ATAC modalities.

M35: Cobolt*[35] *(v1.0.1). We use the 'MultiomicDataset.from_singledata' function to create a MultiomicDataset from the given datasets. Then, we initialise and train the model using the 'Cobolt' (learning_rate = 0.0005) and 'train' functions with other parameters default (https://github.com/epurdom/cobolt). Finally, the integrated embeddings are acquired using the 'get_all_latent' function.

M36: PASTE*[36] *(v1.4.0). The count data with spatial coordinates are used as input. PASTE has two integration approaches (https://github.com/raphael-group/paste). The first is pairwise alignment, where we align pairwise slices using the 'pairwise_align function', followed by the 'stack_slices_pairwise' function to obtain the aligned coordinates. The second approach is centre alignment, for which we utilise the 'center_align' function to train the model and then obtain the aligned coordinates.

M37: SPIRAL*[37] *(v1.0). The count data with spatial coordinates are used as input. Following the authors' instructions (https://github.com/guott15/SPIRAL/blob/main/Demo/run_spiral_DLPFC.ipynb), we use the 'SPIRAL_integration.train', 'CoordAlignment.New_Coord' functions to train the model and then obtain the aligned coordinates.

M38: GPSA*[38] *(v0.8). The count data with spatial coordinates are used as input. Following the authors' instructions (https://github.com/andrewcharlesjones/spatial-alignment), we use the 'VariationalGPSA' function to construct the model. We then perform a forward pass using the forward function and calculate the loss with 'loss_fn'. The network underwent training for 100 epochs as default. Finally, we use the 'forward' function to obtain the aligned coordinates.

M39: PASTE2*[39] *(github version b71ec88). The count data with spatial coordinates are used as input. Following the authors' tutorial (https://github.com/raphael-group/paste2/blob/main/tutorial.ipynb), we align pairwise slices using 'partial_pairwise_align', followed by 'partial_stack_slices_pairwise' to obtain the aligned coordinates.

M40: uniPort*[40] *(v1.2.2). Following the authors' tutorial (https://uniport.readthedocs.io/en/latest/index.html), the count matrices from both RNA and ATAC are used as inputs. Then, normalisation, log-transformation , and batch scale are applied to both modalities. Finally, we use the 'Run' function to train and extract the integrated embeddings from the model.

Evaluation metrics

Dimension reduction

Biological conservation metrics

Graph cell-type local inverse Simpson's index (cLISI) score. The graph cell-type LISI (cLISI) score implemented in the *scib* package[41] assesses the cell-type separation of integrated data. It is calculated by extracting neighbourhood lists from integrated kNN graphs and computing graph distances using shortest path lengths. The graph cLISI scores are rescaled to a range of 0 to 1, quantifying the level of cell-type separation in the integrated data generated by a data integration method.

Dimension reduction scalability metrics

Since generating embeddings from single-cell multimodal omics data is a computationally intensive task and is also a critical component common to most integration methods, we benchmark methods for their scalability in terms of the computational time and memory usage required to complete this task.

Computational time on dimension reduction (CT). We evaluate running time on a server with AMD(R) Ryzen processor CPU (16 cores and 128 Gb total memory) and one RTX3090 graphics processing unit. For benchmarking purposes, we choose some datasets as our foundational datasets to create various benchmarking sets for measuring runtime. To evaluate the impact of the number of cells, we generate benchmarking datasets containing 500, 1,000, 2,000, 3,000, 5,000, 10,000, 20,000, 30,000, 50,000, 80,000, and 100,000 cells each. In cases where the base datasets consist of multiple batches, each batch is configured to have an equal number of cells. To measure the elapsed runtime, we utilise the 'system.time()' function in R and the 'time()' function from the time package in Python, corresponding to methods implemented in R and Python, respectively.

Memory usage on dimension reduction (MU). Using the same benchmarking datasets as those employed for computing computational time, we adopt specific functions to monitor memory usage. For tracking CPU memory, we use the '/usr/bin/time -v command' for R and Python. Meanwhile, GPU memory consumption is monitored using the 'nvidia-smi' tool. To calculate the total memory usage involving both CPU and GPU, we sum the memory readings from both sources.

Batch correction

Batch correction quality metrics

K-nearest neighbour batch effect test (kBET) score. The kBET score quantifies the similarity between the label composition of a k-nearest neighbourhood in an integrated dataset and the expected label composition. It is introduced to measure the rejection rate of tested neighbourhoods in k-nearest neighbour (kNN) graphs to evaluate batch effects. We apply the kBET algorithm implemented in the *scib* package[41] to quantify the batch effect.

Graph integration LISI score (iLISI). Similar to graph cLISI score but calculated for batches in the integrated data, the graph integration LISI (iLISI) score[41] assesses the batch mixing of integrated data. The graph iLISI scores are rescaled to a range of 0 to 1, quantifying the level of batch integration achieved by the data integration methods.

Adjusted Rand Index by batch (ARI_batch). ARI is a metric used to assess the concordance between two partitions of a set of items by considering both the overlaps and the disagreements between the two partitions[42]. Here ARI_batch is used for quantifying the concordance between cells grouped by clusters and by their batch labels in a dataset.

Normalised mutual information by batch (NMI_batch). Similar to ARI, NMI is a metric used to assess the agreement between two partitions of a set of items and ranges between 0 (uncorrelated) and 1 (perfect match).

Average Silhouette width by batch (ASW_batch). The silhouette width quantifies the within-cluster distances of a cell and the between-cluster distances of that cell to the closest cluster[43]. Averaging the silhouette width across all cells gives ASW. Luecken et al.[41] proposed a modified ASW for quantifying batch mixing ('ASW_batch'). We follow this definition and its implementation in the *scib* package[41] for calculating ASW_batch where a value of 1 represents ideal batch mixing and 0 indicates strong batch separation.

Graph connectivity (GC). The GC metric assesses the connectedness of cells with the same cell-type label in the integrated kNN graph. It is a metric introduced by Luecken et al.[41] for quantifying batch effect. We use its implementation in the *scib* package[41] for calculating GC.

Principal component regression (PCR) score. The PCR score is used to assess the impact of data integration on variance, comparing the explained variance before and after integration. We use the metric implemented in *scib* package[41], which ranges from 0 to 1, where 0 indicates no change in variance contribution, and 1 indicates a significant difference in variance contributions before and after integration.

Clustering

Clustering metrics

Adjusted Rand Index by cell type (ARI_cellType). ARI_cellType is used for quantifying the concordance between cells grouped by clusters and by cell type annotation in their original publications. The definition of ARI_cellType is the same as ARI_batch except that the batch clusters/labels are replaced by cell type clusters/labels.

Normalised mutual information by cell type (NMI_cellType). NMI_cellType is used for quantifying the concordance between cells grouped by clusters and by cell type annotation in their original publications. The definition of NMI_cellType is the same as NMI_batch except that the batch clusters/labels are replaced by cell type clusters/labels.

Average silhouette width by cellType (ASW_cellType). As described above, the silhouette width quantifies the within-cluster distances of a cell and the between-cluster distances of that cell to the closest cluster and its average across all cells gives ASW.

Isolated label score of F1 (iF1). An isolated label is defined as a label that appears in the minimum number of batches, which can be extended to labels that appear in fewer than a specified number of batches. This score introduced by Luecken et al.[41] indicates how well the isolated labels are separated from other cell types and can be calculated from the *scib* package[41] for quantifying the quality of clustering results. We average the isolated scores for all clusters and present the final average score.

Isolated label score ASW (iASW). The *scib* package[41] calculates the ASW for the isolated labels in comparison to the non-isolated labels. This score is then scaled to a range between 0 and 1. We average the isolated scores for all clusters and present the final average score.

Classification

Classification metrics

Overall classification accuracy (OCA). Overall classification accuracy is determined by dividing the number of correctly predicted cells by the total number of cells in the dataset. This metric provides an overall measure of a model's predictive performance in correctly classifying cells.

Average classification accuracy (ACA). Average classification accuracy is computed by summing up the accuracies across each cell type and then dividing by the number of cell types in the dataset. This metric is particularly useful in datasets where the number of cells between cell types significantly varies, ensuring that the model's performance is evaluated across all cell types, rather than being influenced by the performance of more abundant cell types.

Specificity (Spec) and sensitivity (Sens). Specificity measures the proportion of actual negatives that are correctly identified. A higher specificity indicates better detection of true negatives, reducing the chance of false positives. Sensitivity is defined as the proportion of true positives correctly identified in the test. It quantifies how effective a model is to identify positive cases among all the truly positive cases. The range for both specificity and sensitivity are from 0 to 1, with values closer to 1 indicating better performance. We use one-versus-all to calculate the specificity and sensitivity for the classification performance of a method for each cell type.

F1-score (F1). F1-score is computed as the harmonic mean of precision and recall. Precision is the ratio of true positives within the predicted positive cases and recall is equal to sensitivity. The F1-score ranges from 0 to 1, where 0 denotes the poorest performance and 1 represents the best performance. We use one-versus-all to calculate the F1-score for the classification performance of a method for each cell type.

Feature selection

Feature selection is frequently used for identifying cell type markers and can be used in various downstream analyses. We select top markers by their ranking and/or feature importance scores with respect to each cell type determined by each feature selection method.

Feature specificity and reproducibility metrics

Marker overlap among different cell types (MO). We identify the top 5, 10, and 20 markers for different cell types and assess the overlaps among these markers via the *intersect* function in R. A smaller overlap suggests the markers selected for each cell type are distinct and specific.

Marker correlation among downsampled data (MC). We randomly subsample cells to various percentages of the original data (100%) to 80%, 50%, 30%, and 10%. We then run the feature selection methods from the targeted integration approaches to derive the feature importance scores for each subsampled dataset and calculate the Pearson's correlation of feature importance scores across different sampling results. A higher correlation indicates higher feature selection reproducibility.

Feature selection downstream analysis metrics

Clustering metrics applied to top features. We take the union of the top 5, 10, and 20 markers from each cell type, as identified from each feature selection method, to conduct clustering analysis. The metrics used in this analysis are the same as those employed in the clustering task, i.e. ARI_cellType, NMI_cellType, ASW_cellType and iF1, iASW.

Classification metrics applied to top features. We take the union of the top 5, 10, and 20 markers from each cell type, as identified from each feature selection method, to conduct classification analysis. The metrics used in this analysis are the same as those employed in the classification task, i.e. OCA, ACA, Spec, Sens and F1.

Imputation

Imputation data structure metrics

Standardized MSE imputation error with ground truth data (sMSE). This metric is calculated by taking the mean squared error (MSE) of the differences between the imputed data and the ground truth data held out from the model training, and then dividing it by the variance of the ground truth. We rescale this metric to the range [0, 1] and take its reciprocal. Thus, a higher score indicates that the imputed data is closer to the ground truth data, reflecting higher accuracy in the imputation process.

Preservation of feature correlation structure (pFCS). As implemented in the Matilda framework[5], we calculate the pairwise correlation of the top 100 highly variable features in the imputed data and ground truth data heldout from the model training. The preservation of gene correlation structure can then be quantified by calculating Pearson's correlation of the pairwise correlations computed from the imputed data and ground truth data, with a higher correlation indicating better imputation performance.

Preservation of differential expression statistics (pDES). We apply the Limma R package (v3.44.3) to calculate differential expression (DE) statistics of features for each cell type and compute the average Pearson's correlation of DE statistics calculated from the imputed data and the ground truth data. A higher correlation indicates better preservation of DE statistics in the imputed data.

Imputation downstream analysis metrics

Clustering metrics applied to imputed data. As illustrated in Fig. 1d, we conduct clustering analysis on the imputed data. The metrics used in this analysis are the same as those employed in the clustering task, i.e. ARI_cellType, NMI_cellType, ASW_cellType and iF1, iASW.

Classification metrics applied to imputed data. As illustrated in Fig. 1d, we conduct classification analysis on the imputed data. The metrics used in this analysis are the same as those employed in the classification task, i.e. OCA, ACA, Spec, Sens and F1.

Spatial registration

Spatial registration quality metrics

Label Transfer Adjusted Rand Index (LTARI). As described in Liu et al.[39], LTARI is a metric used to evaluate the spatial registration performance in preserving cell type and/or spatial region labels between the aligned spots across slices. First, we compute the clustering result for each slice. Then, one slice is selected as the reference, and the others are treated as query

slices. For the query data, LTARI assigns new labels to each spot according to the label of the nearest spot in the reference slice. Finally, we calculate the ARI between the newly assigned spot labels of the query slice and the ground truth labelling, thus assessing the registration performance in maintaining label consistency across slices.

Pairwise Alignment Accuracy (PAA). As implemented in PASTE[36], PAA is calculated by summing the weighted pairs of spots that share the same cluster labels across all slices. Then, we average the PAA for all paired slices and present the final average score. A higher PAA indicates better spatial registration performance.

Spatial Coherence Score (SCS). Following the implementation in PASTE[36], the SCS is calculated by employing O'Neill's spatial entropy to quantify the variation in spatial coherence of cluster labels across different slices. Higher SCS values suggest a patterned label distribution among adjacent spots, while lower values indicate a distribution that is closer to random. The average SCS for all slices is calculated to present the final SCS.

Spatial registration scalability metrics

Spatial registration is another computationally expensive task and the benchmark of method scalability provides useful information about their usability in this task. Given that spatial registration methods do not aim to generate integrated embeddings or graphs, they are not included in previous scalability benchmarks. Consequently, they require the following independent comparison methods to assess their scalability.

Computational time on spatial registration (CT_SR). For benchmarking spatial registration methods, we sample from the spatial dataset to create various datasets with different cell numbers (i.e. 500, 1,000, 2,000, 3,000, 5,000, 10,000, 20,000, 30,000, 50,000, 80,000, and 100,000) for measuring runtime. Each batch is configured to have an equal number of cells. All configurations and functions applied align with those detailed in the dimension reduction metrics section.

Memory usage on spatial registration (MU_SR). Using the same sets of datasets in the above benchmark of computational time for spatial registration methods, we also evaluate and benchmark the memory usage using the same functions previously outlined in the dimension reduction metrics section.

Cross validation for supervised integration methods

For the dimension reduction and clustering tasks in vertical integration, two of the evaluated, Matilda and UnitedNet, are supervised methods and thus require cell type labels, while the rest are unsupervised. To ensure a fair comparison, we employ a 5-fold cross-validation procedure for the supervised methods. Specifically, for vertical integration involving a single data batch, we split the entire dataset into five folds. In each iteration, four folds are combined for training, and the remaining fold is used as the test set. Evaluation metrics are calculated across all five test folds and averaged to produce the final scores, enabling a fair comparison across methods. In addition, UnitedNet can be applied to cross integration that involves multiple data batches and for tasks including dimension reduction, clustering, batch correction, and classification. Similar to the above, each batch is divided into five folds. In each iteration, we select matching folds from all batches to serve as the test set, while the

remaining folds are combined to form the training set. Evaluation metrics are calculated across all five test folds and averaged to produce the final scores.

Union markers selection strategy for MOFA+

In vertical integration for the feature selection task, methods such as scMoMaT and Matilda identify multiple sets of cell type-specific markers for each cell type. In contrast, MOFA+ selects a single set of cell type-invariant markers for all cell types. This limits the fair assessment of feature selection results from MOFA+. To address this issue and ensure a fair comparison, we adjust the way MOFA+ selects its union of markers. Specifically, we determine the total number of MOFA+ markers by multiplying the number of cell types by the number of top markers chosen per cell type in the other methods. For example, if the dataset includes five cell types and scMoMaT and Matilda are each instructed to identify the top five markers per cell type, MOFA+ will select a total of 25 markers (5 cell types × 5 markers per cell type). By doing so, we create a union of MOFA+ markers that corresponds directly to the total number of top markers identified by the other methods, thereby enabling a fair, apples-to-apples comparison.

Implementation of GLUE on simulated datasets

GLUE is a diagonal integration method that integrates RNA (gene) and ATAC (peak) modalities without relying on gene activity scores. Instead, it leverages the gene regulatory relationships between genes and peaks to create a regulatory graph for data integration. However, because simulated datasets lack the accessibility ranges and gene names required to construct such regulatory relationships between genes and peaks, GLUE cannot be applied directly to these datasets. To overcome this challenge, we artificially construct a regulatory graph by linking each RNA feature (gene) to its corresponding ATAC feature (gene activity score). We ensure there are no extraneous links between unrelated features, thus preserving a one-to-one mapping. This adjustment generates the regulatory graph required for GLUE, thereby enabling its application to simulated datasets.

Implementation of Seurat v3/v5 on simulated datasets

Seurat v3 and v5 integrate RNA data with either peaks or gene activity scores from ATAC data. For real datasets, we use peaks for integration. However, for simulated datasets that lack chromatin accessibility ranges, we use gene activity instead. The details for integrating gene activity scores with these two methods are as follows:

Seurat v3. For gene activity score-based integration, we follow the same processing steps as RNA data (NormalizeData, FindVariableFeatures, ScaleData, RunPCA, and RunUMAP), rather than using the peak-based integration workflow (RunTFIDF, FindTopFeatures, RunSVD, and RunUMAP). When using TransferData, we use the 'pca' parameter instead of the 'lsi' parameter for weight reduction for gene activity score-based integration.

Seurat v5. Peak-based integration utilises 'SCT' normalisation for RNA and 'LSI' normalisation for ATAC data. In contrast, gene activity score-based integration applies the same log normalisation and processing steps for both modalities, including NormalizeData, FindVariableFeatures, ScaleData, RunPCA, and RunUMAP. All functions that rely on SCT and LSI-reduced data are replaced with those using log-normalised reduced data.

Reference data setup for Seurat v5

For diagonal integration using Seurat v5, a bridge dataset is required for integrating RNA and ATAC modalities. For datasets with paired modalities (D24–D27, D32, D33, SD7–SD8) but are treated as unpaired for the diagonal integration task, we use the original pairing as the bridge. To also include truly unpaired datasets for evaluation, we try to match the species and tissue types and select D22 as the bridge for D28, D30, and D31, and batch 1 of data source 15 as the bridge for D29.

Classification strategies for methods with original classifiers

For methods that introduced their own classifiers in the original publications, we use those exact implementations to obtain the classification results besides using the MLP classifier. Specifically, Multigrate employs a random forest classifier, while sciCAN and SMILE both rely on a support vector machine (SVM). Several other methods use k-nearest neighbors (kNN) with different values of k. For instance, GLUE uses k=15; Seurat v5, scJoint, and SCALEX each use k=30; and MultiMAP, Concerto, and StabMap each use k=5.

Several methods also include specialised functions within their respective software packages for classification. These include using the TransferData function for Seurat v3, the predict function for sciPENN, the predict_label function for UnitedNet, the infer_result function (from eval_utils) for scBridge, the metrics.label_transfer function for uniPort, the annotate_by_nn function (applied post-integration) for Portal, the propagateLabels function for Conos, and the findNeighbors function for VIPCCA.

Setup of datasets for imputation

In mosaic integration for the imputation task, memory usage presents a significant challenge for large datasets for most methods, where generating imputed data within a reasonable time becomes computationally challenging. To address this, we downsample the features by selecting the top 1,000 highly variable features for RNA and ATAC, while retaining all ADT features. Additionally, for multi-batch datasets, we select two groups comprising one reference and one query, respectively, for evaluation. Detailed descriptions of the datasets are provided in Supplementary Table 1.

Ground truth data preprocessing for imputation metric calculation

Because different methods have distinct input requirements, we apply method-specific preprocessing steps to the ground truth raw data so that each method receives its preferred data format. This approach ensures a fair comparison when calculating sMSE and performing downstream classification. Specifically, UnitedNet, moETM, and totalVI use raw counts. sciPENN applies the ‘preprocess’ function from its package. StabMap performs log-normalisation for both raw RNA and ATAC data, applies CLR normalisation for the raw ADT data, and then scales each modality. scMM normalises the raw data for each modality through division of each element by the library size, then applies scaling using a specified factor 10,000. For MultiVI, we normalise RNA modality using library size normalisation while keeping ATAC as raw counts.

Strategy for large spatial datasets

The spatial dataset D62 consists of two slices each containing 167,780 and 118,752 cells, respectively. The exceptionally large size of the dataset causes out-of-memory issues for all methods. To mitigate this issue, we divide slices one and two into 10 paired folds. We run methods on each paired fold and calculate the evaluation metrics across all 10 folds from the two slices.

Implementation of mapping scores for PAA

The metric PAA measures the weighted pairs of spots that share the same cluster labels across paired slices. For PASTE_pairwise, the 'pairwise_align' function from the PASTE package is used to calculate the mapping scores for each pair of slices. For PASTE_centre, the 'center_align' function from the PASTE package is used to calculate the mapping scores between each slice and the centre slice. For PASTE2, the 'partial_pairwise_align' function from the PASTE2 package is employed to compute the mapping scores. As SPIRAL and GPSA do not provide mapping scores between pairs of spots, we use an alternative procedure from the PASTE method. The mapping for each aligned pair of slices is determined by minimising the Wasserstein distance between spots of the same type. All spots are assigned equal weights, and the transportation cost is calculated based on the Euclidean distances between their aligned coordinates.

Dataset selection and processing for computational and memory usage

Datasets with large feature spaces can impose substantial memory demands on computational methods and frequently lead to out-of-memory errors. To enable an informative assessment of computational and memory efficiency, we selectively apply highly variable feature selection to specific datasets across different integration categories based on their feature dimensionality. For vertical integration, we use datasets D7, D15, and D22. RNA and ATAC features are restricted to 5,000 for D15 and D22, while the original number of features is retained for D7. For diagonal integration, datasets D24 and D37 are used, retaining their original features without further reduction. For mosaic integration, we use datasets D39, D45, D46, and D49. ATAC features are limited to 5,000 for D45, D46, and D49, while D39 keeps its original features. For cross integration, datasets D53, D56, D58, and D59 are employed. Here, ATAC features are limited to 5,000 for D56, D58, and D59. For spatial registration, dataset D61 is used with all features retained in their original form. By tailoring feature dimensions in this manner, we preserve comparability across methods while mitigating the memory constraints associated with high-dimensional datasets in the benchmark.

Computation for overall rank score

To improve the comparability of diverse metrics, we apply min-max normalisation to the values generated from each metric, scaling them to the range [0,1], as follows:

$$y = \frac{x - min(x)}{max(x) - min(x)}$$

where y are the min-max scaled metric values and x are the raw metric values in a single dataset. For the overall rank score of each task, we calculate it as:

$$z = minmax(mean(rank(y)))$$

When summarising multiple datasets, we calculate the grand metric and grand overall score to provide an overview evaluation across datasets. The grand metric values are computed as follows:

$$k = minmax(\frac{\sum_i \; rank(X_i))}{n})$$

where X_i represents the raw metric values for the i-th dataset, and n is the total number of datasets. Similarly, overall grand rank scores are computed as the min-max scaled mean rank of the grand ranks across each metric as follows:

$$p = minmax(mean(rank(k)))$$

Metrics that are not applicable to a given method are excluded from averaging when calculating the (grand) overall rank score.

Evaluation pipelines

Dimension reduction evaluation pipeline

Algorithm 1 provides the pseudocode detailing the steps for evaluating each integration method in this task. Specifically, for each targeted integration method ***m***, we first prepare the list of corresponding single-batch or multi-batch dataset files ***D*** and generate the integrated embeddings which is denoted by $\boldsymbol{embedding_i}$ for each dataset file $\boldsymbol{d_i}$ (Line 5-7). Then, we calculate the 3 evaluation metrics $\boldsymbol{e_j}$, including cLISI score, CT and MU, for each $\boldsymbol{embedding_i}$ to generate the evaluation result list ***O*** and compare across different integration methods (Line 9-12). The list of generated embeddings ***B*** is saved to files for later use (Line 8).

Batch correction evaluation pipeline

Similar to the dimension reduction evaluation, we first derive the batch-corrected integrated embeddings for each targeted method and on the corresponding datasets. Then, we apply the 7 batch correction metrics to all the embeddings for comparison across different methods. The evaluation pipeline for each method follows the steps in **Algorithm 1**, with the only difference that the evaluation function ***EvaluateBatchCorrection(D, E, m)*** is used with the evaluation metrics ***E*** for the 7 batch correction metrics.

Clustering evaluation pipeline

Algorithm 2 details the pseudocode for the steps involved in evaluating each integration method on the clustering task. We first load the pre-saved embeddings from a method ***m*** from the corresponding file paths ***F*** (Line 4-5). For each $\boldsymbol{embedding_i}$, we apply the Leiden clustering to derive the $\boldsymbol{clusters_i}$ (Line 6). Then, we evaluate the clustering results based on each quality metric (Line 7-10). The evaluation output ***O*** is used for our comparative analyses across different methods and datasets.

Classification evaluation pipeline

Algorithm 3 provides the pseudocode for the steps involved in evaluating each integration method. Specifically, for each pre-save $\boldsymbol{embedding_i}$ of the method ***m***, we construct a data structure named ***classification_summary*** for saving the classification evaluation summary for each integration method and dataset (Line 5-7). We first iterate over each data batch of the embeddings as reference (Line 8-9) while using the remaining batches of embeddings as queries (Line 10). Then, for each query, we apply the classification function that calls each specific method to derive the classification results for the current reference data (Line 11-12). The classification results ***predictions*** are then evaluated using each of the metrics in ***E*** (i.e. OCA, ACA, Spec, Sens and F1) and stored in the ***classification_summary*** structure for the final evaluation analysis (Line 13-16).

Feature selection evaluation pipeline

Algorithm 4 provides the pseudocode for the steps involved in the feature selection for each integration method. For each integration method ***m*** on each dataset $\boldsymbol{d_i}$, we first run the method to generate the feature importance scores (Line 6). For evaluating feature specificity and their utility on classification and clustering, we select the top 5, 10 and 20 features for each cell type based on the feature importance scores and calculate the evaluation results based on the corresponding metrics (Line 10-18). Particularly, for classification, ***classification_cross_validation(top_features, E_{cls})*** in Line 14 conducts the 5-fold cross-validation via a simple MLP classifier using the union of selected top features and then assess the performance by using the 5 classification evaluation metrics. For clustering, ***evaluate_clustering_leiden(top_features, E_{clu})*** in Line 16 applies the Leiden clustering pipeline and calculates the 5 clustering evaluation metrics. Finally, for feature reproducibility, we first randomly subsample 10%, 30%, 50%, 80% of cells and run the integration method to produce the corresponding feature importance scores for each subsample (Line 20-24). We then calculate the pairwise Pearson's correlation for each subsample (Line 25). All the evaluation results are saved for comparative analysis (Line 31).

Imputation evaluation pipeline

Algorithm 5 provides the corresponding pseudocode detailing the steps for evaluating each integration method on the imputation task. After loading the prepared dataset (Line 5), we first randomly select one batch as the reference data (Line 7-8) while selecting other batches as query data (Line 9). Then, for each batch in the query data, we exclude one modality to create the query data with missing features (Line 10-11) and keep the excluded data as ground truth for evaluation (Line 12). We run the integration method to impute the missing modality in the query data based on the reference data (Line 13). With the derived imputed data, we then evaluate the quality using the 3 imputation data structure metrics (Line 14-17), the 5 classification metrics (Line 19-22) and the 5 clustering metrics (Line 24-27). Specifically, the ***label_transfer()*** in Line 18 denotes the aforementioned cell classification of the query data using the reference data, whereas the ***leiden_clustering()*** in Line 23 indicates the Leiden clustering pipeline as introduced in the evaluation procedure of previous tasks. All the evaluation results are saved for the comparative analysis.

Spatial registration evaluation pipeline

Algorithm 6 provides the corresponding pseudocode detailing the steps for evaluating each integration method on the spatial registration task. For each loaded spatial dataset $\boldsymbol{data_i}$ with multiple slices, we run the integration method to generate the registered slices ***registered_slices*** in the aligned coordinates (Line 5-7). Then, we apply the Leiden clustering on each registered slice with regard to both gene expression and spatial coordinates (Line 9-12). As is described above, we utilise the label transfer from the reference to the query slice to evaluate the 3 spatial registration quality metrics (Line 14-17). More specifically, the ***evaluate_quality()*** function in Line 15 includes the label transfer procedure for each reference slice and its corresponding query slices. For the evaluation of scalability (Line 20-23), the ***evaluate_scalability()*** function in Line 21 is applied to each scalability metrics to the aforementioned sub-datasets to measure both the runtime and memory. All the evaluation results are saved for our benchmarking analysis.

Data availability

Published data are downloaded and used to compile the 64 datasets described in the section "Datasets and preprocessing" for benchmarking methods in each integration category and for different tasks. The source data information is detailed in **Supplementary Table 4**. Below is a brief summary of each data source.

Data source 1: CITE-seq data capturing RNA and ADT modalities of human peripheral blood mononuclear cell (PBMC) samples[44]. Cell types in the original study are manually annotated based on RNA and protein expression of known markers from each cell cluster. This annotation is further verified by matching to the Azimuth PBMC database for quality validation using the Azimuth tool (http://azimuth.satijalab.org/app/azimuth). We obtain the data under the accession of E-MTAB-10026 from the Array Express database.

Data source 2: CITE-seq data capturing RNA and ADT modalities of human PBMC samples[45]. Cell types in the original study are annotated using cluster specific differential expression and the reference-based scRNA-seq annotation method implemented in SingleR[46] (v1.4.0). We obtain the data under the accession of GSE166489 from the GEO database.

Data source 3: CITE-seq data capturing RNA and ADT modalities of human PBMC samples[10]. Cell types in the original study are manually annotated based on RNA and protein expression of both known markers and those discovered through clustering analyses. We obtain the data under the accession of GSE164378 from the GEO database.

Data source 4: CITE-seq data capturing RNA and ADT modalities of human bone marrow mononuclear cell (BMMC)[47]. Cell types in the original study are annotated first by known markers from each cluster generated by either RNA or ADT modality using the Scanpy platform, and then, manually validated and cross-modality harmonised. We obtain the data under the accession of GSE194122 from the GEO database.

Data source 5: CITE-seq data capturing RNA and ADT modalities of human BMMC samples[23]. Cell types in the original study are annotated via mapping protein expression from the CITE-seq data to a Human Cell Atlas reference dataset and transferring the labels via Seurat v3. We obtain the data under the accession of https://zenodo.org/records/6348128 from the Zenodo database.

Data source 6: CITE-seq data capturing RNA and ADT modalities of human PBMC samples and lung tissues[48]. Cell types in the original study are manually annotated based on cluster-specific expression of markers within the RNA and/or ADT modality and further aided by cell-by-cell annotation from the SingleR (v1.4.0) using the 'Monaco reference' from the celldex (v1.0.0) made from bulk RNA-seq data of sorted immune cell populations. We obtain the data from https://archive.softwareheritage.org/browse/revision/1c7fcabb18a1971dc4d6e29bc3ed4f6f36b2361f/.

Data source 7: SHARE-seq data capturing RNA and ATAC modalities of mouse skin samples[49]. Cell types in the original study are manually assigned to clusters on the basis of marker genes, TF motifs, and chromatin accessibility peaks. We obtain the data under the accession of GSE140203 from the GEO database.

Data source 8: SNARE-seq data capturing RNA and ATAC modalities of mouse brain samples[50]. Cell types in the original study are manually annotated on the basis of known markers for the cerebral cortex and gene expression patterns from DropViz (http://dropviz.org/). We obtain the data under the accession of GSE126074 from the GEO database.

Data source 9: 10x multiome data capturing RNA and ATAC modalities of human BMMC samples[47]. Cell types in the original study are first determined by cluster-related markers from RNA and ATAC modalities independently using the Scanpy platform. Specifically, the count matrix of open chromatin data is first converted to a gene activity matrix and is then used for the marker-based cell type annotation. Next, modality-specific cell types are manually harmonised. We obtain the data under the accession of GSE194122 from the GEO database.

Data source 10: 10x multiome data capturing RNA and ATAC modalities of mouse embryo samples[51]. Cell types in the original study are annotated by mapping the RNA expression profiles to a reference atlas from the same developmental stages. Specifically, the query and atlas cells are first integrated within a joint PCA space. Then, each query cell is assigned with a cell type based on majority voting over the cell types from its 25 nearest atlas neighbours. We obtain the data under the accession of GSE205117 from the GEO database.

Data source 11: 10x multiome data capturing RNA and ATAC modalities of human PBMC samples. Cell type annotations in the original study are first performed by 10x Genomics and then further validated manually by inspecting markers from an annotated single-cell RNA atlas. We obtain the validated data from https://zenodo.org/records/6348128.

Data source 12: 10x multiome data capturing RNA and ATAC modalities of human neocortex samples[52]. Cell types in the original study are manually annotated on the basis of marker genes for each cluster. We obtain the data under the accession of GSE204684 from the GEO database.

Data source 13: ASAP-seq data capturing ATAC and ADT modalities of human PBMC samples[53]. Cell types in the original study are manually annotated based on the marker gene expression in each cluster in the RNA and ADT modalities. We obtain the data under the accession of GSE156478 from the GEO database.

Data source 14: DOGMA-seq data capturing RNA, ATAC, and ADT modalities of human PBMC samples[53]. Cell types in the original study are annotated by projecting the three-modality WNN (3WNN) clusters derived from Seurat to Azimuth PBMC reference data. We obtain the data under the accession of GSE156478 from the GEO database.

Data source 15: TEA-seq data capturing RNA, ATAC, and ADT modalities of human PBMC samples[54]. Cell types in the original study are derived via label transfer from a multimodal reference atlas for PBMCs using Seurat. We obtain the data under the accession of GSE158013 from the GEO database.

Data source 16: Visium spatial data capturing RNA and spatial coordinates of mouse squamous cell carcinoma[55]. Spot labels in the original study are generated based on independent component analysis. Data are obtained from https://zenodo.org/records/6334774

Data source 17: Visium spatial data capturing RNA and spatial coordinates of human dorsolateral prefrontal cortex samples[56]. Spot labels in the original study are manually annotated based on cytoarchitecture visualised by spatialLIBD tool and selected gene markers. Data are obtained from https://github.com/raphael-group/paste_reproducibility/tree/main/data/DLPFC.

Data source 18: Xenium spatial data capturing RNA and spatial coordinates of human breast cancer tumour. Cell types in the original study are manually annotated based on differential gene expression from each cluster[57]. Data are obtained from https://www.10xgenomics.com/products/xenium-in-situ/preview-dataset-human-breast

Data source 19: Stereo-seq spatial data capturing RNA and spatial coordinates of drosophila embryos[58]. Tissue types at bin level in the original study are manually annotated based on marker genes for each cluster. The clustering is performed using both transcriptomic information and spatial information (via Squidpy[59]). Data are obtained from https://db.cngb.org/stomics/flysta3d/

Data source 20: MERFISH spatial data capturing RNA and spatial coordinates of mouse brain[60]. Cell types of the original study are manually annotated based on the comparison of marker genes and/or spatial locations of clusters, which are obtained from the multi-level clustering of integrated MERFISH and snRNA-seq data, with previously annotated datasets. Data are obtained from https://cellxgene.cziscience.com/collections/31937775-0602-4e52-a799-b6acdd2bac2e

Data source 21: Spatial ATAC-RNA seq data capturing RNA, ATAC and spatial coordinates of mouse embryo (E13) and mouse postnatal (P22) brain[61]. In the original study, cell types in spatial-RNA are transferred from scRNA-seq using Seurat while cell types in spatial-ATAC are transferred from scATAC-seq data using Seurat and Signac. We obtain the data under the accession of GSE205055 from the GEO database.

Supplementary algorithms

Algorithm 1: Pseudocode for Evaluating Dimension Reduction

```
Define
    inputs : list of dataset paths D; list of evaluation metrics E;
             integration method m;
    output: list of integrated embeddings B; list of evaluation results
            O;
EvaluateDimensionReduction (D, E, m)
    B ← list();
    O ← list();
    for data path d_i ∈ D do
        data_i ← load_multimodal_data(d_i);
        embedding_i ← generate_embedding(m, data_i);
        append(B, embedding_i);
        for evaluation metrics e_j ∈ E do
            result_ij ← evaluate(embedding_i, e_j);
            append(O, result_ij);
        end
    end
return B, O;
```

Algorithm 2: Pseudocode for Evaluating Clustering

```
Define
    inputs : list of evaluation metrics E; list of embeddings files F
             generated via integration method m;
    output: list of evaluation results O;
EvaluateClustering (F, E)
    O ← list();
    for embedding path f_i ∈ F do
        embedding_i ← load_integrated_embedding(f_i);
        clusters_i ← leiden_clustering(embedding_i);
        for evaluation metrics e_j ∈ E do
            result_ij ← evaluate(clusters_i, embedding_i, e_j);
            append(O, result_ij);
        end
    end
return O;
```

Algorithm 3: Pseudocode for Evaluating Classification

```
Define
    inputs : list of evaluation metrics E; list of embeddings files F generated via
             integration method m;
    output: list of evaluation results O;
EvaluateClassification (F, E, m)
    O ← list();
    for embedding path f_i ∈ F do
        embedding_i ← load_integrated_embedding(f_i);
        number_of_batches ← get_number_of_batches(embedding_i);
        classification_summary ← initialize_classification_summary();
        foreach batch_id in 1,2,..., number_of_batches do
            reference ← select_batch_as_reference(embedding_i, batch_id);
            queries ← select_batches_as_queries(embedding_i, batch_id);
            foreach query in queries do
                predictions ← classify_cells(m, reference, query);
                for evaluation metrics e_j ∈ E do
                    evaluation_score ← evaluate_classification(predictions, e_j);
                    update_summary(classification_summary, evaluation_score);
                end
            end
        end
        append(O, classification_summary);
    end
return O;
```

Algorithm 4: Pseudocode for Evaluating Feature Selection

```
Define
    inputs : list of dataset paths D; integration method m; feature specificity evaluation
             metrics e_s; feature reproducibility evaluation metrics e_r, list of classification
             evaluation metrics E_cls, list of clustering evaluation metrics E_clu;
    output: list of feature specificity evaluation results O_s, feature reproducibility
            evaluation results O_r, classification evaluation results O_cls, clustering
            evaluation results O_clu;
EvaluateFeatureSelection (D, e_s, e_r, E_cls, E_clu, m)
    O_s, O_r, O_cls, O_clu ← list();
    for data path d_i ∈ D do
        data_i ← load_multimodal_data(d_i);
        importance_score ← apply_feature_selection(data_i, m);
        specificity_results ← list();
        classification_results ← list();
        clustering_results ← list();
        for top_k ∈ {5, 10, 20} do
            top_features ← select_top_features(importance_score, top_k);
            specificity ← evaluate_specificity(top_features, e_s);
            append(specificity_results, specificity);
            classification ← classification_cross_validation(top_features, E_cls);
            append(classification_results, classification);
            clustering ← evaluate_clustering_leiden(top_features, E_clu);
            append(clustering_results, clustering);
        end
        importance_score_subs ← list();
        for portion ∈ {0.1, 0.3, 0.5, 0.8} do
            subset_data ← apply_random_subset(data_i, portion);
            importance_score_for_sub ← apply_feature_selection(subset_data , m);
            append(importance_score_subs, importance_score_for_sub);
        end
        reproducibility_results ← evaluate_reproducibility(importance_score_subs, e_r);
        append(O_s, specificity_results);
        append(O_r, reproducibility_results);
        append(O_cls, classification_results);
        append(O_clu, clustering_results);
    end
return O_s, O_r, O_cls, O_clu;
```

Algorithm 5: Pseudocode for Evaluating Imputation

```
Define
    inputs : list of dataset paths D; integration method m; list of imputation data structure
             evaluation metrics E_imp, list of classification evaluation metrics E_cls, list of clustering
             evaluation metrics E_clu;
    output: list of evaluation results O
EvaluateImputation (D, E_imp, E_cls, E_clu, m)
    O ← list();
    for data path d_i ∈ D do
        data_i ← load_multimodal_data(d_i);

        evaluation_for_dataset ← list();
        batch_id ← select_random_batch(data_i);
        reference ← get_batch_as_reference(data_i, batch_id);
        queries ← get_batches_as_queries(data_i, batch_id);
        foreach query in queries do
            query_with_missing_data ← create_query(query);
            missing_data_groud_truth ← create_query_ground_truth(query);
            imputed_data ← impute(reference, query_with_missing_data, m);
            for evaluation metrics e_j ∈ E_imp do
                evaluation_result ← evaluate_imputation(imputed_data,
                 missing_data_groud_truth, e_j);
                append(evaluation_for_dataset, evaluation_result);
            end

            classifications ← label_transfer(imputed_data, missing_data_groud_truth);
            for evaluation metrics e_k ∈ E_cls do
                evaluation_result ← evaluate_classification(classifications, e_k);
                append(evaluation_for_dataset, evaluation_result);
            end

            clusters ← leiden_clustering(imputed_data);
            for evaluation metrics e_m ∈ E_clu do
                evaluation_result ← evaluate_clustering(clusters, e_m);
                append(evaluation_for_dataset, evaluation_result);
            end
        end
        append(O, evaluation_for_dataset);
    end
return O;
```

Algorithm 6: Pseudocode for Evaluating Spatial Registration

```
Define
    inputs : list of spatial dataset paths D; spatial integration method m; list of spatial registration
             quality metrics E_q; list of spatial registration scalability metrics E_s
    output: list of evaluation results O
EvaluateSpatialRegistration (D, E_q, E_s, m)
    O ← list();
    evaluation_for_dataset ← list();
    for data path d_i ∈ D do
        data_i ← load_spatial_data(d_i);
        registered_slices ← perform_spatial_registration(data_i, m);

        cluster_labels ← list();
        foreach slice_rna, slice_coordinates in registered_slices do
            cluster_labels_for_slice ← clustering(slice_rna, slice_coordinates);
            append(cluster_labels, cluster_labels_for_slice);
        end

        quality_evaluation_results ← list();
        for evaluation metrics e_j ∈ E_q do
            evaluation_result ← evaluate_quality(data_i, registered_slices, cluster_labels, e_j);
            append(quality_evaluation_results, evaluation_result);
        end
        append(evaluation_for_dataset, quality_evaluation_results);

        scalability_evaluatio_results ← list();
        for evaluation metrics e_k ∈ E_s do
            evaluation_result ← evaluate_scalability(data_i, registered_slices, cluster_labels, e_k);
            append(scalability_evaluation_results, evaluation_result);
        end
        append(evaluation_for_dataset, scalability_evaluation_results);

        append(O, evaluation_for_dataset);
    end
return O;
```

Supplementary figures

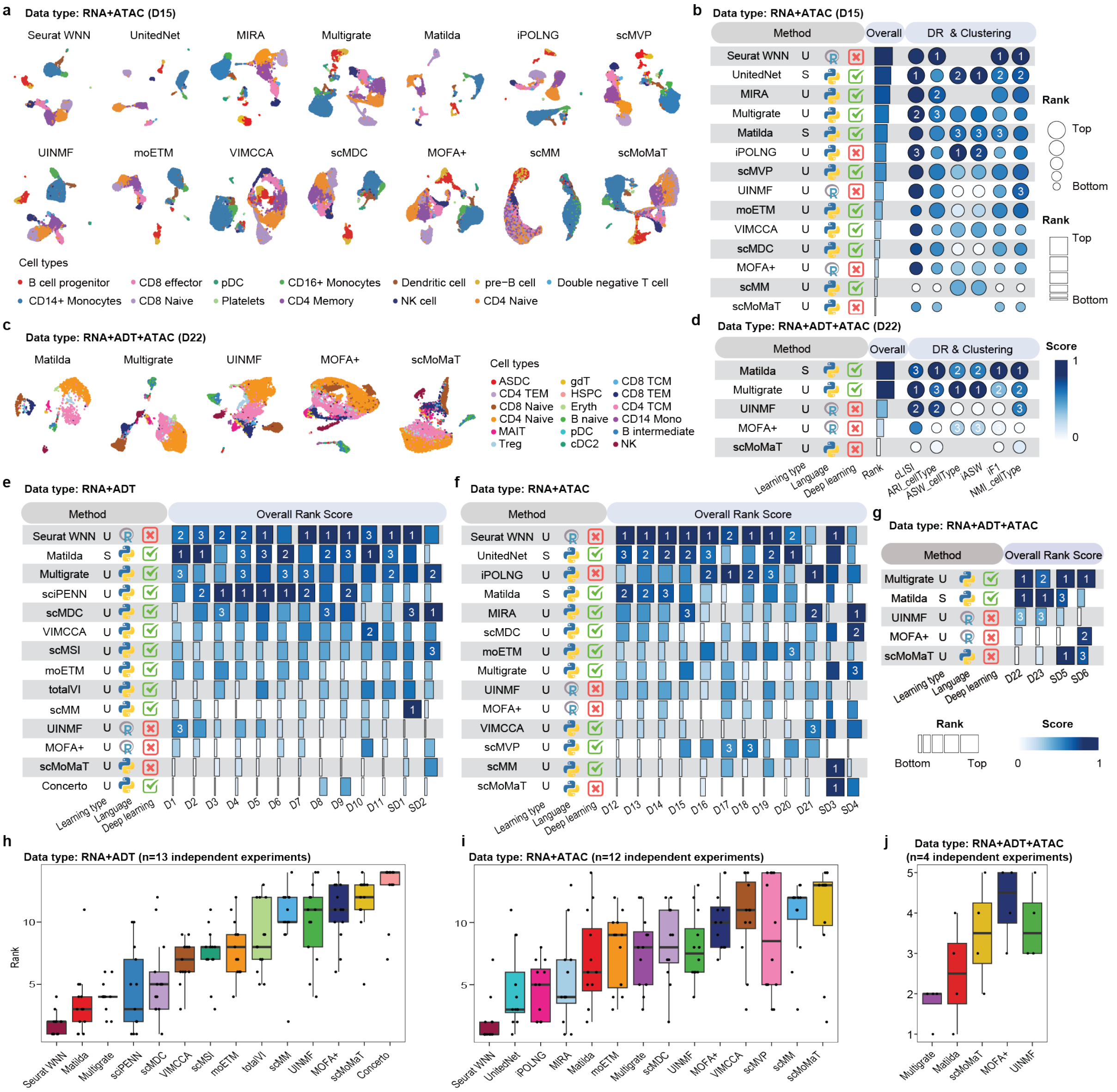

Sup Fig. 1: Extended benchmark results of vertical integration methods for dimension reduction and clustering. a, UMAP visualisation of vertical integration methods applied to a representative RNA+ATAC dataset (D15), and **b**, Method performance on D15. **c,** UMAP visualisation of integration methods applied to a representative RNA+ADT+ATAC dataset (D22), and **d**, Method performance on D22. Summary of overall rank scores for vertical integration applied to **e**, Each of all RNA+ADT datasets; **f**, Each of all RNA+ATAC datasets; and **g**, Each of all RNA+ADT+ATAC datasets. Box plots showing the distribution of method ranks across **h**, All RNA+ADT datasets; **i**, All RNA+ATAC datasets; and **j**, All RNA+ADT+ATAC datasets. In the box plots, the centre lines indicate the median, boxes indicate the interquartile range, and whiskers indicate 1.5× interquartile range. Each dot corresponds to the rank for an individual dataset.

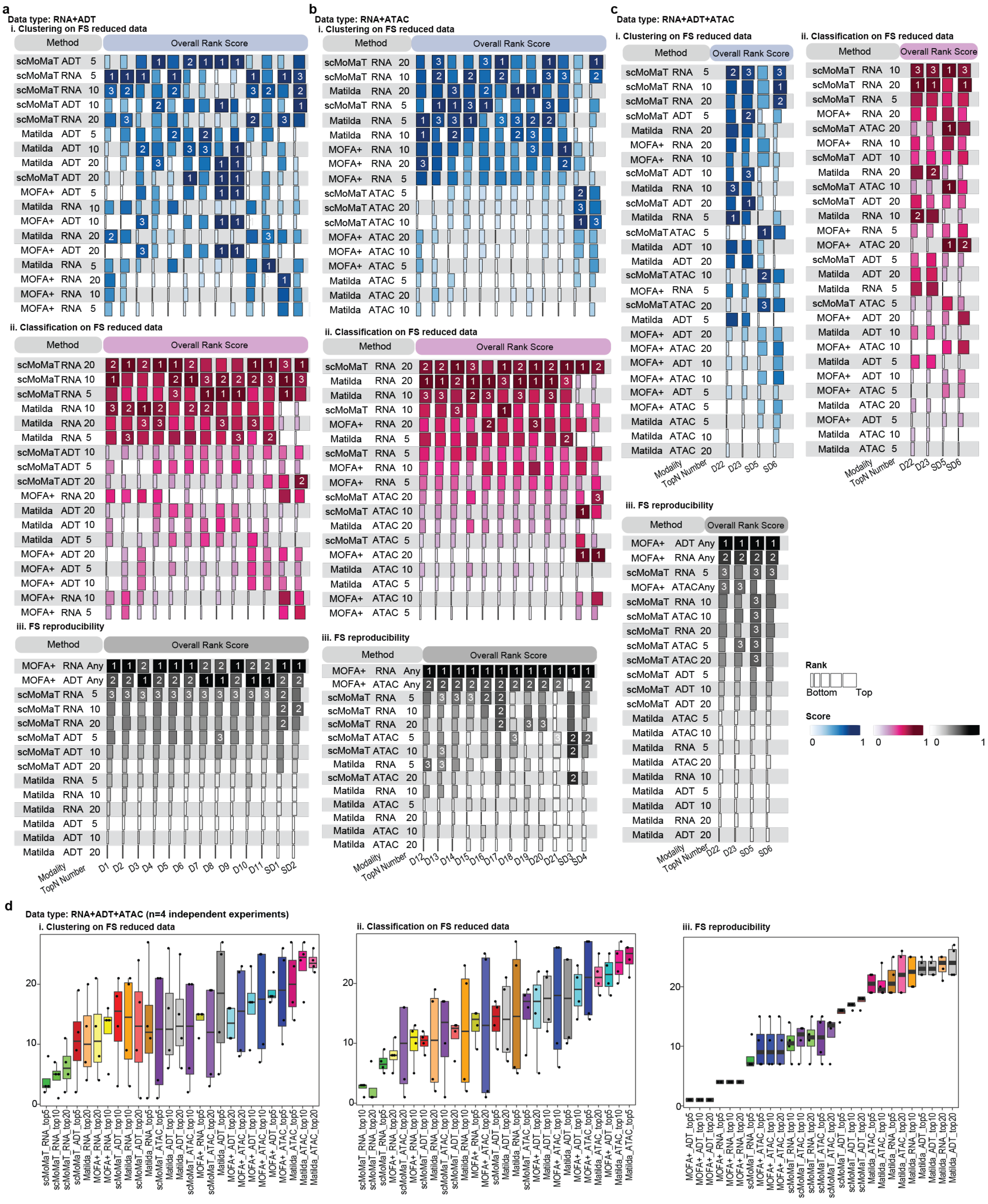

Sup Fig. 2: Extended benchmark results of vertical integration methods for feature selection. Summary of overall rank scores for vertical integration applied to feature selection from **a**, Each of all RNA+ADT datasets; **b**, Each of all RNA+ATAC datasets; and **c**, Each of all RNA+ADT+ATAC datasets, across the evaluation categories of i) clustering, ii) classification, and iii) reproducibility. Box plots showing the distribution of method ranks across **d**, All RNA+ADT+ATAC datasets. In the box plots, the centre lines indicate the median, boxes indicate the interquartile range, and whiskers indicate 1.5× interquartile range. Each dot corresponds to the rank for an individual dataset.

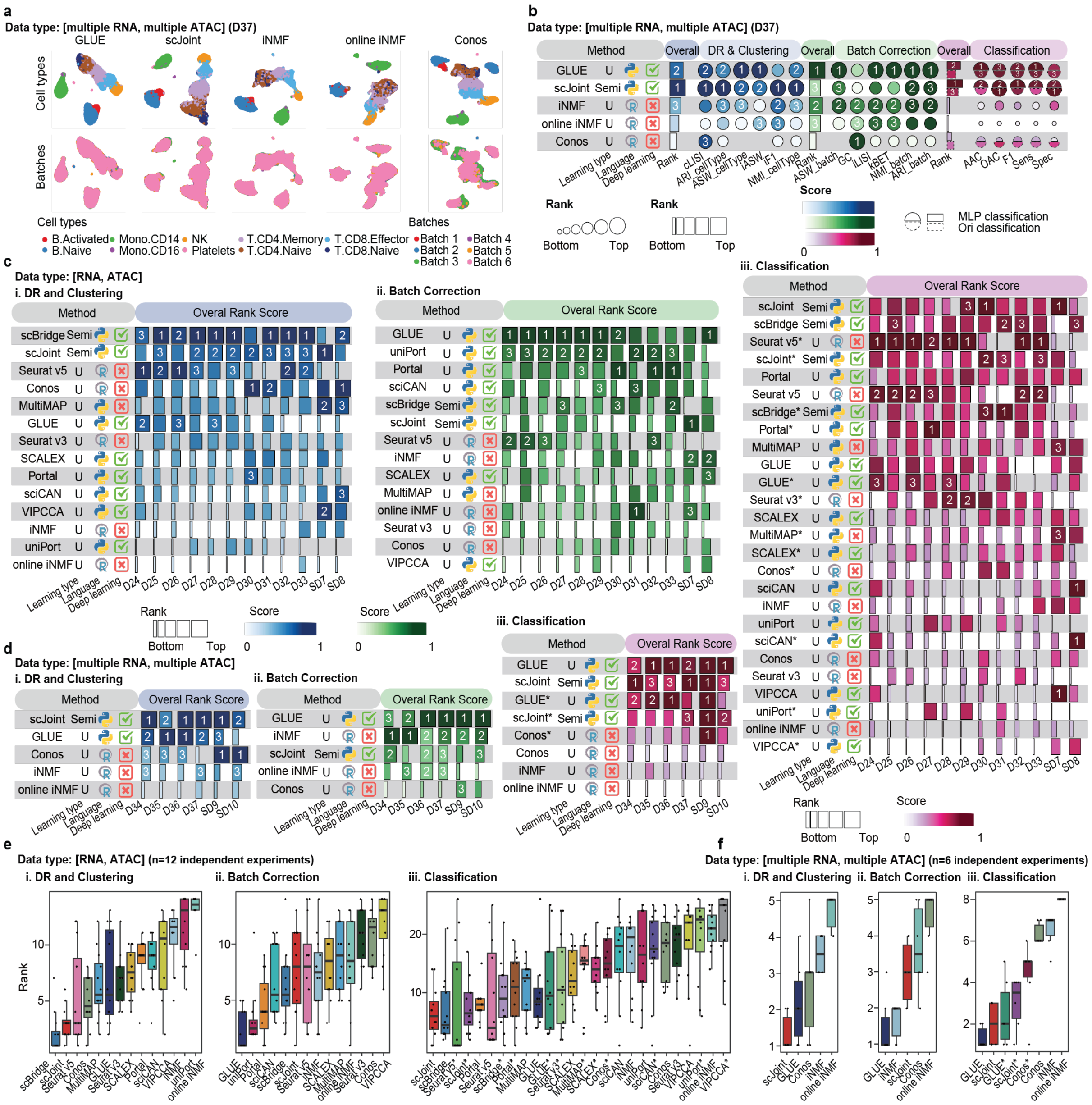

Sup Fig. 3: Extended benchmark results of diagonal integration methods. a, UMAP visualisation of diagonal integration methods applied to a representative dataset of D37 with multiple batches of RNA data and multiple batches of ATAC data, and **b**, Method performance on D37. Summary of overall rank scores for diagonal integration applied to **c**, Each of all datasets with a single batch of RNA data and a single batch of ATAC data; and **d**, Each of all datasets with multiple batches of RNA data and multiple batches of ATAC data, across the evaluation categories of i) dimension reduction and clustering, ii) batch correction, and iii) classification. For the classification summary, the method without * represents the MLP classifier implemented in this study, while the method with * represents the classifier proposed in the original papers. Box plots showing the distribution of method ranks across **e**, All datasets with a single batch of RNA data and a single batch of ATAC data; and **f**, All datasets with multiple batches of RNA data and multiple batches of ATAC data. In the box plots, the centre lines indicate the median, boxes indicate the interquartile range, and whiskers indicate 1.5× interquartile range. Each dot corresponds to the rank for an individual dataset.

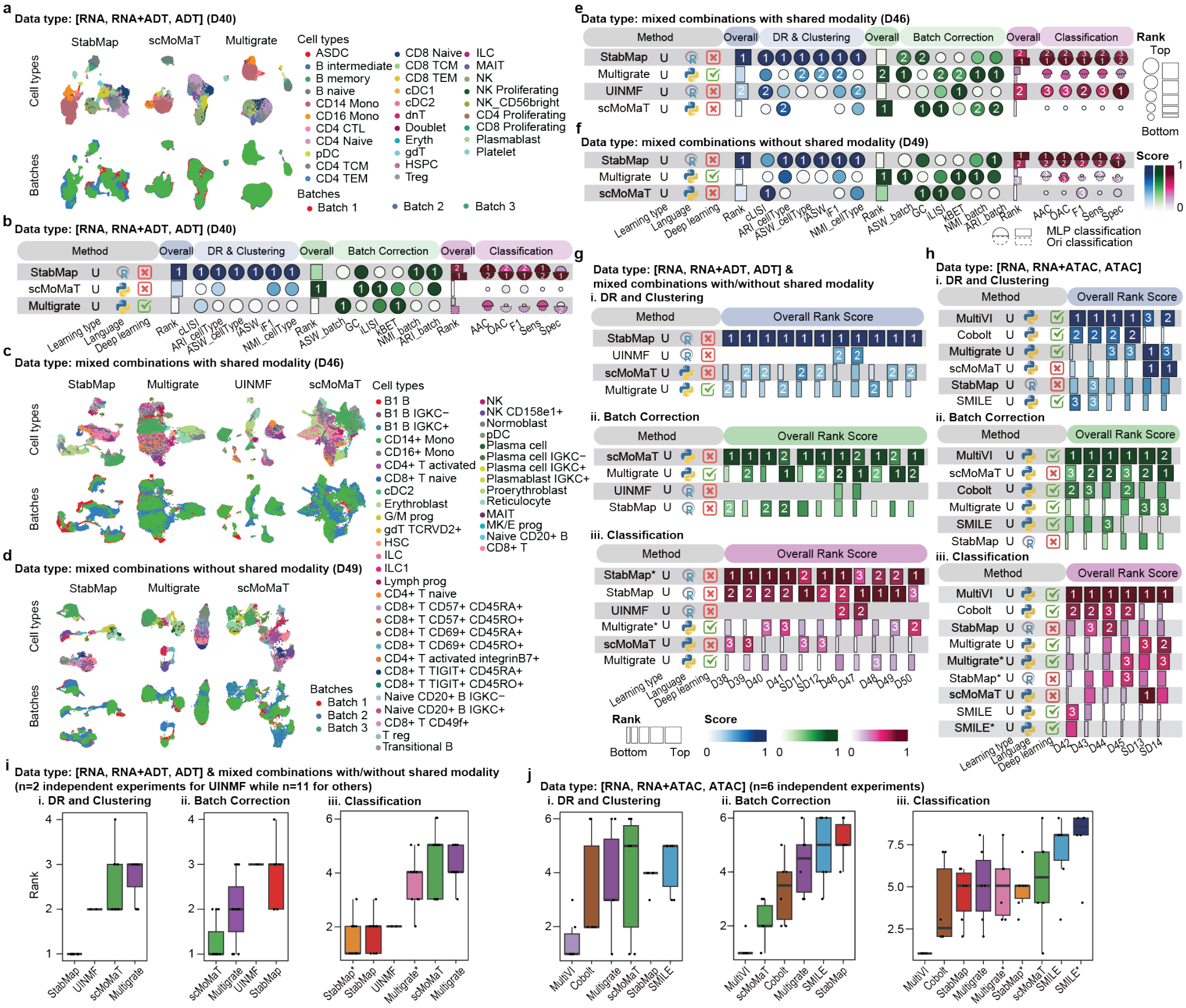

Sup Fig. 4: Extended benchmark results of mosaic integration methods. a, UMAP visualisation of methods applied to a representative dataset of D40 with data batches of RNA, paired RNA+ADT, and ADT. **b**, Method performance on the dataset D40. UMAP visualisation of methods applied to a representative dataset of **c**, D46 with mixed batch combinations and shared modality across batches; and **d**, D49 with mixed batch combinations and without shared modality across batches. Method performance on **e**, D46 and **f**, D49. **g**, Method performance on all datasets with batches of RNA, paired RNA+ADT, and ADT and mixed batch combinations. **h**, Method performance on all datasets with batches of RNA, paired RNA+ATAC, and ATAC. Box plots showing the distribution of method ranks across **i**, All datasets with batches of RNA, paired RNA+ADT, and ADT and mixed batch combinations; and **j**, All datasets with batches of RNA, paired RNA+ATAC, and ATAC. In the box plots, the centre lines indicate the median, boxes indicate the interquartile range, and whiskers indicate 1.5× interquartile range. Each dot corresponds to the rank for an individual dataset.

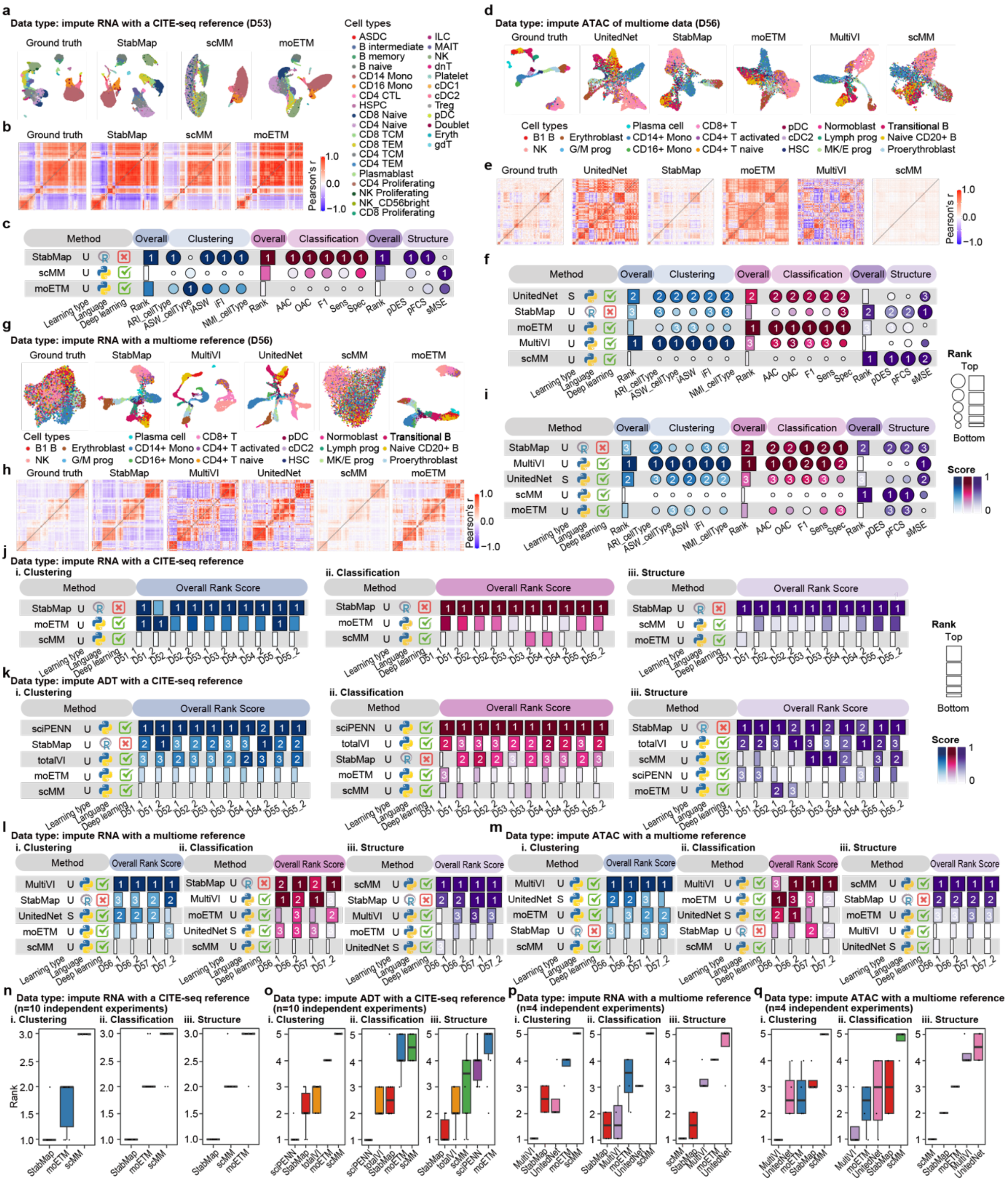

Sup Fig. 5: Extended benchmark on imputation for mosaic data analysis. a, UMAP of D53 for ground truth and imputed RNA. **b**, Pairwise correlation heatmap of top-100 HVGs in ground truth and imputed RNA of D53. **c**, Method performance on D53 for RNA imputation. **d**, UMAP of D56 for ground truth and imputed ATAC. **e**, Pairwise correlation heatmap of top-100 highly variable peaks in ground truth and imputed ATAC of D56. **f**, Method performance on D56 for ATAC imputation. **g**, UMAP of D56 for ground truth and imputed RNA. **h**, Pairwise heatmap of top-100 HVGs in ground truth and imputed RNA of D56. **i**, Method performance on D56 for RNA imputation. Performance summary of overall rank scores for methods applied to all applicable datasets for imputing **j**, RNA in CITE-seq; **k**, ADT in CITE-seq; **l**, RNA in multiome; and **m**, ATAC in multiome. Box plots of method ranks across all applicable datasets for imputing **n**, RNA in CITE-seq; **o**, ADT in CITE-seq; **p**, RNA in multiome; and **q**, ATAC in multiome. In the box plots, the centre lines indicate the median, boxes indicate the interquartile range, and whiskers indicate 1.5× interquartile range. Each dot corresponds to the rank for an individual dataset.

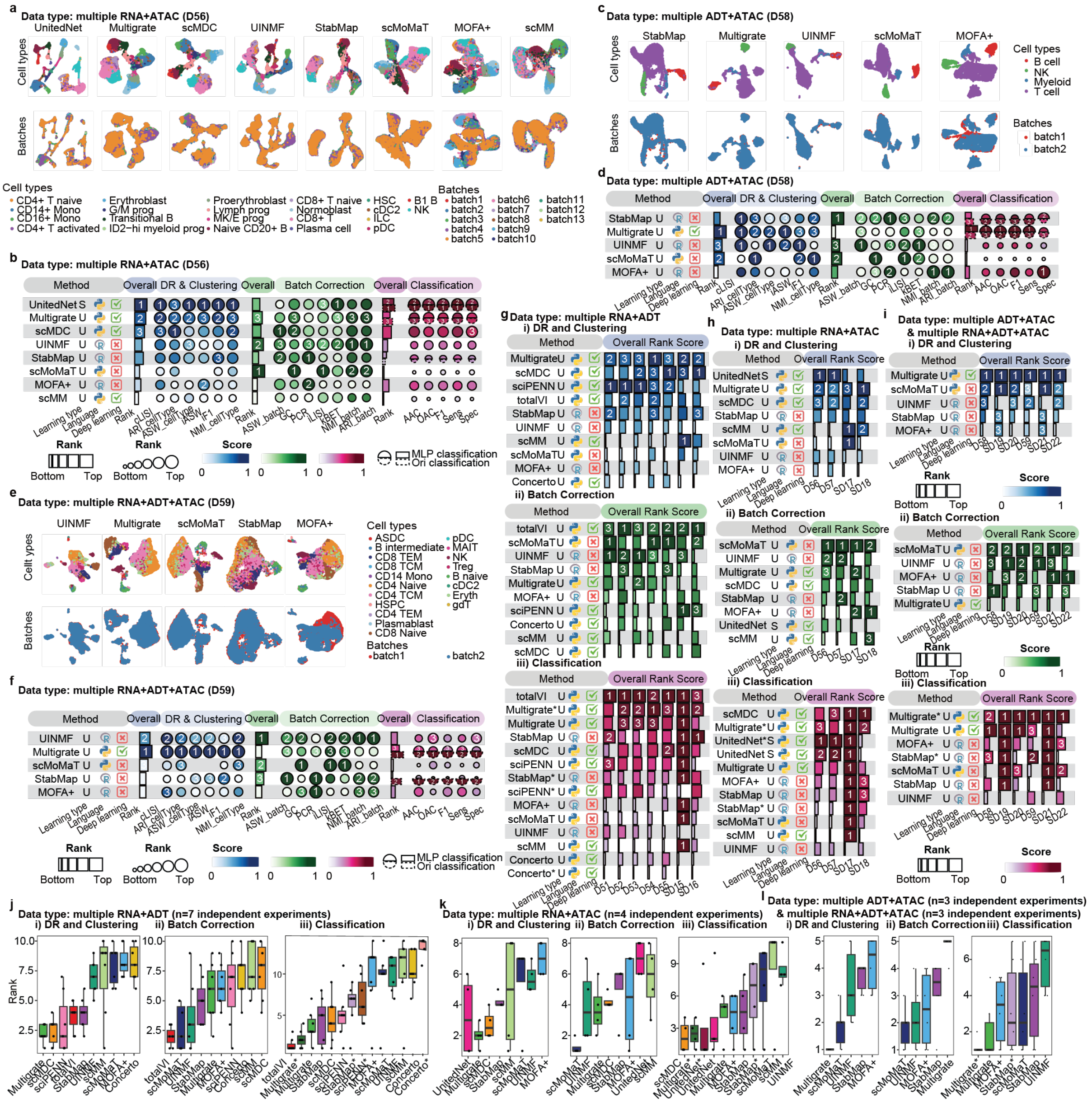

Sup Fig. 6: Extended benchmark results of cross integration methods. a, UMAP visualisation of cross integration methods applied to a representative data of D56 with paired RNA+ATAC data across multiple batches. **b**, Method performance on the dataset D56. **c**, UMAP visualisation of cross integration methods applied to a representative data of D58 with paired ADT+ATAC across multiple batches. **d**, Method performance on the dataset D58. **e**, UMAP visualisation of cross integration methods applied to a representative data of D59 with paired RNA+ADT+ATAC data across multiple batches. **f**, Method performance on the dataset D59. Performance summary of overall rank scores for cross integration applied to **g**, All datasets with RNA+ADT across multiple batches; **h**, All datasets with RNA+ATAC across multiple batches; **i**, All datasets with ADT+ATAC and RNA+ADT+ATAC across multiple batches, evaluated across the categories of i) dimension reduction and clustering, ii) batch correction, and iii) classification. Box plots showing the distribution of method ranks across **j**, All datasets with RNA+ADT across multiple batches; **k**, All datasets with RNA+ATAC across multiple batches; **l**, All datasets with ADT+ATAC and RNA+ADT+ATAC across multiple batches. In the box plots, the centre lines indicate the median, boxes indicate the interquartile range, and whiskers indicate 1.5× interquartile range. Each dot corresponds to the rank for an individual dataset.

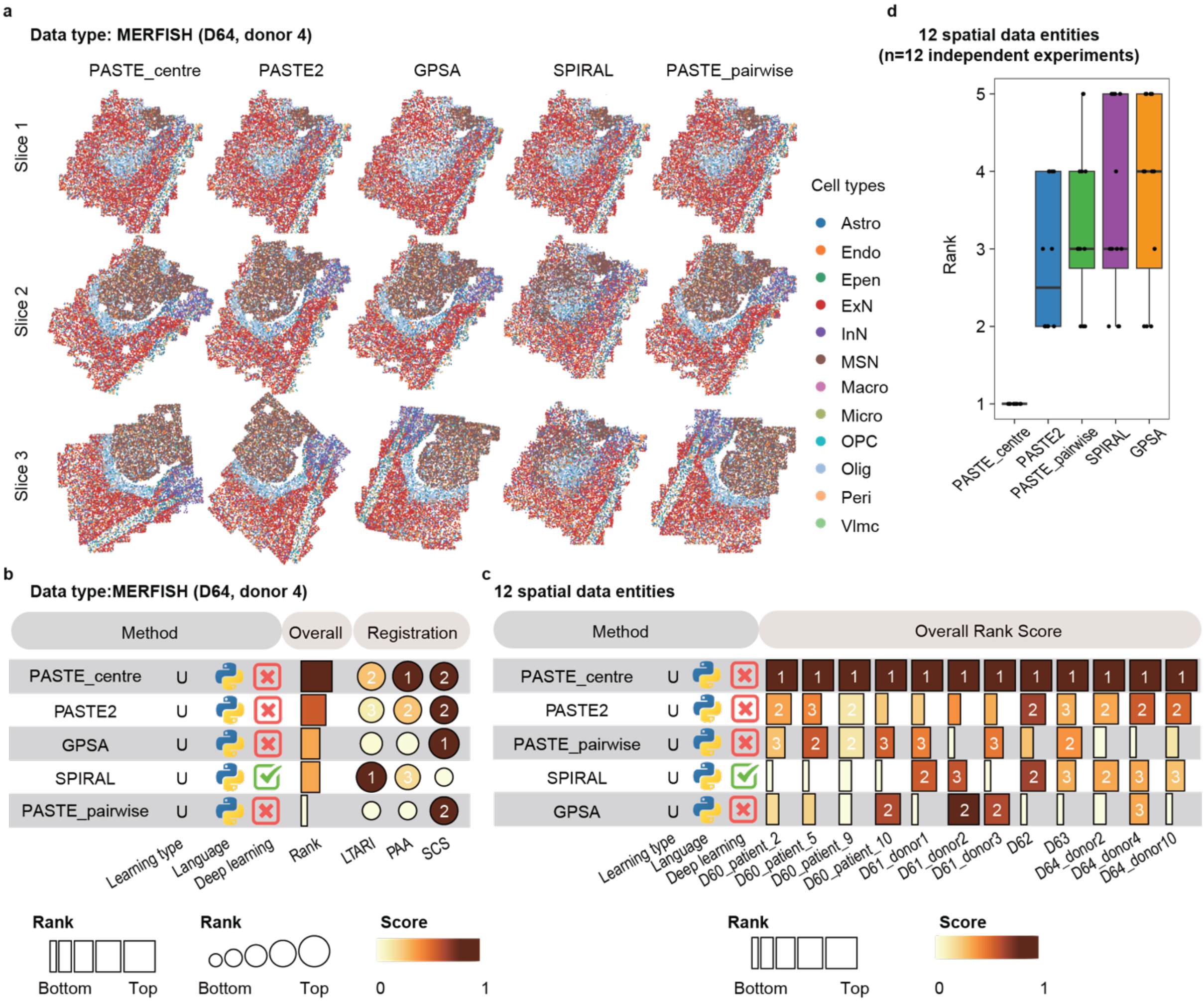

Sup Fig. 7: Extended benchmark results of spatial registration methods. a, Visualisation of cell type spatial arrangements from dataset D64 (donor 4). Each row corresponds to the spatial transcriptomics profile of a tissue slice, and each column corresponds to the spatial registration results from a method across slices. **b**, Quantification of method performance on the dataset D64 (donor 4). **c**, Performance summary of spatial registration methods by overall rank scores for each spatial dataset and patient/donor. **d**, Box plots showing the distribution of method ranks across all datasets. In the box plots, the centre lines indicate the median, boxes indicate the interquartile range, and whiskers indicate 1.5× interquartile range. Each dot corresponds to the rank for an individual dataset.

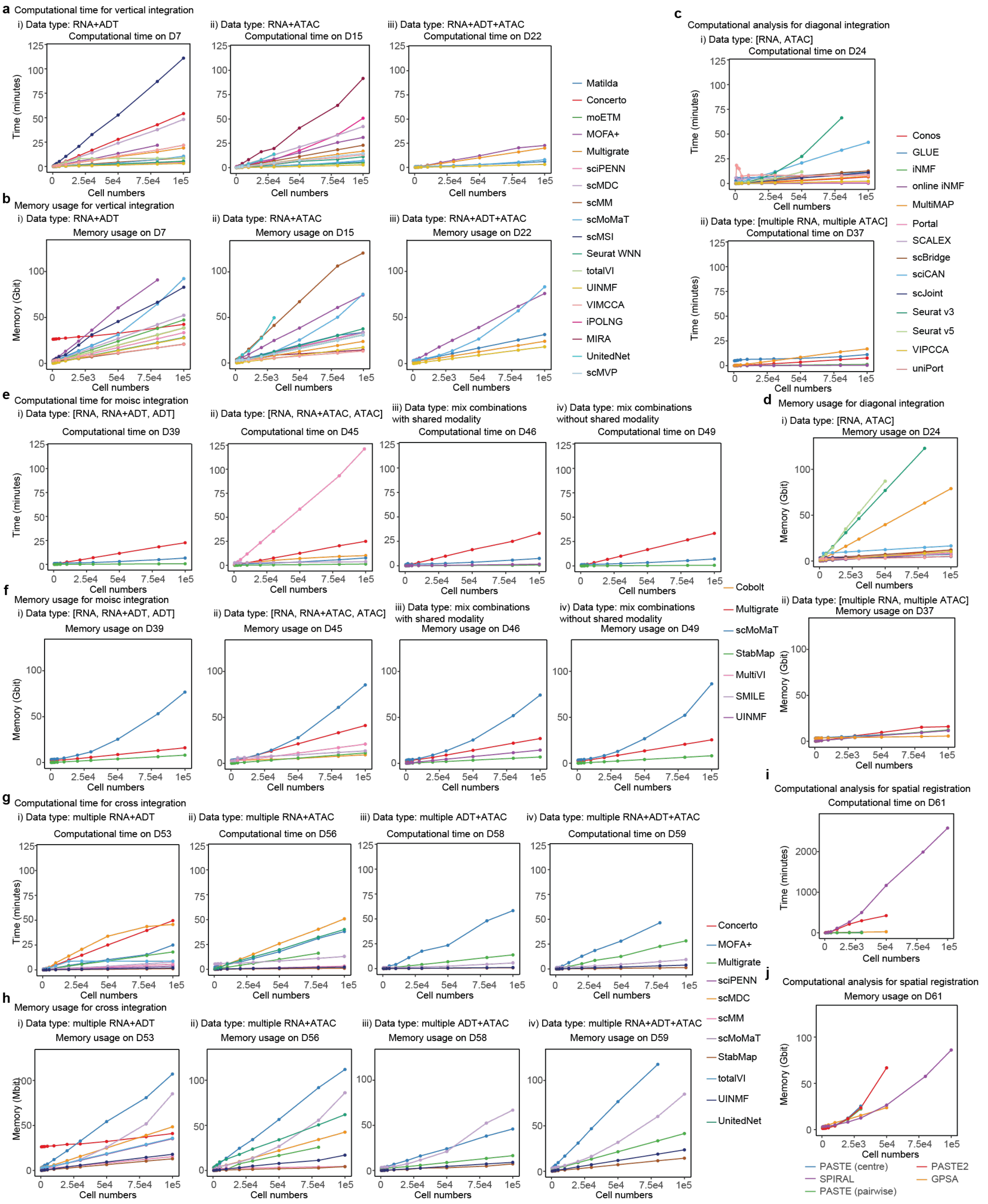

Sup Fig. 8: Summary of methods on computational time and peak memory usage. **a**, Computational time for vertical integration. **b**, Memory usage for vertical integration. **c**, Computational time for diagonal integration. **d**, Memory usage for diagonal integration. **e**, Computational time for mosaic integration. **f**, Memory usage for mosaic integration. **g**, Computational time for cross integration. **h**, Memory usage for cross integration. **i**, Computational time for spatial registration. **j**, Memory usage for spatial registration.

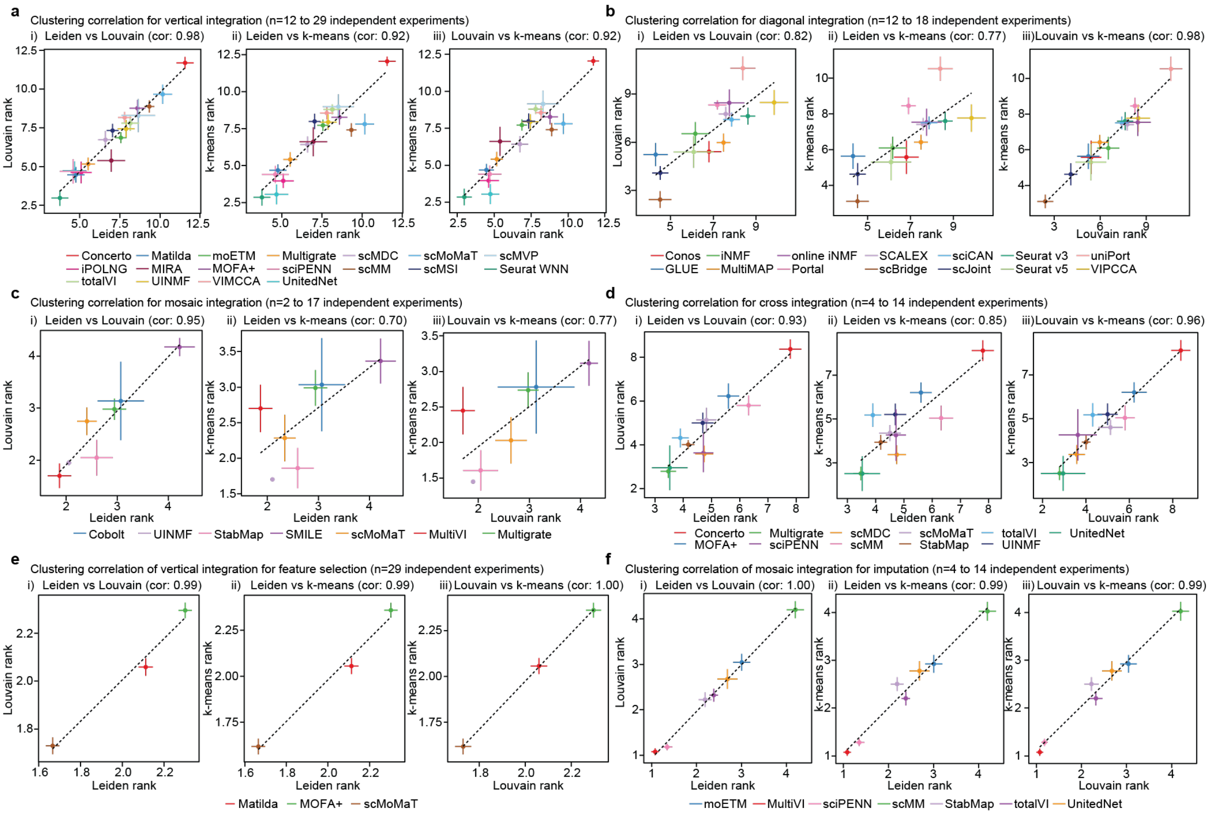

Sup Fig. 9: Impact of clustering algorithms on method evaluation. Pairwise correlation of method ranks from using k-means, Leiden, and Louvain clustering algorithms for evaluating clustering results generated from **a**, Vertical integration methods, **b**, Diagonal integration methods, **c**, Mosaic integration methods, and, **d**, Cross integration methods; **e**, Feature selection results from vertical integration methods; and **f**, Imputation results from mosaic integration methods. Error bars represent the rank variability across all applicable datasets in each evaluation.

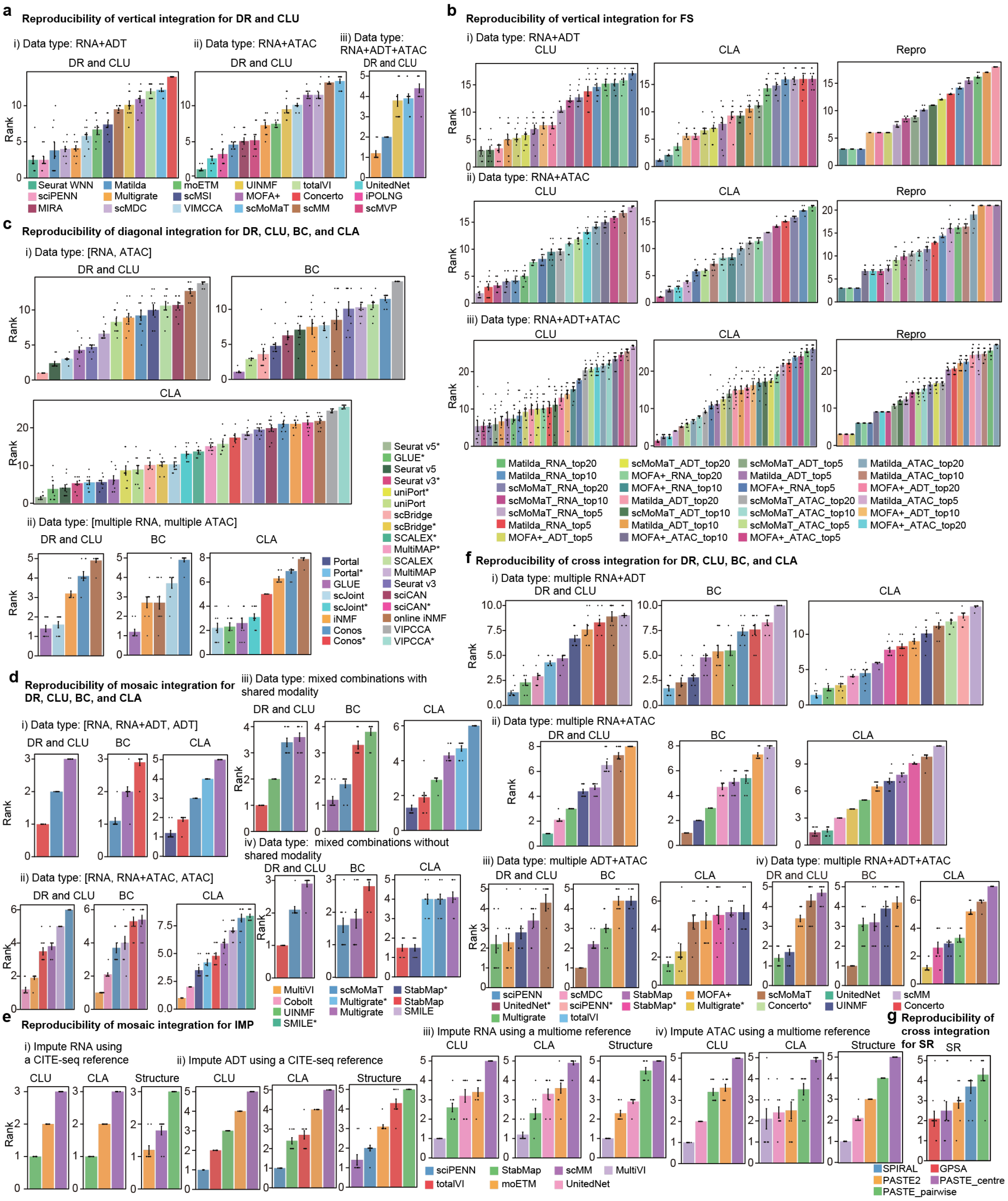

Sup Fig. 10: Robustness and consistency evaluation under random removal of ~20% cell types repeated 10 times (n=10 independent experiments). a, Vertical integration for dimension reduction and clustering tasks. **b**, Vertical integration for feature selection task. **c**, Diagonal integration for dimension reduction, clustering, batch correction, and classification tasks. **d**, Mosaic integration for dimension reduction, clustering, batch correction, and classification tasks. **e**, mosaic integration for imputation task. **f**, cross integration for dimension reduction, clustering, batch correction, and classification tasks. **g**, Cross integration for spatial registration. Each data point (a rank of a method in a dataset) is shown by a dot and the standard errors are shown by the error bars.

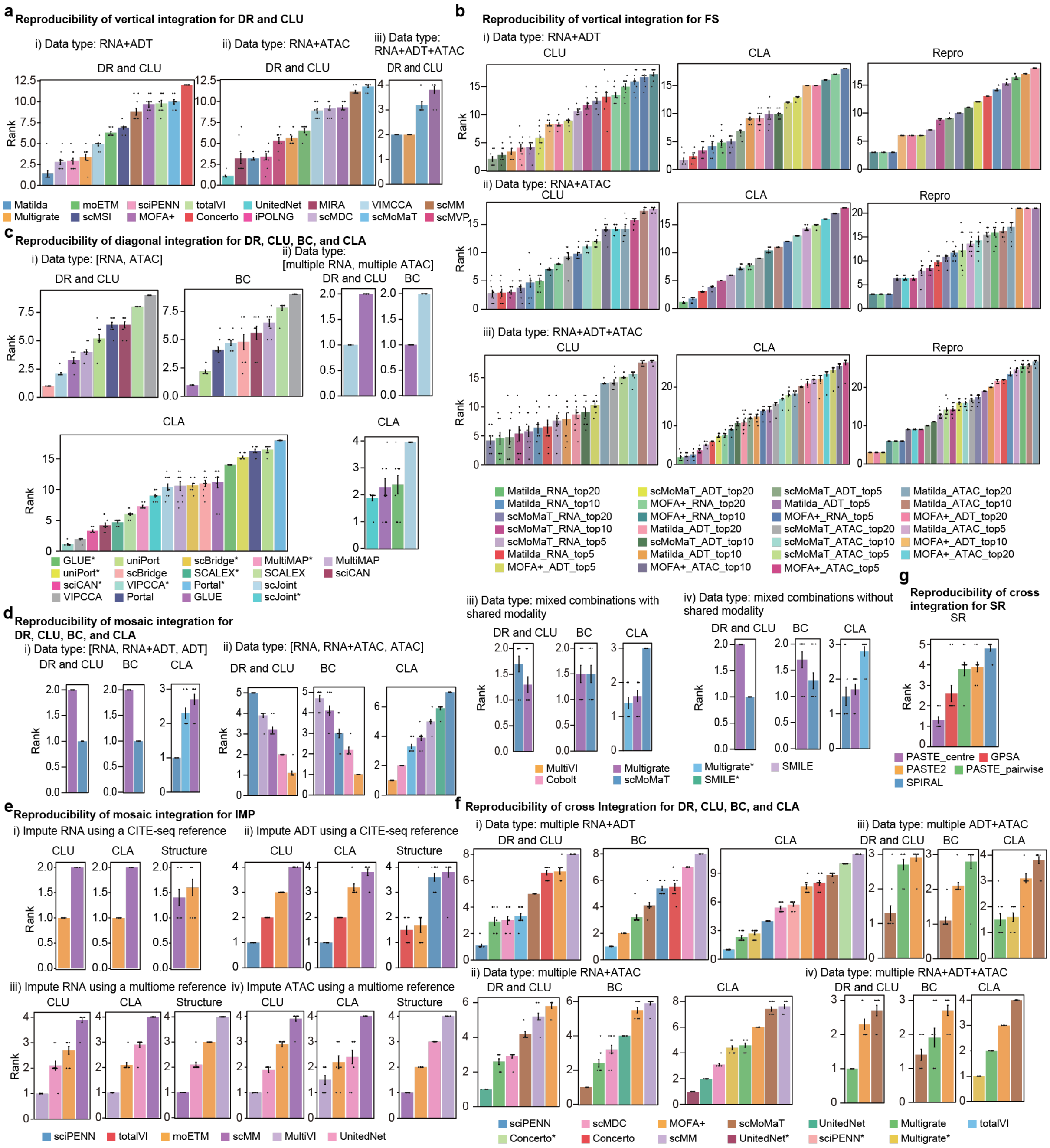

Sup Fig. 11: Robustness and consistency evaluation under random seeds repeated 10 times (n=10 independent experiments). **a**, Vertical integration for dimension reduction and clustering tasks. **b**, Vertical integration for feature selection task. **c**, Diagonal integration for dimension reduction, clustering, batch correction, and classification tasks. **d**, Mosaic integration for dimension reduction, clustering, batch correction, and classification tasks. **e**, Mosaic integration for imputation task. **f**, Cross integration for dimension reduction, clustering, batch correction, and classification tasks. **g**, Cross integration for spatial registration. Each data point (a rank of a method in a dataset) is shown by a dot and the standard errors are shown by the error bars.

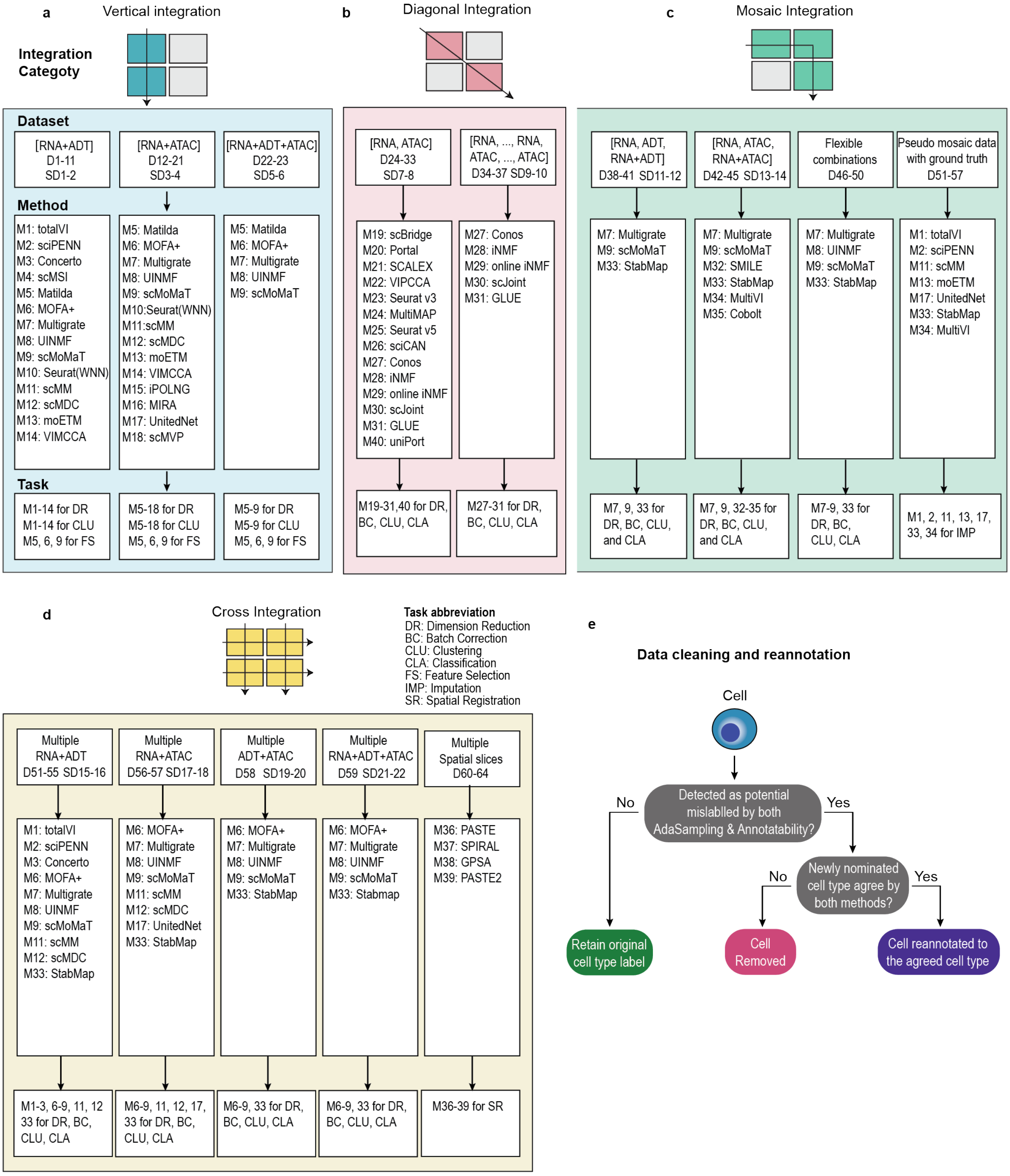

Sup Fig. 12: Summary of the 40 integration methods included in the benchmark evaluation. Methods are grouped by their integration category including **a**, Vertical integration, **b**, Diagonal integration, **c**, Mosaic integration, and **d**, Cross integration. Datasets and tasks that are evaluated are indexed. **e**, Data cleaning and reannotation procedure for quality control cell type annotation in each dataset.

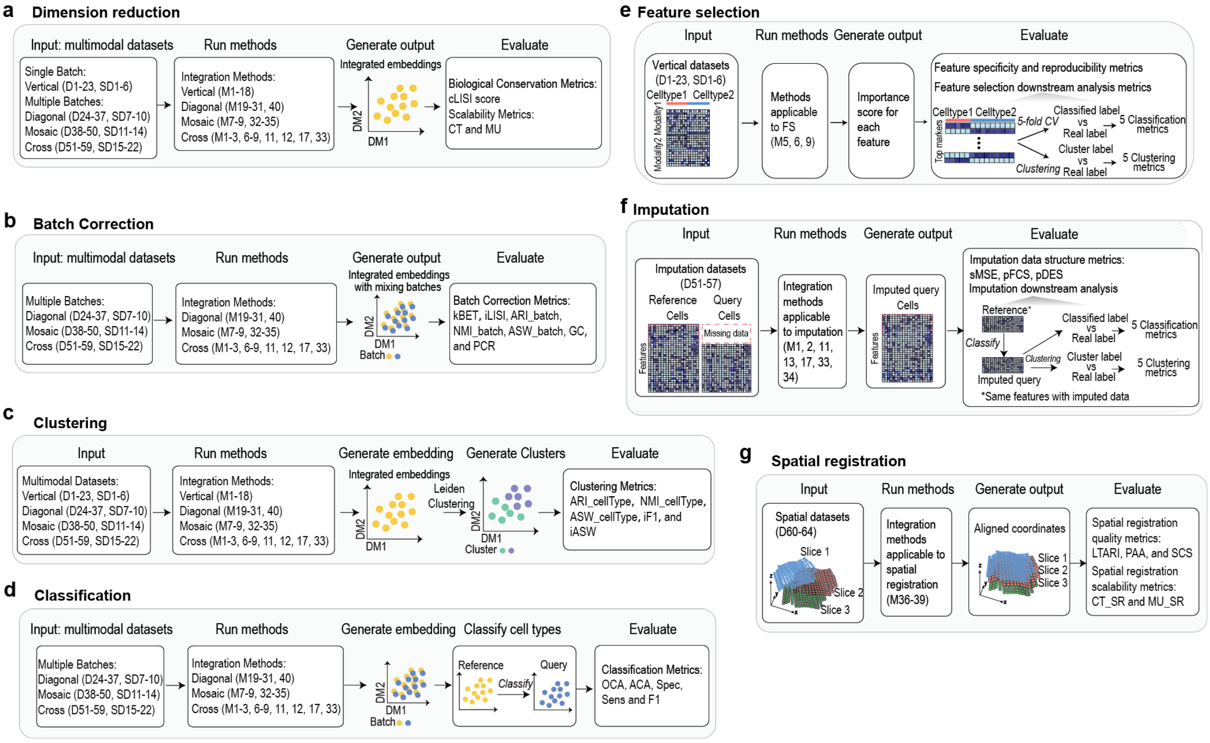

Sup Fig. 13: Schematic summary of the evaluation pipelines for the integration methods. a, Dimension reduction task. **b**, Batch correction task. **c**, Clustering task. **d**, Classification task. **e**, Feature selection task. **f**, Imputation task. **g**, Spatial registration task.

References

1. Gayoso, A. *et al.* Joint probabilistic modeling of single-cell multi-omic data with totalVI. *Nat Methods* **18**, 272–282 (2021).

2. Lakkis, J. *et al.* A multi-use deep learning method for CITE-seq and single-cell RNA-seq data integration with cell surface protein prediction and imputation. *Nat Mach Intell* **4**, 940–952 (2022).

3. Yang, M. *et al.* Contrastive learning enables rapid mapping to multimodal single-cell atlas of multimillion scale. *Nat Mach Intell* **4**, 696–709 (2022).

4. Zhang, C. *et al.* Contrastively generative self-expression model for single-cell and spatial multimodal data. *Brief Bioinform* **24**, bbad265 (2023).

5. Liu, C., Huang, H. & Yang, P. Multi-task learning from multimodal single-cell omics with Matilda. *Nucleic Acids Research* **51**, (2023).

6. Argelaguet, R. *et al.* MOFA+: a statistical framework for comprehensive integration of multi-modal single-cell data. *Genome Biol* **21**, 111 (2020).

7. Lotfollahi, M., Litinetskaya, A. & Theis, F. J. Multigrate: single-cell multi-omic data integration. 2022.03.16.484643 Preprint at https://doi.org/10.1101/2022.03.16.484643 (2022).
8. Kriebel, A. R. & Welch, J. D. UINMF performs mosaic integration of single-cell multi-omic datasets using nonnegative matrix factorization. *Nat Commun* **13**, 780 (2022).
9. Zhang, Z. *et al.* scMoMaT jointly performs single cell mosaic integration and multi-modal bio-marker detection. *Nat Commun* **14**, 384 (2023).
10. Hao, Y. *et al.* Integrated analysis of multimodal single-cell data. *Cell* **184**, 3573-3587.e29 (2021).
11. Minoura, K., Abe, K., Nam, H., Nishikawa, H. & Shimamura, T. scMM: Mixture-of-experts multimodal deep generative model for single-cell multiomics data analysis. 2021.02.18.431907 Preprint at https://doi.org/10.1101/2021.02.18.431907 (2021).
12. Lin, X., Tian, T., Wei, Z. & Hakonarson, H. Clustering of single-cell multi-omics data with a multimodal deep learning method. *Nat Commun* **13**, 7705 (2022).
13. Zhou, M. *et al.* Single-cell multi-omics topic embedding reveals cell-type-specific and COVID-19 severity-related immune signatures. *Cell Reports Methods* **3**, 100563 (2023).
14. Wang, Y. *et al.* A multi-view latent variable model reveals cellular heterogeneity in complex tissues for paired multimodal single-cell data. *Bioinformatics* **39**, btad005 (2023).
15. Zhang, W. & Lin, Z. iPoLNG—An unsupervised model for the integrative analysis of single-cell multiomics data. *Frontiers in Genetics* **14**, (2023).
16. Lynch, A. W. *et al.* MIRA: joint regulatory modeling of multimodal expression and chromatin accessibility in single cells. *Nat Methods* **19**, 1097–1108 (2022).
17. Tang, X. *et al.* Explainable multi-task learning for multi-modality biological data analysis. *Nat Commun* **14**, 2546 (2023).
18. Li, G. *et al.* A deep generative model for multi-view profiling of single-cell RNA-seq and ATAC-seq data. *Genome Biology* **23**, 20 (2022).
19. Li, Y. *et al.* scBridge embraces cell heterogeneity in single-cell RNA-seq and ATAC-seq data integration. *Nat Commun* **14**, 6045 (2023).
20. Zhao, J. *et al.* Adversarial domain translation networks for integrating large-scale atlas-level single-cell datasets. *Nat Comput Sci* **2**, 317–330 (2022).

21. Xiong, L. *et al.* Online single-cell data integration through projecting heterogeneous datasets into a common cell-embedding space. *Nat Commun* **13**, 6118 (2022).

22. Hu, J., Chen, M. & Zhou, X. Effective and scalable single-cell data alignment with non-linear canonical correlation analysis. *Nucleic Acids Research* **50**, e21–e21 (2022).

23. Stuart, T. *et al.* Comprehensive Integration of Single-Cell Data. *Cell* **177**, 1888-1902.e21 (2019).

24. Jain, M. S. *et al.* MultiMAP: dimensionality reduction and integration of multimodal data. *Genome Biology* **22**, 346 (2021).

25. Hao, Y. *et al.* Dictionary learning for integrative, multimodal and scalable single-cell analysis. *Nat Biotechnol* 1–12 (2023) doi:10.1038/s41587-023-01767-y.

26. Xu, Y., Begoli, E. & McCord, R. P. sciCAN: single-cell chromatin accessibility and gene expression data integration via cycle-consistent adversarial network. *npj Syst Biol Appl* **8**, 1–10 (2022).

27. Barkas, N. *et al.* Joint analysis of heterogeneous single-cell RNA-seq dataset collections. *Nat Methods* **16**, 695–698 (2019).

28. Welch, J. D. *et al.* Single-Cell Multi-omic Integration Compares and Contrasts Features of Brain Cell Identity. *Cell* **177**, 1873-1887.e17 (2019).

29. Gao, C. *et al.* Iterative single-cell multi-omic integration using online learning. *Nat Biotechnol* **39**, 1000–1007 (2021).

30. Lin, Y. *et al.* scJoint integrates atlas-scale single-cell RNA-seq and ATAC-seq data with transfer learning. *Nat Biotechnol* **40**, 703–710 (2022).

31. Cao, Z.-J. & Gao, G. Multi-omics single-cell data integration and regulatory inference with graph-linked embedding. *Nat Biotechnol* **40**, 1458–1466 (2022).

32. Xu, Y., Das, P. & McCord, R. P. SMILE: mutual information learning for integration of single-cell omics data. *Bioinformatics* **38**, 476–486 (2022).

33. Ghazanfar, S., Guibentif, C. & Marioni, J. C. Stabilized mosaic single-cell data integration using unshared features. *Nat Biotechnol* 1–9 (2023) doi:10.1038/s41587-023-01766-z.

34. Ashuach, T. *et al.* MultiVI: deep generative model for the integration of multimodal data. *Nat Methods* **20**, 1222–1231 (2023).

35. Gong, B., Zhou, Y. & Purdom, E. Cobolt: integrative analysis of multimodal single-cell sequencing data. *Genome Biology* **22**, 351 (2021).

36. Zeira, R., Land, M., Strzalkowski, A. & Raphael, B. J. Alignment and integration of spatial transcriptomics data. *Nat Methods* **19**, 567–575 (2022).

37. Guo, T. *et al.* SPIRAL: integrating and aligning spatially resolved transcriptomics data across different experiments, conditions, and technologies. *Genome Biology* **24**, 241 (2023).

38. Jones, A., Townes, F. W., Li, D. & Engelhardt, B. E. Alignment of spatial genomics data using deep Gaussian processes. *Nat Methods* **20**, 1379–1387 (2023).

39. Liu, X., Zeira, R. & Raphael, B. J. PASTE2: Partial Alignment of Multi-slice Spatially Resolved Transcriptomics Data. *bioRxiv* 2023.01.08.523162 (2023) doi:10.1101/2023.01.08.523162.

40. Cao, K., Gong, Q., Hong, Y. & Wan, L. A unified computational framework for single-cell data integration with optimal transport. *Nat Commun* **13**, 7419 (2022).

41. Luecken, M. D. *et al.* Benchmarking atlas-level data integration in single-cell genomics. *Nat Methods* **19**, 41–50 (2022).

42. Rand, W. M. Objective Criteria for the Evaluation of Clustering Methods. *Journal of the American Statistical Association* **66**, 846–850 (1971).

43. Rousseeuw, P. J. Silhouettes: A graphical aid to the interpretation and validation of cluster analysis. *Journal of Computational and Applied Mathematics* **20**, 53–65 (1987).

44. Stephenson, E. *et al.* Single-cell multi-omics analysis of the immune response in COVID-19. *Nat Med* **27**, 904–916 (2021).

45. Ramaswamy, A. *et al.* Immune dysregulation and autoreactivity correlate with disease severity in SARS-CoV-2-associated multisystem inflammatory syndrome in children. *Immunity* **54**, 1083-1095.e7 (2021).

46. Aran, D. *et al.* Reference-based analysis of lung single-cell sequencing reveals a transitional profibrotic macrophage. *Nat Immunol* **20**, 163–172 (2019).

47. Luecken, M. D. *et al.* A sandbox for prediction and integration of DNA, RNA, and proteins in single cells. in (2021).

48. Buus, T. B. *et al.* Improving oligo-conjugated antibody signal in multimodal single-cell analysis.

eLife **10**, e61973 (2021).

49. Ma, S. *et al.* Chromatin Potential Identified by Shared Single-Cell Profiling of RNA and Chromatin. *Cell* **183**, 1103-1116.e20 (2020).

50. Chen, S., Lake, B. B. & Zhang, K. High-throughput sequencing of the transcriptome and chromatin accessibility in the same cell. *Nat Biotechnol* **37**, 1452–1457 (2019).

51. Argelaguet, R. *et al. Decoding Gene Regulation in the Mouse Embryo Using Single-Cell Multi-Omics*. http://biorxiv.org/lookup/doi/10.1101/2022.06.15.496239 (2022) doi:10.1101/2022.06.15.496239.

52. Zhu, K. *et al.* Multi-omic profiling of the developing human cerebral cortex at the single-cell level. *Sci. Adv.* **9**, eadg3754 (2023).

53. Mimitou, E. P. *et al.* Scalable, multimodal profiling of chromatin accessibility, gene expression and protein levels in single cells. *Nat Biotechnol* **39**, 1246–1258 (2021).

54. Swanson, E. *et al.* Simultaneous trimodal single-cell measurement of transcripts, epitopes, and chromatin accessibility using TEA-seq. *eLife* **10**, e63632 (2021).

55. Ji, A. L. *et al.* Multimodal Analysis of Composition and Spatial Architecture in Human Squamous Cell Carcinoma. *Cell* **182**, 497-514.e22 (2020).

56. Maynard, K. R. *et al.* Transcriptome-scale spatial gene expression in the human dorsolateral prefrontal cortex. *Nat Neurosci* **24**, 425–436 (2021).

57. Janesick, A. *et al.* High resolution mapping of the tumor microenvironment using integrated single-cell, spatial and in situ analysis. *Nat Commun* **14**, 8353 (2023).

58. Wang, M. *et al.* High-resolution 3D spatiotemporal transcriptomic maps of developing Drosophila embryos and larvae. *Developmental Cell* **57**, 1271-1283.e4 (2022).

59. Palla, G. *et al.* Squidpy: a scalable framework for spatial omics analysis. *Nat Methods* **19**, 171–178 (2022).

60. Allen, W. E., Blosser, T. R., Sullivan, Z. A., Dulac, C. & Zhuang, X. Molecular and spatial signatures of mouse brain aging at single-cell resolution. *Cell* **186**, 194-208.e18 (2023).

61. Zhang, D. *et al.* Spatial epigenome–transcriptome co-profiling of mammalian tissues. *Nature* **616**, 113–122 (2023).
