# Supplementary material for: Multitask benchmarking of single-cell multimodal omics integration methods
Source: Nat Methods. 2025 Oct 13;22(11):2449–60. doi: 10.1038/s41592-025-02856-3 (PMC12615258; doi:10.1038/s41592-025-02856-3)
Supplement: Supplementary file 2 — Reporting Summary [file 41592_2025_2856_MOESM2_ESM.pdf]

Corresponding author(s): Pengyi Yang

Last updated by author(s): May 19, 2025

Reporting Summary

Nature Portfolio wishes to improve the reproducibility of the work that we publish. This form provides structure for consistency and transparency in reporting. For further information on Nature Portfolio policies, see our Editorial Policies and the Editorial Policy Checklist.

Statistics

For all statistical analyses, confirm that the following items are present in the figure legend, table legend, main text, or Methods section.

n/a	Confirmed	
☐	☒	The exact sample size (n) for each experimental group/condition, given as a discrete number and unit of measurement
☐	☒	A statement on whether measurements were taken from distinct samples or whether the same sample was measured repeatedly
☒	☐	The statistical test(s) used AND whether they are one- or two-sided<br>*Only common tests should be described solely by name; describe more complex techniques in the Methods section.*
☒	☐	A description of all covariates tested
☒	☐	A description of any assumptions or corrections, such as tests of normality and adjustment for multiple comparisons
☐	☒	A full description of the statistical parameters including central tendency (e.g. means) or other basic estimates (e.g. regression coefficient) AND variation (e.g. standard deviation) or associated estimates of uncertainty (e.g. confidence intervals)
☒	☐	For null hypothesis testing, the test statistic (e.g. F, t, r) with confidence intervals, effect sizes, degrees of freedom and P value noted<br>*Give P values as exact values whenever suitable.*
☒	☐	For Bayesian analysis, information on the choice of priors and Markov chain Monte Carlo settings
☒	☐	For hierarchical and complex designs, identification of the appropriate level for tests and full reporting of outcomes
☐	☒	Estimates of effect sizes (e.g. Cohen's d, Pearson's r), indicating how they were calculated

Our web collection on statistics for biologists contains articles on many of the points above.

Software and code

Policy information about availability of computer code

Data collection	No software was used during data collection.
Data analysis	Analysis of benchmark results was performed in R and Python using standard packages. We have uploaded the source code to a GitHub repository at https://github.com/PYangLab/scMultiBench, including scripts for running the benchmarking methods, the evaluation pipeline, and generating the figures associated with the paper. The code is also archived and available via Zenodo at https://doi.org/10.5281/zenodo.15385334.<br><br>Key package versions:<br>scvi-tools (version 1.1.2), sciPENN (version 1.0.0), Concerto (github version ab1fc7f), scMSI (github version dffcbb2), Matilda (github version 7d71480), MOFA+ (version 1.6.0), Multigrate (version 0.0.2), UNIMF (version 2.0.1), scMoMaT (version 0.2.2), scMM (github version c5c8579), scMDC (github version 43b0c3a), moETM (github version c2eaa97), VIMCCA (version 0.5.6), iPOLNG (version 0.0.2), MIRA (version 2.1.0), UnitedNet (github version 3689da8), scMVP (github version fc61e4d), scBridge (github version ff17561), Portal (version 1.0.2), SCALEX (version 1.0.2), VIPCCA (version 0.2.7), Seurat (version 5.0.2), MultiMAP (github version 681e608), SMILE (github version a2e2ca6), sciCAN (github version ad71bba), Conos (version 1.5.2), iNMF (version 2.0.1), online iNMF (version 2.0.1), scJoint (github version cbbfa5d), GLUE (version 0.3.2), uniPort (version 1.2.2), MultiVI (version 1.1.2), StabMap (version 0.1.8), Cobolt (version 1.0.1), PASTE (version 1.4.0), GPSA (version 0.8), SPIRAL (version 1.0), PASTE2 (github version b71ec88), scib (version 1.1.4), limma (version 3.44.3), SingleR (version 1.4.0), Signac (version 1.14.0), celldex (version 1.0.0).

For manuscripts utilizing custom algorithms or software that are central to the research but not yet described in published literature, software must be made available to editors and reviewers. We strongly encourage code deposition in a community repository (e.g. GitHub). See the Nature Portfolio guidelines for submitting code & software for further information.

Data

Policy information about availability of data

All manuscripts must include a data availability statement. This statement should provide the following information, where applicable:

- Accession codes, unique identifiers, or web links for publicly available datasets
- A description of any restrictions on data availability
- For clinical datasets or third party data, please ensure that the statement adheres to our policy

All real single-cell and spatial transcriptomics datasets used in this benchmark were obtained from publicly available repositories, as described in Supplementary Table 4. These include CITE-seq datasets from ArrayExpress (E-MTAB-10026) and GEO (GSE166489, GSE164378, GSE194122), Zenodo (https://zenodo.org/records/6348128), and Software Heritage (https://archive.softwareheritage.org/browse/revision/1c7fcabb18a1971dc4d6e29bc3ed4f6f36b2361f/); 10x multiome datasets from GEO (GSE194122, GSE205117, GSE204684) and Zenodo (https://zenodo.org/records/6348128); SHARE-seq and SNARE-seq datasets from GEO (GSE140203, GSE126074); ASAP-seq, DOGMA-seq, and TEA-seq datasets from GEO (GSE156478, GSE158013); and spatial transcriptomics datasets including Visium (https://zenodo.org/records/6334774, https://github.com/raphael-group/paste_reproducibility/tree/main/data/DLPFC), Xenium (https://www.10xgenomics.com/products/xenium-in-situ/preview-dataset-human-breast), Stereo-seq (https://db.cngb.org/stomics/flysta3d/), MERFISH (https://cellxgene.cziscience.com/collections/31937775-0602-4e52-a799-b6acdd2bac2e), and Spatial ATAC-RNA (GEO: GSE205055). The processed input datasets used for benchmarking are available in a publicly accessible Figshare repository (https://figshare.com/articles/dataset/datasets/29035586?file=54438737).

Human research participants

Policy information about studies involving human research participants and Sex and Gender in Research.

Reporting on sex and gender	n/a
Population characteristics	n/a
Recruitment	n/a
Ethics oversight	n/a

Note that full information on the approval of the study protocol must also be provided in the manuscript.

Field-specific reporting

Please select the one below that is the best fit for your research. If you are not sure, read the appropriate sections before making your selection.

☒ Life sciences ☐ Behavioural & social sciences ☐ Ecological, evolutionary & environmental sciences

For a reference copy of the document with all sections, see nature.com/documents/nr-reporting-summary-flat.pdf

Life sciences study design

All studies must disclose on these points even when the disclosure is negative.

Sample size	No new data was generated as part of this study. The number of samples in each data source was provided by the original authors and we selected the samples whose data match the multi-modal data types covered in our study. The number of datasets in the study was selected to cover a representative sample of modality combinations and tissues.
Data exclusions	No data was excluded from the study.
Replication	Due to computational limitations, the main benchmark run was performed only once. However, to assess the influence of random seeds and the reproducibility of subsetting 80% of the cell types, we: (1) performed 10 rounds of random subsampling of the 80% cell type data and compared the rank summaries across methods for each task; and (2) conducted 10 rounds with different seeds to evaluate the impact of seed variation, where applicable.
Randomization	This is not relevant to our study because we do not include separate experimental groups.
Blinding	This is not relevant to our study because we do not include separate experimental groups.

Reporting for specific materials, systems and methods

We require information from authors about some types of materials, experimental systems and methods used in many studies. Here, indicate whether each material, system or method listed is relevant to your study. If you are not sure if a list item applies to your research, read the appropriate section before selecting a response.

Materials & experimental systems

n/a	Involved in the study
☒	☐ Antibodies
☒	☐ Eukaryotic cell lines
☒	☐ Palaeontology and archaeology
☒	☐ Animals and other organisms
☒	☐ Clinical data
☒	☐ Dual use research of concern

Methods

n/a	Involved in the study
☒	☐ ChIP-seq
☒	☐ Flow cytometry
☒	☐ MRI-based neuroimaging
