# Supplementary material for: Multitask benchmarking of single-cell multimodal omics integration methods
Source: Nat Methods. 2025 Oct 13;22(11):2449–60. doi: 10.1038/s41592-025-02856-3 (PMC12615258; doi:10.1038/s41592-025-02856-3)
Supplement: Supplementary file 3 — Peer Review File [file 41592_2025_2856_MOESM3_ESM.pdf]

nature portfolio

Peer Review File

Multi-task benchmarking of single-cell multimodal omics integration methods

Corresponding Author: Professor Pengyi Yang

Version 0:

Decision Letter:

31st Oct 2023

Dear Professor Yang,

Thank you for your inquiry about submitting your manuscript, "Multi-task benchmarking of multimodal single-cell integration methods" to Nature Methods. The paper sounds like it may be of interest, and should fit the scope of the journal. We would be willing to consider it for publication in Nature Methods.

Of course, it is very difficult to judge a paper based only on the limited information available in a presubmission inquiry. Therefore I am sure you understand that we cannot promise to send your paper out for peer review and must read it in its entirety before deciding if this would be suitable.

Our Registered Reports format is intended for systematic performance comparison studies of related, established methods or tools. We strongly encourage you to read our Editorial introducing this format and familiarize yourself with our guidelines for this format. As the purpose of a Registered Report is to provide authors with editor and reviewer feedback at the experimental design phase, Stage 1 manuscripts should be submitted prior to embarking on large-scale data collection (pilot data is fine and encouraged).

You will find our Guide to Authors at http://www.nature.com/naturemethods to assist you in preparing your manuscript. However, it is not necessary at this stage to spend major effort adhering to our detailed formatting instructions.

Thank you for your interest in Nature Methods.

Sincerely,

Allison Doerr, Ph.D.
Chief Editor
Nature Methods

Version 1:

Decision Letter:

25th Dec 2023

Dear Professor Yang,

Thank you for submitting your manuscript entitled "Multi-task benchmarking of single-cell multimodal omics integration methods". We have given the paper our careful consideration and find it of potential interest. However, we are concerned that sending the current manuscript out to review could lead to unnecessary delays and possibly an undesirable outcome of the review process.

In particular, please expand the details about your evaluation pipeline. If you are using a code pipeline for data analysis, please also provide this.

We therefore invite you to revise your manuscript to address these concerns before we make a final determination on whether to send your manuscript for external peer-review. Please ensure that the revised version is as concise as possible, and that it conforms to our format requirements (see http://www.nature.com/nmeth for our Guide to Authors).

We shall hope to receive your revised version as soon as you are able to complete the suggested revisions. If something similar is published in the interim we will have to consider the impact it has on the novelty of the revised manuscript.

If you anticipate a delay of more than 6 weeks, please let us know. In this event, we will still be happy to reconsider your paper at a later date so long as nothing similar has been accepted for publication at Nature Methods or published elsewhere. In the event of publication, however, the received date would be that of the revised rather than the original version.

Nature Methods is committed to improving transparency in authorship. As part of our efforts in this direction, we are now requesting that all authors identified as 'corresponding author' on published papers create and link their Open Researcher and Contributor Identifier (ORCID) with their account on the Manuscript Tracking System (MTS), prior to acceptance. This applies to primary research papers only. ORCID helps the scientific community achieve unambiguous attribution of all scholarly contributions. You can create and link your ORCID from the home page of the MTS by clicking on 'Modify my Springer Nature account'. For more information please visit please visit www.springernature.com/orcid.

If you are not interested in submitting a revised manuscript in the future please let me know immediately so we can close your file. If you have any questions, please contact me.

Please use the link below when you are prepared to resubmit.
Link Redacted

Note: The above URL links to your confidential home page and associated information about manuscripts you may have submitted, or that you are reviewing for us. If you wish to forward this email to co-authors, please delete the link to your homepage.

Thank you for your interest in Nature Methods. We wish you a happy new year.

Sincerely,

Lin Tang, PhD
Senior Editor
Nature Methods

** For Nature Portfolio general information and news for authors, see http://npg.nature.com/authors.

Version 2:

Decision Letter:

1st Apr 2024

Dear Professor Yang,

Thank you very much for your patience when your Registered Report Stage 1 proposal, "Multi-task benchmarking of single-cell multimodal omics integration methods" is sent out for peer review. This proposal has now been evaluated by expert reviewers. As you will see from their comments below, although the reviewers find your work of potential interest, they have raised several suggestions and concerns. We are interested in the possibility of proceeding further with your submission at Nature Methods, but would like to consider your response to these concerns before we reach a final decision on acceptance in principle and Stage 2 submission.

I have discussed the reviewer reports in detail with our chief editor and other members of the Nature Methods team, with a view to 1) identifying key priorities that should be addressed in revision, and 2) overruling reviewer requests that we think fall beyond the scope of the study. Please see the attached file which contains the reviewer reports annotated with our editorial recommendations. We invite you to revise your Stage 1 proposal to address these concerns (before you embark on the proposed experiments).

We are committed to providing a fair and constructive peer review process. Do not hesitate to contact us if there are any reviewer requests that you believe are technically impossible or unlikely to yield a meaningful outcome.

When revising your paper:
* include a point-by-point response to the reviewers and to any editorial suggestions
* please underline/highlight any additions to the text or areas with other significant changes to facilitate review of the revised manuscript

* include a cover letter with the following information

---an anticipated timeline for completing the study (should the study be accepted in principle)
---a statement confirming that, should the study be accepted in principle, you agree to share your raw data and conform to our other open science requirements (see details below)
---a statement confirming that, following acceptance in principle, you agree to register the Stage 1 proposal in a recognized repository (either publicly or under private embargo), until submission of the Stage 2 manuscript (please note we can assist you in uploading your Stage 1 proposal in our Figshare space)
---a statement confirming that if you later decide to withdraw your manuscript from consideration at Nature Methods, you agree to the journal publishing a brief note about the withdrawn proposal

Please resubmit all the necessary files electronically by using the link below to access your home page:

Link Redacted

IMPORTANT: This URL links to your confidential home page and associated information about manuscripts you may have submitted, or that you are reviewing for us. If you wish to forward this email to co-authors, please delete the link to your homepage.

We hope to receive your revised proposal within 6 weeks. If you cannot send it within this time, please let us know. In this event, we will still be happy to reconsider your paper at a later date so long as nothing similar has been accepted for publication at Nature Methods or published elsewhere.

ORCID
Nature Methods is committed to improving transparency in authorship. As part of our efforts in this direction, we are now requesting that all authors identified as ‘corresponding author’ on published papers create and link their Open Researcher and Contributor Identifier (ORCID) with their account on the Manuscript Tracking System (MTS), prior to acceptance. This applies to primary research papers only. ORCID helps the scientific community achieve unambiguous attribution of all scholarly contributions. You can create and link your ORCID from the home page of the MTS by clicking on ‘Modify my Springer Nature account’. For more information please visit please visit www.springernature.com/orcid.

Please do not hesitate to contact me if you have any questions or would like to discuss these revisions further. We look forward to seeing the revised manuscript and thank you for the opportunity to consider your work.

Sincerely,

Lin Tang, PhD
Senior Editor
Nature Methods

Reviewers' Comments:
Reviewer #1:
Remarks to the Author:
Liu et al. proposed a comprehensive benchmarking analysis plan to assess the performance of various types of single-cell multi-modal data integration methods. With the increasing prevalence of single-cell multi-modal data, numerous integration methods have been developed, posing a challenge for practitioners in choosing the most suitable approach. This paper seeks to address this challenge by conducting a comprehensive evaluation that involves a large number of methods and datasets. A unique aspect of this paper is the evaluation of different types of multimodal omics data integration, including “vertical”, “diagonal”, “mosaic”, and “cross” integration. It is the first time that such comprehensive evaluation is being proposed. Additionally, the authors outlined a detailed timeline for the completion of this paper. Overall, this is a timely paper. The completion of this paper will offer practical guidance to researchers when deciding which method to use for single-cell multi-modal data integration.

I have a few comments about the papers.

1. Potential bias in evaluation: An inherent challenge in benchmarking analyses lies in defining the ground truth. The authors should clearly describe what information is treated as ground truth. The authors should also discuss alternative evaluation strategies when ground truth is hard to define.
2. It is good that the authors included spatial transcriptomics integration evaluation, but the two proposed datasets are both based on Visium platform. As the field is now moving towards single-cell resolution spatial omics platforms, it is important for the authors to conduct evaluations using those spatial omics data that have single-cell resolution.
3. For the spatial transcriptomics integration, the authors only considered registration of different tissue slices. While results from this evaluation will offer useful information, it seems a bit out of scope. The focus of the paper is multi-modal data integration, but the proposed registration evaluation only uses gene expression. A more appropriate evaluation is to perform integration of multi-modal spatial omics data similar to the way that single-cell multi-omics data integration is evaluated. This evaluation would better align with the paper’s objectives.

Remarks on code availability:
The website is well documented.

Reviewer #2:
Remarks to the Author:
This registered report proposes to comprehensively benchmark multimodal omics integration methods. The authors have proposed benchmarking across four different types of integration problems: vertical, diagonal, mosaic, and cross. They propose to evaluate these methods using 7 different downstream tasks, including dimension reduction and classification, using 53 different datasets. Overall the proposed work appears to be comprehensive in the number of different methods and datasets it proposes to use, and a systematic and unbiased evaluation of these methods on a variety of datasets would certainly be a useful resource for the community.

My main concerns relate to the evaluation metrics, the dataset processing, and the vertical integration category.

How the authors will evaluate performance of methods is a crucial point in the benchmarking study. Several different metrics have been proposed here, mostly from the scIB work. However, these metrics need to be more carefully considered. For example, the LISI metric does not take into account differences in cell type abundance between samples, and so the expected diversity of batches in each neighborhood is not taken into account by the metric. It is better if the authors can focus on a smaller but well-motivated set of evaluation metrics. Many of these metrics also require a “ground truth” cell type annotation, and it is unclear how this will be obtained. Simply using the author-supplied cell type annotations for datasets can bias the results towards methods that simply produce results more similar to those used to process the data by the original authors. Wherever possible, the authors should use multimodal datasets (eg, those in the “cross” integration) to enable a comparison between, for example, diagonal integration performance vs same-modality integration performance. This would allow setting the “ground truth” to the results from within-modality integration and assess how closely each cross-modality method is able to recapitulate these results.

How the results of benchmarking will be finally communicated was also unclear. Will methods be ranked, or the results for each separate metric and dataset simply stated? Do the authors aim to provide a final recommendation for readers, and how would this be determined?

In the description of datasets to be used, it is stated that peaks found in <10% of ATAC cells will be removed. However, this can remove peaks that are specific to certain low-abundance cell types and would likely impact the results. Furthermore, it was unclear how the ATAC data will be processed. Will the same set of peaks be quantified for all datasets being integrated? If so, what quantification method will be used? The number of peaks stated in most ATAC datasets also seems very low. ATAC data usually has around 100k peaks, but most of these datasets have <10k peaks, some <3k (D19 for example), which seems unusually low. For the spatial data, only Visium data is proposed. To be comprehensive it would be better to include some other methods, including molecule-resolution (eg, Xenium, MERFISH).

The vertical integration category is conceptually quite different from the others, as it aims to place one dataset in a low dimensional space (or build a graph) that captures variation across cells present in all the measured modalities. There is no goal of batch correction since this deals with a single dataset. The methods for evaluating the performance of these methods are therefore quite different as batch correction metrics do not apply. I would suggest the authors instead focus only on diagonal, mosaic, and cross integration methods for their evaluation. Furthermore, Seurat WNN for vertical integration does not generate latent space but generates a graph. This would make it difficult or not possible to evaluate the performance using some of the proposed metrics.

For the list of software methods, there are too many to check that the code and version proposed for each one is appropriate. I did notice that for Seurat v5 bridge integration, this was added in the version 5 release, so v4.1.1.9001 should not be used. The latest Seurat v5 release should be used instead (5.0.2). I would suggest, as much as possible, contacting the authors of the methods with the information about the version and example code that you intend to run and give them an opportunity to give feedback. This will ensure that the results are not biased simply due to running the methods incorrectly.

Other comments:
- For Seurat v3, it should be specified what parameters will be used as this can influence results substantially (RPCA vs CCA)

- For vertical integration (if retained in the benchmarking) the authors should consider including some more multiome datasets. There are many on the 10x website from a variety of tissues, as well as in publications.

- The CreateGeneActivityMatrix function has been removed in later releases of Seurat in favor of the GeneActivity function in the Signac package.

- The gc() function in R will free memory through garbage collection. A better approach to record the peak memory usage could be the /usr/bin/time command.

- Should add robustness to missing cell types. Perturb datasets by removing cell types and see how integration performance changes.

- The feature selection part was unclear. How are top markers defined? The differential expression results should only depend on the populations of cells being compared, so this should in a sense be captured by the evaluation of the integrated low-dimension space and cell type classification accuracy.

Reviewer #3:
Remarks to the Author:
The authors have proposed to conduct a benchmarking study on 37 single-cell multimodal omics integration methods through seven different analytic tasks. Overall, the experimental plan is comprehensive and my main comments are listed below:

1. It would be helpful to list the methods that did not pass the eight selection criteria and what criteria did they fail.

2. A key to successful benchmarking is the existence of underlying ground truth. Here, the cell type labels obtained from the original publications are treated as ground truth for comparing cell type clustering methods. However, these labels are obtained through specific bioinformatics software and thus will inevitably favor methods that are similar to these existing bioinformatics software. As a result, it seems that the proposed benchmarking study will not be able to evaluate the performance of different methods, but rather the similarities of different methods to the original bioinformatics pipeline used in individual studies. How do you resolve this issue?

3. For some of the chosen tasks, it would be helpful to provide intuitive explanations as to why those evaluation metrics can be used to reflect method performance. For example, cLISI score is proposed to evaluate method performance on dimension reduction. The use of cLISI score appears to imply that methods that can better separate cell types are considered to perform better in dimension reduction. But this should be justified and explicitly stated.

4. What are the expected new insights gained through the present study as compared to existing benchmarking studies on the same topic (e.g. https://doi.org/10.1093/bib/bbae095; https://doi.org/10.1038/s41467-020-20430-7; and https://doi.org/10.1186/s13059-022-02739-2)?

5. The Leiden clustering method is used for all clustering tasks and other tasks requires clustering. Do other clustering methods influence the conclusion of these comparisons? For the Leiden clustering method, how do you determine the resolution parameter that implicitly controls the number of resulting clusters?

6. A large number of the compared methods use algorithms that can be highly sensitive to the initial values or random seeds. How do you evaluate the influence of initial values and random seeds on the performance comparison of different methods?

7. How do you evaluate the consistency of different methods across different technologies and platforms?

****

OPEN SCIENCE REQUIREMENTS

REPORTING SUMMARY AND EDITORIAL POLICY CHECKLISTS
When revising your manuscript, please update your reporting summary and editorial policy checklists.

Reporting summary: https://www.nature.com/documents/nr-reporting-summary.zip
Editorial policy checklist: https://www.nature.com/documents/nr-editorial-policy-checklist.zip

If your paper includes custom software, we also ask you to complete a supplemental reporting summary.

Software supplement: https://www.nature.com/documents/nr-software-policy.pdf

Please submit these with your revised manuscript. They will be available to reviewers to aid in their evaluation if the paper is re-reviewed. If you have any questions about the checklist, please see http://www.nature.com/authors/policies/availability.html or contact me.

Please note that these forms are dynamic 'smart pdfs' and must therefore be downloaded and completed in Adobe Reader. We will then flatten them for ease of use by the reviewers. If you would like to reference the guidance text as you complete the template, please access these flattened versions at http://www.nature.com/authors/policies/availability.html.

IMAGE INTEGRITY
When submitting the revised version of your manuscript, please pay close attention to our Digital Image Integrity Guidelines and to the following points below:

-- that unprocessed scans are clearly labelled and match the gels and western blots presented in figures.
-- that control panels for gels and western blots are appropriately described as loading on sample processing controls
-- all images in the paper are checked for duplication of panels and for splicing of gel lanes.

Finally, please ensure that you retain unprocessed data and metadata files after publication, ideally archiving data in perpetuity, as these may be requested during the peer review and production process or after publication if any issues arise.

DATA AVAILABILITY
We strongly encourage you to deposit all new data associated with the paper in a persistent repository where they can be freely and enduringly accessed. We recommend submitting the data to discipline-specific and community-recognized repositories; a list of repositories is provided here: http://www.nature.com/sdata/policies/repositories

All novel DNA and RNA sequencing data, protein sequences, genetic polymorphisms, linked genotype and phenotype data, gene expression data, macromolecular structures, and proteomics data must be deposited in a publicly accessible database, and accession codes and associated hyperlinks must be provided in the “Data Availability” section.

Refer to our data policies here: https://www.nature.com/nature-research/editorial-policies/reporting-standards#availability-of-data

To further increase transparency, we encourage you to provide, in tabular form, the data underlying the graphical representations used in your figures. This is in addition to our data-deposition policy for specific types of experiments and large datasets. For readers, the source data will be made accessible directly from the figure legend. Spreadsheets can be submitted in .xls, .xlsx or .csv formats. Only one (1) file per figure is permitted: thus if there is a multi-paneled figure the source data for each panel should be clearly labeled in the csv/Excel file; alternately the data for a figure can be included in multiple, clearly labeled sheets in an Excel file. File sizes of up to 30 MB are permitted. When submitting source data files with your manuscript please select the Source Data file type and use the Title field in the File Description tab to indicate which figure the source data pertains to.

Please include a “Data availability” subsection in the Online Methods. This section should inform readers about the availability of the data used to support the conclusions of your study, including accession codes to public repositories, references to source data that may be published alongside the paper, unique identifiers such as URLs to data repository entries, or data set DOIs, and any other statement about data availability. At a minimum, you should include the following statement: “The data that support the findings of this study are available from the corresponding author upon request”, describing which data is available upon request and mentioning any restrictions on availability. If DOIs are provided, please include these in the Reference list (authors, title, publisher (repository name), identifier, year). For more guidance on how to write this section please see: http://www.nature.com/authors/policies/data/data-availability-statements-data-citations.pdf

CODE AVAILABILITY
Please include a “Code Availability” subsection in the Online Methods which details how your custom code is made available. Only in rare cases (where code is not central to the main conclusions of the paper) is the statement “available upon request” allowed (and reasons should be specified).

We request that you deposit code in a DOI-minting repository such as Zenodo, Gigantum or Code Ocean and cite the DOI in the Reference list. We also request that you use code versioning and provide a license.

For more information on our code sharing policy and requirements, please see:
https://www.nature.com/nature-research/editorial-policies/reporting-standards#availability-of-computer-code

MATERIALS AVAILABILITY
As a condition of publication in Nature Methods, authors are required to make unique materials promptly available to others without undue qualifications.

Authors reporting new chemical compounds must provide chemical structure, synthesis and characterization details. Authors reporting mutant strains and cell lines are strongly encouraged to use established public repositories.

More details about our materials availability policy can be found at https://www.nature.com/nature-portfolio/editorial-policies/reporting-standards#availability-of-materials

Version 3:

Decision Letter:

30th Jul 2024

Dear Professor Yang,

Thank you for sending the updated version of your Stage 1 Registered Report, entitled "Multi-task benchmarking of single-cell

multimodal omics integration methods." After consulting again with Reviewer 2, I am delighted to say that we can offer acceptance in principle. You may progress to Stage 2 and complete your study as approved.

As you know, a condition of acceptance-in-principle is that the authors agree to deposit their Stage 1 accepted manuscript in a repository, either publicly or under embargo until Stage 2 manuscript acceptance and publication. We are very keen to showcase our accepted-in-principle manuscripts, so that our readers, reviewers, and potential authors can gain insight into the requirements of the format as well as an idea of the types of projects that are suitable for publication as Registered Reports in Nature Methods. We have set up a space on figshare (https://springernature.figshare.com/registered-reports_nmethods) to host all of our accepted-in-principle manuscripts, which can either be made public or kept under embargo until Stage 2 acceptance (depending on author preference). This gives you the opportunity to have your work publicly associated with Nature Methods, and of course we will be very pleased to showcase your report if you agree to share it publicly.

If you agree with posting your Stage 1 manuscript on our figshare space, we will upload it on your behalf and either set it public or place it under embargo, depending on your choice. Your protocol will be licensed under a CC BY license (Creative Commons Attribution 4.0 International License). The CC BY license allows for maximum dissemination and re-use of open access materials and is preferred by many research funding bodies. Under this license users are free to share (copy, distribute and transmit) and remix (adapt) the contribution including for commercial purposes, providing they attribute the contribution in the manner specified by the author or licensor (read full legal code: http://creativecommons.org/licenses/by/4.0/legalcode) Please note that any use of https://springernature.figshare.com will be subject to the figshare terms of use. Figshare has the right to enforce these terms and conditions where applicable. Use of third party services and sites will be subject to the relevant terms of use and will apply if we act on your behalf in this regard. Do let me know if you would like to take up this option or if you have any questions regarding the manuscript deposition requirement.

Please also note that depositing the work on our figshare space does not preclude deposition of your Stage 1 manuscript on other repositories – your manuscript can also be posted on any other public repository of your choice.

Following completion of your study, we invite you to resubmit your finalized manuscript as a Stage 2 Registered Report. We will send the Stage 2 manuscript to our reviewers for a final check, but they will be instructed that any comments on novelty and/or potential significance of the results will not factor into our final decision.

IMPORTANT: Please note that your manuscript can still be rejected for publication at Stage 2 if the Editors consider any of the following to hold:

-- The authors substantially alter the rationale for the study as approved in the Stage 1 submission (please note that the Introduction should not be significantly modified from the Stage 1 manuscript).
-- The authors fail to adhere closely to the approved experimental plan. (Please contact us as soon as possible for advice if at any point you need to make any changes to your experimental plan.)
-- The authors' conclusions are not justified given the data obtained.
--Any post hoc (unregistered) analyses are not justified or are overly dominant in shaping the authors' conclusions.
-- Our open science requirements (detailed below) are not followed.

Should authors choose to withdraw their Registered Report at any time, we will publish a Withdrawn Registration notice.

When you are ready, please use the following link to access your home page and submit your Stage 2 Registered Report:

Link Redacted

Note: This URL links to your confidential home page and associated information about manuscripts you may have submitted, or that you are reviewing for us. If you wish to forward this email to co-authors, please delete the link to your homepage.

We expect your Stage 2 Registered Report to be submitted by the date specified in your latest cover letter. If unforeseen circumstances prevent submission by that date, please contact us as soon as possible.

Thank you again for submitting your work to Nature Methods and we look forward to receiving your Stage 2 Registered Report! Please do not hesitate to reach out to me at any time if you have questions.

Yours sincerely,

Lin Tang, PhD
Senior Editor
Nature Methods

OPEN SCIENCE REQUIREMENTS

REPORTING SUMMARY AND EDITORIAL POLICY CHECKLISTS
When submitting your Stage 2 manuscript, please include a reporting summary and editorial policy checklists. If you have any questions about these checklist, please see http://www.nature.com/authors/policies/availability.html or contact me.

Reporting summary: https://www.nature.com/documents/nr-reporting-summary.zip
Editorial policy checklist: https://www.nature.com/documents/nr-editorial-policy-checklist.zip

If your paper includes custom software, we also ask you to complete a supplemental reporting summary.

Software supplement: https://www.nature.com/documents/nr-software-policy.pdf

Please note that these forms are dynamic 'smart pdfs' and must therefore be downloaded and completed in Adobe Reader. We will then flatten them for ease of use by the reviewers. If you would like to reference the guidance text as you complete the template, please access these flattened versions at http://www.nature.com/authors/policies/availability.html.

IMAGE INTEGRITY
When submitting the revised version of your manuscript, please pay close attention to our href="https://www.nature.com/nature-research/editorial-policies/image-integrity">Digital Image Integrity Guidelines. and to the following points below:

-- that unprocessed scans are clearly labelled and match the gels and western blots presented in figures.
-- that control panels for gels and western blots are appropriately described as loading on sample processing controls
-- all images in the paper are checked for duplication of panels and for splicing of gel lanes.

Finally, please ensure that you retain unprocessed data and metadata files after publication, ideally archiving data in perpetuity, as these may be requested during the peer review and production process or after publication if any issues arise.

DATA AVAILABILITY
We strongly encourage you to deposit all new data associated with the paper in a persistent repository where they can be freely and enduringly accessed. We recommend submitting the data to discipline-specific and community-recognized repositories; a list of repositories is provided here: http://www.nature.com/sdata/policies/repositories

All novel DNA and RNA sequencing data, protein sequences, genetic polymorphisms, linked genotype and phenotype data, gene expression data, macromolecular structures, and proteomics data must be deposited in a publicly accessible database, and accession codes and associated hyperlinks must be provided in the "Data Availability" section.

Refer to our data policies here: https://www.nature.com/nature-research/editorial-policies/reporting-standards#availability-of-data

To further increase transparency, we encourage you to provide, in tabular form, the data underlying the graphical representations used in your figures. This is in addition to our data-deposition policy for specific types of experiments and large datasets. For readers, the source data will be made accessible directly from the figure legend. Spreadsheets can be submitted in .xls, .xlsx or .csv formats. Only one (1) file per figure is permitted: thus if there is a multi-paneled figure the source data for each panel should be clearly labeled in the csv/Excel file; alternately the data for a figure can be included in multiple, clearly labeled sheets in an Excel file. File sizes of up to 30 MB are permitted. When submitting source data files with your manuscript please select the Source Data file type and use the Title field in the File Description tab to indicate which figure the source data pertains to.

Please include a "Data availability" subsection in the Online Methods. This section should inform readers about the availability of the data used to support the conclusions of your study, including accession codes to public repositories, references to source data that may be published alongside the paper, unique identifiers such as URLs to data repository entries, or data set DOIs, and any other statement about data availability. At a minimum, you should include the following statement: "The data that support the findings of this study are available from the corresponding author upon request", describing which data is available upon request and mentioning any restrictions on availability. If DOIs are provided, please include these in the Reference list (authors, title, publisher (repository name), identifier, year). For more guidance on how to write this section please see:
http://www.nature.com/authors/policies/data/data-availability-statements-data-citations.pdf

CODE AVAILABILITY
Please include a "Code Availability" subsection in the Online Methods which details how any custom code is made available. Only in rare cases (where code is not central to the main conclusions of the paper) is the statement "available upon request" allowed (and reasons should be specified).

We request that you deposit code in a DOI-minting repository such as Zenodo, Gigantum or Code Ocean and cite the DOI in the Reference list. We also request that you use code versioning and provide a license.

For more information on our code sharing policy and requirements, please see:
https://www.nature.com/nature-research/editorial-policies/reporting-standards#availability-of-computer-code

MATERIALS AVAILABILITY
As a condition of publication in Nature Methods, authors are required to make unique materials promptly available to others without undue qualifications.

Authors reporting new chemical compounds must provide chemical structure, synthesis and characterization details. Authors reporting mutant strains and cell lines are strongly encouraged to use established public repositories.

More details about our materials availability policy can be found here.

ORCID
Nature Methods is committed to improving transparency in authorship. As part of our efforts in this direction, we are now requesting that all authors identified as 'corresponding author' on published papers create and link their Open Researcher and Contributor Identifier (ORCID) with their account on the Manuscript Tracking System (MTS), prior to acceptance. This applies to primary research papers only. ORCID helps the scientific community achieve unambiguous attribution of all scholarly contributions. You can create and link your ORCID from the home page of the MTS by clicking on 'Modify my Springer Nature account'. For more information please visit please visit www.springernature.com/orcid.

TRANSPARENT PEER REVIEW
Nature Methods offers a transparent peer review option for new original research manuscripts submitted from 17th February 2021. We encourage increased transparency in peer review by publishing the reviewer comments, author rebuttal letters and editorial decision letters if the authors agree. Such peer review material is made available as a supplementary peer review file. **Please state in the cover letter 'I wish to participate in transparent peer review' if you want to opt in, or 'I do not wish to participate in transparent peer review' if you don't.** Failure to state your preference will result in delays in accepting your manuscript for publication.
Please note: we allow redactions to authors' rebuttal and reviewer comments in the interest of confidentiality. If you are concerned about the release of confidential data, please let us know specifically what information you would like to have removed. Please note that we cannot incorporate redactions for any other reasons. Reviewer names will be published in the peer review files if the reviewer signed the comments to authors, or if reviewers explicitly agree to release their name. For more information, please refer to our FAQ page.

****

Reviewers' Comments:

Reviewer #1:
Remarks to the Author:
My previous concerns have been appropriately addressed. I don't have any additional comments.

Remarks on code availability
The authors provided a well documented website for programs, data, and evaluation metrics.

Reviewer #2:
Remarks to the Author:
The authors have addressed some of my initial comments regarding dataset processing, additional spatial and multiome datasets, and communication of results. The proposal to use AdaSampling and Annotatability to improve the annotation of cells certainly may help to reduce the bias towards methods more similar to those used to produce the original cell annotations, but does not fully address this issue.

The inclusion of a shiny app is a good addition, but I would also think that the authors aim to provide some conclusions about the relative performance of methods in their paper. It's still unclear how final recommendations would be made.

It was still unclear how the ATAC data will be processed, particularly what methods/software would be used to quantify the peaks in each dataset.

Remarks on code availability
The github repo has not been updated for the revison, and does not appear to include the scripts to be used for each software tool.

Reviewer #3:
Remarks to the Author:
My previous comments have been well addressed.

Version 4:

Decision Letter:

Our ref: NMETH-RR54299D

13th Apr 2025

Dear Dr. Yang,

Thank you for submitting your revised manuscript "Multi-task benchmarking of single-cell multimodal omics integration methods" (NMETH-RR54299D). It has now been seen by the original referees and their comments are below. The reviewers find that the paper has improved in revision, and therefore we'll be happy in principle to publish it in Nature Methods, pending minor revisions to satisfy the referees' final requests and to comply with our editorial and formatting guidelines.

When submitting a revised version of the manuscript, please also supply a point-by-point response letter.

We think point 5 in Reviewer 1's comments is optional and you can discuss it in the Discussion section. If you plan to add any new analysis results, they should be labeled as "Exploratory analysis" (the same applies to results of any new analysis not included in the Stage 1 Registered Report manuscript).

Among other revisions, please clarify the benchmarking of SMILE/MaxFuse. If those tools were not included in the Stage 1 Registered Report manuscript, they should be labeled as "Exploratory analysis".

We are now performing detailed checks on your paper and will send you a checklist detailing our editorial and formatting requirements within two weeks or so. Please do not upload the final materials and make any revisions until you receive this additional information from us.

TRANSPARENT PEER REVIEW
Nature Methods offers a transparent peer review option for new original research manuscripts submitted from 17th February 2021. We encourage increased transparency in peer review by publishing the reviewer comments, author rebuttal letters and editorial decision letters if the authors agree. Such peer review material is made available as a supplementary peer review file. **Please state in the cover letter 'I wish to participate in transparent peer review' if you want to opt in, or 'I do not wish to participate in transparent peer review' if you don't.** Failure to state your preference will result in delays in accepting your manuscript for publication.

Please note: we allow redactions to authors' rebuttal and reviewer comments in the interest of confidentiality. If you are concerned about the release of confidential data, please let us know specifically what information you would like to have removed. Please note that we cannot incorporate redactions for any other reasons. Reviewer names will be published in the peer review files if the reviewer signed the comments to authors, or if reviewers explicitly agree to release their name. For more information, please refer to our FAQ page.

ORCID
IMPORTANT: Non-corresponding authors do not have to link their ORCIDs but are encouraged to do so. Please note that it will not be possible to add/modify ORCIDs at proof. Thus, please let your co-authors know that if they wish to have their ORCID added to the paper they must follow the procedure described in the following link prior to acceptance:
https://www.springernature.com/gp/researchers/orcid/orcid-for-nature-research

Thank you again for your interest in Nature Methods. Please do not hesitate to contact me if you have any questions. We will be in touch again soon.

Sincerely,

Lin Tang, PhD
Senior Editor
Nature Methods

Reviewer #1 (Remarks to the Author):

1. The authors have conducted a comprehensive evaluation across various data types, demonstrating that method performance is highly dataset- and modality-dependent. This result is expected, as no single method can consistently outperform others in all scenarios. However, my main concern is that the primary figures only report the overall performance of each method, aggregated across all datasets. This may be misleading. I strongly recommend that the authors to use a box plot or a similar visualization to allow readers to assess method performance across different datasets more intuitively.
2. How was the performance across different datasets combined? Was it averaged? I believe aggregating performance across datasets is not ideal, as it obscures dataset-specific variations. While this paper follows a common benchmarking format, I question the effectiveness of this approach, particularly given the strong dependence of performance on dataset and modality.
3. The main figures demonstrate results from a single selected dataset. Could the authors clarify the criteria used for selecting

this dataset?
4. The authors discuss MaxFuse and SMILE, but these methods were not included in the evaluation. Please incorporate them into the benchmarking analysis.
5. I appreciate the inclusion of spatial data in the paper; however, the registration task appears somewhat disconnected from the main focus. I suggest aligning the spatial data integration strategy more closely with that used for single-cell data to ensure a more coherent analysis. Moreover, registration is only applicable to data derived from the same tissue, making it a special case rather than a broadly applicable approach in real-world studies. To improve consistency, I recommend revising the integration strategy for spatial data so that it aligns more closely with the evaluations throughout the rest of the paper.

Reviewer #2 (Remarks to the Author):

The authors have faithfully followed the benchmarking plan that was proposed in stage 1, and present a very detailed benchmarking study of the various data integration tasks for single cell analysis. The scope is impressive, both in the datasets and range of methods evaluated, and this will be a valuable resource for the community. The major results are nicely summarized in Fig9, which will be a useful guide for users in selecting methods. The discussion in appropriate, and highlights some of the limitations in the metrics and the existence of tradeoffs between different objectives (batch correction, biological conservation). The shiny app is a nice addition and can be helpful to explore the results in more detail.

Reviewer #3 (Remarks to the Author):

The authors adhered closely to the experimental plan outlined in the Stage 1 submission and conducted a comprehensive benchmarking of multimodal omics integration methods across a broad collection of datasets and evaluation metrics. The analyses are accurate and statistically rigorous. I have only a few minor comments for the authors to consider:

1. Each results section begins by describing a specific dataset example, followed by a summary of the overall results and rankings. Would it be clearer to reverse this order—presenting the overall findings first, then illustrating them with representative examples?

2. Related to the above, it would be helpful to clarify the criteria used to select the specific datasets featured in the main text. Were these chosen based on dataset complexity, performance variability, or other considerations?

3. On page 11, in the section "Benchmarking methods on data imputation for mosaic data analysis," the term structural similarity is used. Could the authors clarify what this term refers to in this context? Additionally, how should readers interpret the finding that structural similarity does not always align with clustering or classification performance?

4. In the benchmarking of spatial registration methods, PASTE2—though a newer development built on PASTE—exhibited worse overall performance. Could the authors provide some discussion on why this might be the case?

5. In the section "Benchmarking vertical integration methods for dimension reduction and clustering tasks," the manuscript suggests that performance on simulated datasets may not align with performance on real datasets. Does this indicate that the simulated datasets lack key characteristics of real data? If so, should their utility in benchmarking be interpreted with caution?

Version 5:

Decision Letter:

8th Sep 2025

Dear Professor Yang,

Thank you very much for your patience when we check the revised version of your Registered Reports "Multi-task benchmarking of single-cell multimodal omics integration methods". I am pleased to inform you that this paper has now been accepted for publication in Nature Methods. The received and accepted dates will be 10th Dec 2023 and 8th Sep 2025. This note is intended to let you know what to expect from us over the next month or so, and to let you know where to address any further questions.

Over the next few weeks, your paper will be copyedited to ensure that it conforms to Nature Methods style. Once your paper is typeset, you will receive an email with a link to choose the appropriate publishing options for your paper and our Author Services team will be in touch regarding any additional information that may be required. It is extremely important that you let us know now whether you will be difficult to contact over the next month. If this is the case, we ask that you send us the contact information (email, phone and fax) of someone who will be able to check the proofs and deal with any last-minute problems.

After the grant of rights is completed, you will receive a link to your electronic proof via email with a request to make any corrections within 48 hours. If, when you receive your proof, you cannot meet this deadline, please inform us at rjsproduction@springernature.com immediately.

Authors may need to take specific actions to achieve compliance with funder and institutional open access mandates. If your research is supported by a funder that requires immediate open access (e.g. according to Plan S principles or the NIH public access policy) then you should select the gold OA route, and we will direct you to the compliant route where possible. Because authors warrant under our subscription licensing terms that they haven't committed to licensing any version of their article under a licence inconsistent with the terms of our agreement – including the applicable embargo period – publication under the subscription model isn't suitable for authors whose funders require no embargo.

If you have any questions about our publishing options, costs, Open Access requirements, or our legal forms, please contact ASJournals@springernature.com

If you have posted a preprint on any preprint server, please ensure that the preprint details are updated with a publication reference, including the DOI and a URL to the published version of the article on the journal website.

You may wish to make your media relations office aware of your accepted publication, in case they consider it appropriate to organize some internal or external publicity. Once your paper has been scheduled you will receive an email confirming the publication details. This is normally 3-4 working days in advance of publication. If you need additional notice of the date and time of publication, please let the production team know when you receive the proof of your article to ensure there is sufficient time to coordinate. Further information on our embargo policies can be found here: https://www.nature.com/authors/policies/embargo.html

To assist our authors in disseminating their research to the broader community, our SharedIt initiative provides you with a unique shareable link that will allow anyone (with or without a subscription) to read the published article. Recipients of the link with a subscription will also be able to download and print the PDF.

As soon as your article is published, you will receive an automated email with your shareable link.

You can now use a single sign-on for all your accounts, view the status of all your manuscript submissions and reviews, access usage statistics for your published articles and download a record of your refereeing activity for the Nature journals.

Please note that you and any of your coauthors will be able to order reprints and single copies of the issue containing your article through Nature Portfolio's reprint website, which is located at http://www.nature.com/reprints/author-reprints.html. If there are any questions about reprints please send an email to author-reprints@nature.com and someone will assist you.

Please feel free to contact me if you have questions about any of these points. Thank you very much again for publishing your paper at Nature Methods!

Best regards,

Lin Tang, PhD
Senior Editor
Nature Methods

** Visit the Springer Nature Editorial and Publishing website at www.springernature.com/editorial-and-publishing-jobs for more information about our career opportunities. If you have any questions please click here.**

Open Access This Peer Review File is licensed under a Creative Commons Attribution 4.0 International License, which permits use, sharing, adaptation, distribution and reproduction in any medium or format, as long as you give appropriate credit to the original author(s) and the source, provide a link to the Creative Commons license, and indicate if changes were made. In cases where reviewers are anonymous, credit should be given to 'Anonymous Referee' and the source.
The images or other third party material in this Peer Review File are included in the article's Creative Commons license, unless indicated otherwise in a credit line to the material. If material is not included in the article's Creative Commons license and your intended use is not permitted by statutory regulation or exceeds the permitted use, you will need to obtain permission directly from the copyright holder.
To view a copy of this license, visit https://creativecommons.org/licenses/by/4.0/

Editor recommendations

Reviewer 1 has concerns on the ground truth in the evaluation, and suggests strengthening the spatial integration evaluation using single-cell resolution spatial omics data and multi-modal spatial omics data. We agree with these comments and think they should be well addressed.

Reviewer 2 raises concerns and questions on the evaluation metrics (and ground truth), dataset processing and other aspects of the proposal (some are shared with other reviewers). Please fully address all these important points.

We think Reviewer 3's comments are important and helpful (some are shared with other reviewers, such as the ones about ground truth) and need to be fully addressed to improve this proposal.

We acknowledge that cell type annotations provided by the authors from their original studies may not be ground truth. To address this, we propose to perform data cleaning and reannotation using AdaSampling (PMID: 29993676) and Annotability (https://doi.org/10.1101/2024.04.06.588373) techniques followed by manual verification to identify and correct the labels of potentially mislabeled cells in the experimental data. We have now included the details of these proposed procedures in the revised manuscript (section "Data cleaning and reannotation" and Figure 2e). We also propose to include the generation of simulation data with ground truth for method assessment. There are very few multimodal single-cell omics data simulation methods and most of them require cell type labels from the original data as input for modelling and generating simulation data. To this end, we propose to use generalised versions of SymSim (PMID: 31197158) as described in (PMID: 35761403) and (PMID: 36693837) for simulating multimodal data, including RNA+ATAC, RNA+ADT, ADT+ATAC, and RNA+ADT+ATAC modalities, without using cell type labels from the original data. Details are included in the revised proposal (section "Simulation datasets").

Besides the above-proposed approaches for addressing the concerns on the potential bias of ground truth, we note that the diverse data sources we have acquired have been generated using various platforms that profiled different combinations of data modalities and by different labs from different samples. In particular, each data source used a unique set of cell type annotation procedures and bioinformatics approaches and most studies have included various manual annotation strategies based on expert knowledge and cell type markers. We have now included this information in the revised proposal in section "Data availability" and Supplementary Table 4, which will assist in the interpretation of performance when a particular dataset and method/task is considered. Nonetheless, given that the performance of methods ultimately will not be judged by a single data source/set, we believe that the diverse data and cell type annotation strategies used in their original studies and our additional proposals to include data cleaning and verification and data simulation will provide an overview of multimodal single-cell data integration methods across a range of datasets, settings, and tasks, and be informative for their future development and adoption by the research community.

Reviewer 1

Liu et al. proposed a comprehensive benchmarking analysis plan to assess the performance of various types of single-cell multi-modal data integration methods. With the increasing prevalence of single-cell multi-modal data, numerous integration methods have been developed, posing a challenge for practitioners in choosing the most suitable approach. This paper seeks to address this challenge by conducting a comprehensive evaluation that involves a large number of methods and datasets. A unique aspect of this paper is the evaluation of different types of multimodal omics data integration, including "vertical", "diagonal", "mosaic", and "cross" integration. It is the first time that such comprehensive evaluation is being proposed. Additionally, the authors outlined a detailed timeline for the completion of this paper. Overall, this is a timely paper. The completion of this paper will offer practical guidance to researchers when deciding which method to use for single-cell multi-modal data integration. I have a few comments about the papers.

Response: We thank the reviewer for the positive comments and constructive suggestions.

1. Potential bias in evaluation: An inherent challenge in benchmarking analyses lies in defining the ground truth. The authors should clearly describe what information is treated as ground truth. The authors should also discuss alternative evaluation strategies when ground truth is hard to define.

Response: We agree with the reviewer that the use of the cell type annotations from their original studies may introduce potential bias to the benchmarking analyses. We propose to address this challenge by the following approaches. First, we will perform data cleaning and reannotation using AdaSampling (PMID: 29993676) and Annotability (https://doi.org/10.1101/2024.04.06.588373) techniques followed by manual inspection for further verification. The proposed procedures are now included in the revised proposal (section "Data cleaning and reannotation" and Figure 2e). In addition, we propose to include the generation of simulation data. Very few methods can simulate multimodal single-cell omics data and most of them require cell type labels from the original data for modelling and generating simulation data. To this end, we propose to use generalised versions of SymSim (PMID: 31197158) as described in (PMID: 35761403) and (PMID: 36693837) for simulating multimodal data, including RNA+ATAC, RNA+ADT, ADT+ATAC, and RNA+ADT+ATAC modalities, without using cell type labels from the original data. We have included these experimental details in a new section "Simulation datasets". Finally, we have also included the details of the cell type annotation procedure used by each experimental data source (section "Data availability", and Supplementary Table 4). We anticipate the evaluation across a broad range of experimental data sources accompanied by data simulation and data cleaning and verification will help reduce the potential bias in cell type annotation from a single dataset and provide an informative benchmark of integration method for single-cell multimodal omics data.

2. It is good that the authors included spatial transcriptomics integration evaluation, but the two proposed datasets are both based on the Visium platform. As the field is now

moving towards single-cell resolution spatial omics platforms, it is important for the authors to conduct evaluations using those spatial omics data that have single-cell resolution.

Response: We thank this suggestion and have now included in the proposal additional datasets generated from Xenium, MERFISH, and Stereo-seq technologies, which profile spatial transcriptomics at single-cell and subcellular resolutions. These details are included in sections “Datasets for spatial integration” and “Data availability”, and Supplementary Table 1 and 4.

3. For the spatial transcriptomics integration, the authors only considered registration of different tissue slices. While results from this evaluation will offer useful information, it seems a bit out of scope. The focus of the paper is multi-modal data integration, but the proposed registration evaluation only uses gene expression. A more appropriate evaluation is to perform integration of multi-modal spatial omics data similar to the way that single-cell multi-omics data integration is evaluated. This evaluation would better align with the paper’s objectives.

Response: As suggested, we have now included in the proposal multimodal spatial omics data for method evaluation. Details of these data are now presented in section “Data availability” and Supplementary Table 4.

Remarks on code availability:
The website is well documented.

Response: We thank this comment.

Reviewer 2

This registered report proposes to comprehensively benchmark multimodal omics integration methods. The authors have proposed benchmarking across four different types of integration problems: vertical, diagonal, mosaic, and cross. They propose to evaluate these methods using 7 different downstream tasks, including dimension reduction and classification, using 53 different datasets. Overall the proposed work appears to be comprehensive in the number of different methods and datasets it proposes to use, and a systematic and unbiased evaluation of these methods on a variety of datasets would certainly be a useful resource for the community.

Response: We thank the positive review and constructive suggestions.

My main concerns relate to the evaluation metrics, the dataset processing, and the vertical integration category.

1. How the authors will evaluate performance of methods is a crucial point in the benchmarking study. Several different metrics have been proposed here, mostly from the

scIB work. However, these metrics need to be more carefully considered. For example, the LISI metric does not take into account differences in cell type abundance between samples, and so the expected diversity of batches in each neighborhood is not taken into account by the metric. It is better if the authors can focus on a smaller but well-motivated set of evaluation metrics. Many of these metrics also require a “ground truth” cell type annotation, and it is unclear how this will be obtained. Simply using the author-supplied cell type annotations for datasets can bias the results towards methods that simply produce results more similar to those used to process the data by the original authors. Wherever possible, the authors should use multimodal datasets (eg, those in the “cross” integration) to enable a comparison between, for example, diagonal integration performance vs same-modality integration performance. This would allow setting the “ground truth” to the results from within-modality integration and assess how closely each cross-modality method is able to recapitulate these results.

Response: We thank the reviewer for these suggestions. We have included a new Supplementary Table 3 introducing each of all metrics and summarising their advantages, disadvantages (such as the LISI metric that does not account for the variance in cell type abundance), requirements for cell type and batch labels, applicability to graph or embedding outputs, and other information. We consider that using a comprehensive set of metrics provides a holistic view of integration performance, highlighting diverse strengths and weaknesses potentially overlooked with fewer metrics. We have also tested using the within-modality integration results to establish “ground truth” for assessing methods that integrate across multiple modalities but have found that the outputs from within-modality integration methods do not necessarily lead to high-quality results. We propose to address the requirement of the ground truth of cell types by performing cleaning and reannotation on experimental data using AdaSampling (PMID: 29993676) and Annotability (https://doi.org/10.1101/2024.04.06.588373) techniques and also manual inspection for further verification. These procedures are now included in the revised proposal (section “Data cleaning and reannotation” and Figure 2e). We also propose to generate simulation data using generalised versions of SymSim (PMID: 31197158) as described in (PMID: 35761403) and (PMID: 36693837) for simulating multimodal data, including RNA+ATAC, RNA+ADT, ADT+ATAC, and RNA+ADT+ATAC modalities, without using cell type labels from the original data (section “Simulation datasets”). These experiments will help alleviate the potential bias in the “ground truth” of cell type annotation and will provide useful information on method performance. Finally, we have now also included the cell type annotation procedures used by each data source (section “Data availability” and Supplementary Table 4). These details provide information such as the use of particular algorithms and/or manual annotation for cell type annotation, which will help when interpreting the performance of a method on a particular dataset.

2. How the results of benchmarking will be finally communicated was also unclear. Will methods be ranked, or the results for each separate metric and dataset simply stated? Do the authors aim to provide a final recommendation for readers, and how would this be determined?

Response: We will create an R Shiny application to host the assessment results. This will enable researchers to select integration categories, tasks, evaluation metrics, and datasets to dynamically display the relative performance of methods. It will also facilitate the inclusion of new methods and datasets when these become available. We have added this communication of results in the revised proposal (section "R Shiny application for interactive exploration of benchmark results").

3. In the description of datasets to be used, it is stated that peaks found in <10% of ATAC cells will be removed. However, this can remove peaks that are specific to certain low-abundance cell types and would likely impact the results. Furthermore, it was unclear how the ATAC data will be processed. Will the same set of peaks be quantified for all datasets being integrated? If so, what quantification method will be used? The number of peaks stated in most ATAC datasets also seems very low. ATAC data usually has around 100k peaks, but most of these datasets have <10k peaks, some <3k (D19 for example), which seems unusually low. For the spatial data, only Visium data is proposed. To be comprehensive it would be better to include some other methods, including molecule-resolution (eg, Xenium, MERFISH).

Response: We thank the reviewer for these suggestions. We have now revised the proposal to filter out peaks identified in less than 1% of ATAC cells. The updated numbers of ATAC peaks for all datasets are included in Supplementary Table 1. We employ the 'reduce' function within the Signac package to consolidate intersecting peaks from all data batches. This quantification method ensures that the same set of peaks is quantified for all datasets being integrated. We have now included these details in the revised manuscript (section 'Experimental datasets'). As suggested, we have now included additional spatial omics datasets in the proposal from those generated using Xenium, MERFISH, and Stereo-seq technologies, which profile spatial transcriptomics at single-cell or subcellular resolutions. Details of these additional data are included in sections "Datasets for spatial integration" and "Data availability", and Supplementary Table 1 and 4.

4. The vertical integration category is conceptually quite different from the others, as it aims to place one dataset in a low dimensional space (or build a graph) that captures variation across cells present in all the measured modalities. There is no goal of batch correction since this deals with a single dataset. The methods for evaluating the performance of these methods are therefore quite different as batch correction metrics do not apply. I would suggest the authors instead focus only on diagonal, mosaic, and cross integration methods for their evaluation. Furthermore, Seurat WNN for vertical integration does not generate latent space but generates a graph. This would make it difficult or not possible to evaluate the performance using some of the proposed metrics.

Response: We thank this comment. We consider vertical integration a key application for methods designed for single-cell multimodal omics data integration and therefore relevant to our proposed benchmark study. We note that no study has yet benchmarked methods for vertical integration, highlighting the utility and importance of including this integration category in our analysis. We have now included these details in Supplementary Table 2

regarding the outputs of the methods (either embedding or graph). Furthermore, we have included in Supplementary Table 3 the descriptions of each evaluation metric and their advantages, disadvantages, required ground truth, applicability to graph or embedding outputs, and other information.

5. For the list of software methods, there are too many to check that the code and version proposed for each one is appropriate. I did notice that for Seurat v5 bridge integration, this was added in the version 5 release, so v4.1.1.9001 should not be used. The latest Seurat v5 release should be used instead (5.0.2). I would suggest, as much as possible, contacting the authors of the methods with the information about the version and example code that you intend to run and give them an opportunity to give feedback. This will ensure that the results are not biased simply due to running the methods incorrectly.

Response: Thank you for the valuable suggestion. We have updated the Seurat version 5. Additionally, we have reached out to all authors of the methods we have included in the proposed benchmark, providing example code and asking for their feedback on whether the implementation and the choice of parameters are appropriate. We have now revised the proposal based on all feedback we have received at the time of this resubmission.

Other comments:

- For Seurat v3, it should be specified what parameters will be used as this can influence results substantially (RPCA vs CCA)

Response: We have now clarified this in the revised proposal (section "Single-cell multimodal omics data integration methods"). Specifically, the reduction parameter in the 'FindTransferAnchors' function is set to 'cca' by default (as suggested by the authors), following the tutorial outlined at https://satijalab.org/seurat/articles/seurat5_atacseq_integration_vignettel.

- For vertical integration (if retained in the benchmarking) the authors should consider including some more multiome datasets. There are many on the 10x website from a variety of tissues, as well as in publications.

Response: We have added six additional datasets (D16-D21) from another multiome data source (PMID: 37824614) in our proposal (section "Datasets for vertical integration" and Supplementary Table 1). This together provides 23 real datasets for vertical integration.

- The CreateGeneActivityMatrix function has been removed in later releases of Seurat in favor of the GeneActivity function in the Signac package.

Response: We thank this comment and will use the 'GeneActivity' function in the Signac package. This has been updated in the proposal.

- The gc() function in R will free memory through garbage collection. A better approach to record the peak memory usage could be the /usr/bin/time command.

Response: We have revised the proposal, replacing the ‘gc()’ function with the ‘/usr/bin/time -v command’ to record peak memory usage.

- Should add robustness to missing cell types. Perturb datasets by removing cell types and see how integration performance changes.

Response: We thank this suggestion and have included in the proposal a “Robustness and consistency assessment” section, where as suggested we will randomly remove a subset of cell types from datasets to evaluate how integration performance changes.

- The feature selection part was unclear. How are top markers defined? The differential expression results should only depend on the populations of cells being compared, so this should in a sense be captured by the evaluation of the integrated low-dimension space and cell type classification accuracy.

Response: Top markers for each cell type will be identified by their ranking and/or feature importance scores as determined by each method. While both the cell type makers and the low-dimensional embeddings can be used for cell type classification, the former directly uses the features from the original feature space and improves the interpretability for various downstream analysis. This makes them a unique and commonly performed task in single-cell omics data analysis. We have now clarified this in the revised proposal (section “Feature selection”).

Reviewer 3

The authors have proposed to conduct a benchmarking study on 37 single-cell multimodal omics integration methods through seven different analytic tasks. Overall, the experimental plan is comprehensive and my main comments are listed below:

Response: We thank the reviewer for the positive comments and constructive suggestions.

1. It would be helpful to list the methods that did not pass the eight selection criteria and what criteria did they fail.

Response: We thank this suggestion. We have now added details specifying the criteria for which methods failed to fulfil. These are now included in Supplementary Table 2 “Criteria Not Met”.

2. A key to successful benchmarking is the existence of underlying ground truth. Here, the cell type labels obtained from the original publications are treated as ground truth for comparing cell type clustering methods. However, these labels are obtained through specific bioinformatics software and thus will inevitably favor methods that are similar to these existing bioinformatics software. As a result, it seems that the proposed benchmarking study will not be able to evaluate the performance of different methods, but

rather the similarities of different methods to the original bioinformatics pipeline used in individual studies. How do you resolve this issue?

Response: We fully agree with the reviewer that high-quality ground truth is essential for successful benchmarking and acknowledge that the generation of cell type labels from their original publications relies on specific bioinformatics software that may introduce bias to the proposed benchmark. We aim to address this by performing data cleaning and reannotation using AdaSampling (PMID: 29993676) and Annotability (https://doi.org/10.1101/2024.04.06.588373) techniques and manual inspection for further verification. We have included the details of these proposed procedures in section "Data cleaning and reannotation" and Figure 2e. Furthermore, we prioritise the selection of experimental data generated from different data sources and diverse cell type annotation procedures, and complemented by annotations using expert knowledge of marker genes etc. These details are included in the revised proposal (section "Data availability" and Supplementary Table 4) and will help the interpretation of method performance on a particular dataset. In parallel, we also aim to generate simulation data by using generalised versions of SymSim (PMID: 31197158), a method that does not require cell type label information from the original data, as described in (PMID: 35761403) and (PMID: 36693837) for simulating multimodal data, including RNA+ATAC, RNA+ADT, ADT+ATAC, and RNA+ADT+ATAC data. Details are included in the section "Simulation datasets". We anticipate the evaluation across a broad range of data sources complemented with data cleaning and verification and data simulation will help reduce the potential bias in cell type annotation from any particular study and provide useful information on method performance.

3. For some of the chosen tasks, it would be helpful to provide intuitive explanations as to why those evaluation metrics can be used to reflect method performance. For example, cLISI score is proposed to evaluate method performance on dimension reduction. The use of cLISI score appears to imply that methods that can better separate cell types are considered to perform better in dimension reduction. But this should be justified and explicitly stated.

Response: We agree that it would be beneficial to provide intuitive explanations for why certain evaluation metrics reflect the performance of methods for chosen tasks. Indeed, cLISI score is proposed as a measure to evaluate the performance of dimension reduction techniques. A higher cLISI score implies that a method better distinguishes between cell types in the reduced dimensional space. This suggests that methods that separate cell types more effectively are considered to have better performance in dimension reduction as they can reduce data complexity while preserving critical biological information. We have included in Supplementary Table 3 a detailed description of evaluation metrics including each of their descriptions, advantages, disadvantages, required ground truth, applicability to graph or embedding outputs, and other information.

4. What are the expected new insights gained through the present study as compared to existing benchmarking studies on the same topic (e.g.
https://doi.org/10.1093/bib/bbae095;

https://doi.org/10.1038/s41467-020-20430-7; and
https://doi.org/10.1186/s13059-022-02739-2)?

Response: We thank the reviewer for directing us to these studies. The work by Xiao et al. (https://doi.org/10.1093/bib/bbae095) benchmarked methods specifically designed for single-cell RNA and ATAC data. Other data modalities such as ADT and their various combinations are not included in the assessment. Furthermore, the evaluation does not distinguish different categories of integrations given the focus was on RNA and ATAC modalities alone. The work by Cantini et al. (https://doi.org/10.1038/s41467-020-20430-7) benchmarked methods for multi-omics dimensionality reduction in cancer applications. The scope of this study differs significantly from us in that (i) it performed analyses/comparisons on traditional bulk omics data and is not targeted specifically on single-cell data, (ii) it included primarily methods for multi-omics data dimension reduction and does not distinguish different integration strategies, and (iii) it focused its comparisons/interpretations on cancer studies. Lastly, Leng et al. (https://doi.org/10.1186/s13059-022-02739-2) also benchmarked methods for integrating multi-omics data for cancer studies. In particular, they focused on only deep learning-based methods and therefore did not include those that are not considered as using deep learning models. Furthermore, similar to Cantini et al., this benchmark study by Leng et al. was not specifically for multimodal single-cell omics data analysis and focused on cancer studies for their comparisons.

For our proposed study, the comparison will be conducted particularly for methods designed for single-cell multimodal omics integration across four specific integration categories (e.g. vertical, diagonal, mosaic, and cross integrations), encompassing seven tasks, and using 64 real datasets and 22 simulated datasets. This will be the first study to provide a comprehensive view of methods designed for single-cell multimodal omics data integration on the four data integration categories, and tasks and modalities, that have not been previously identified and categorised. It will also provide a reference point and practical guidelines for method comparison and selection for single-cell multimodal omics data analyses. We have now related our proposed study to these related works in the revised manuscript and the expected new insights and outcomes that will be gained through this proposed work (section “Introduction”, paragraphs 2 and 3).

5. The Leiden clustering method is used for all clustering tasks and other tasks requires clustering. Do other clustering methods influence the conclusion of these comparisons? For the Leiden clustering method, how do you determine the resolution parameter that implicitly controls the number of resulting clusters?

Response: We thank the reviewer for this suggestion. We have now included in the proposal a new section “Clustering algorithms and parameters”, in which we propose to assess the performance using different popular clustering algorithms. For the clustering methods that require a resolution parameter, we will use the ‘get_N_clusters’ function from the Sinfonia package to automatically select the resolution parameters that align the number of clusters to the number of cell types in each dataset.

6. A large number of the compared methods use algorithms that can be highly sensitive to the initial values or random seeds. How do you evaluate the influence of initial values and random seeds on the performance comparison of different methods?

Response: We thank the reviewer for this suggestion. We have now included in the proposal a new section “Robustness and consistency assessment”, in which we propose to assess the performance of methods that rely on random initialisation (noted in Supplementary Table 2) by repeating them 10 times and evaluating the change in performance.

7. How do you evaluate the consistency of different methods across different technologies and platforms?

Response: We have now included in the new section “Robustness and consistency assessment” where we propose to group and analyse method performance by technologies and platforms, and from this analysis assess the consistency of the method performance.

Reviewer 1

1. The authors have conducted a comprehensive evaluation across various data types, demonstrating that method performance is highly dataset- and modality-dependent. This result is expected, as no single method can consistently outperform others in all scenarios. However, my main concern is that the primary figures only report the overall performance of each method, aggregated across all datasets. This may be misleading. I strongly recommend that the authors use a box plot or a similar visualization to allow readers to assess method performance across different datasets more intuitively.

Response: *We thank the reviewer for this suggestion. We agree that aggregated performance may obscure important dataset-specific variability. As suggested, we have added boxplots (Supplementary Figures 1–7 and Extended Data Figure2) showing the distribution of method rankings across datasets for each integration category and task, and revised the manuscript to incorporate these figure panels. These visualisations offer a clearer view of each method's performance on individual datasets, improving the interpretability of our benchmarking results.*

2. How was the performance across different datasets combined? Was it averaged? I believe aggregating performance across datasets is not ideal, as it obscures dataset-specific variations. While this paper follows a common benchmarking format, I question the effectiveness of this approach, particularly given the strong dependence of performance on dataset and modality.

Response: *We thank the reviewer for raising this important point. As noted, the overall rank scores for all datasets were summarised by first averaging the method rankings across datasets, followed by min-max scaling. While we acknowledge that aggregating performance may obscure dataset-specific variation, we adopted this approach to facilitate high-level comparison and maintain consistency with common benchmarking practices.*

To address the concern on data-specific dependence of performance, we have included in the revised manuscript boxplots in Supplementary Figures 1–7 and Extended Data Figure2, visualising the method's performance on individual datasets for each task and integration category. In addition, the performance of each method on individual datasets is also provided (e.g., Supplementary Figure 1e-g). These results allow readers to assess both overall trends and dataset-specific method performance.

3. The main figures demonstrate results from a single selected dataset. Could the authors clarify the criteria used for selecting this dataset?

Response: *We selected these specific datasets primarily because they clearly illustrate performance differences among the evaluated methods and represent diverse combinations of data modalities. This selection ensures that the chosen examples effectively facilitate interpretation and highlight key insights derived from the broader benchmarking analyses.*

4. The authors discuss MaxFuse and SMILE, but these methods were not included in the evaluation. Please incorporate them into the benchmarking analysis.

Response: *We thank the reviewer for this comment. SMILE was indeed included in the Stage 1 Registered Report and is benchmarked within our analysis of mosaic integration methods, with detailed results presented in Figure 4 and Supplementary Figure 4. Regarding MaxFuse, although it was initially listed among the considered methods, it was excluded from our benchmarking analyses based on our predefined inclusion criterion of "broad applicability" (outlined clearly in the section "Summary of integration methods in each category and task"), as MaxFuse is designed for unique data combinations such as CODEX. This is noted in Supplementary Table 2 and the last column of the main Figure 1e.*

5. I appreciate the inclusion of spatial data in the paper; however, the registration task appears somewhat disconnected from the main focus. I suggest aligning the spatial data integration strategy more closely with that used for single-cell data to ensure a more coherent analysis. Moreover, registration is only applicable to data derived from the same tissue, making it a special case rather than a broadly applicable approach in real-world studies. To improve consistency, I recommend revising the integration strategy for spatial data so that it aligns more closely with the evaluations throughout the rest of the paper.

Response: *We thank the reviewer for this comment. We acknowledge that spatial registration differs from the other evaluated tasks. Nonetheless, our decision to include it was deliberate. We view spatial location as a distinct modality, and thus consider spatial registration a valid form of modality integration. From this perspective, the task remains conceptually aligned with the central theme of the paper. To improve coherence, we have revised the manuscript to clarify this framing and better integrate the spatial registration component with the broader narrative of multimodal data integration (Section "Benchmark spatial registration methods").*

Reviewer 2

The authors have faithfully followed the benchmarking plan that was proposed in stage 1, and present a very detailed benchmarking study of the various data integration tasks for single cell analysis. The scope is impressive, both in the datasets and range of methods evaluated, and this will be a valuable resource for the community. The major results are nicely summarized in Fig9, which will be a useful guide for users in selecting methods. The discussion in appropriate, and highlights some of the limitations in the metrics and the existence of tradeoffs between different objectives (batch correction, biological conservation). The shiny app is a nice addition and can be helpful to explore the results in more detail.

Response: *We thank the reviewer for the positive and encouraging comments.*

Reviewer 3

The authors adhered closely to the experimental plan outlined in the Stage 1 submission and conducted a comprehensive benchmarking of multimodal omics integration methods across a broad collection of datasets and evaluation metrics. The analyses are accurate and statistically rigorous. I have only a few minor comments for the authors to consider:

1. Each results section begins by describing a specific dataset example, followed by a summary of the overall results and rankings. Would it be clearer to reverse this order—presenting the overall findings first, then illustrating them with representative examples?

Response: *We appreciate the reviewer's suggestion regarding the order of presentation. After careful consideration, we have opted to retain our original structure to first introduce specific dataset examples followed by the summary of overall results and rankings. We feel this format effectively guides readers through detailed examples before placing them within the broader context of the benchmarking outcomes.*

2. Related to the above, it would be helpful to clarify the criteria used to select the specific datasets featured in the main text. Were these chosen based on dataset complexity, performance variability, or other considerations?

Response: *We selected these specific datasets primarily because they clearly illustrate performance differences among the evaluated methods and represent diverse combinations of data modalities. This selection ensures that the chosen examples effectively facilitate interpretation and highlight key insights derived from the broader benchmarking analyses.*

3. On page 11, in the section "Benchmarking methods on data imputation for mosaic data analysis," the term structural similarity is used. Could the authors clarify what this term refers to in this context? Additionally, how should readers interpret the finding that structural similarity does not always align with clustering or classification performance?

Response: *We thank the reviewer for this comment. In our manuscript, "structural similarity" specifically refers to preserving intrinsic data structures, such as pairwise feature correlations, between imputed and ground-truth modalities. Practically, we quantify this using structure metrics (sMSE, pFCS, pDES) described in the Supplementary methods. The observed discordance indicates that preserving structural similarity alone does not necessarily enhance clustering or classification performance, highlighting the importance of evaluating imputation methods across multiple metrics. We have noted this in the section "Benchmark methods on data imputation for mosaic data".*

4. In the benchmarking of spatial registration methods, PASTE2—though a newer development built on PASTE—exhibited worse overall performance. Could the authors provide some discussion on why this might be the case?

Response: *We thank the reviewer for raising this point. PASTE2 was an extension of PASTE to accommodate more complex registration scenarios, such as partial alignment across more diverse spatial transcriptomics datasets. Nevertheless, the flexibility offered by PASTE2 may not provide additional advantages in aligning datasets containing adjacent or serial tissue sections where full alignment is expected. This may explain the lower overall performance of PASTE2 compared to the original PASTE in our evaluation. We have noted this in the section "Benchmark spatial registration methods".*

5. In the section "Benchmarking vertical integration methods for dimension reduction and clustering tasks," the manuscript suggests that performance on simulated datasets may not align with performance on real datasets. Does this indicate that the simulated datasets lack key characteristics of real data? If so, should their utility in benchmarking be interpreted with caution?

Response: *We appreciate the reviewer's insightful observation regarding the differences in data complexity between simulated and real datasets. We acknowledge that these differences may have resulted in the difficulty in interpreting the performance of the benchmarked methods. Accordingly, we have revised the manuscript to reflect this and to clarify that results derived from simulated data should be interpreted with appropriate caution when assessing method performance (Section "Discussion").*
